# Lower cretaceous avian-dominated, theropod, thyreophoran, pterosaur and turtle track assemblages from the Tugulu Group, Xinjiang, China: ichnotaxonomy and palaeoecology

**DOI:** 10.7717/peerj.11476

**Published:** 2021-05-28

**Authors:** Lida Xing, Martin G. Lockley, Chengkai Jia, Hendrik Klein, Kecheng Niu, Lijun Zhang, Liqi Qi, Chunyong Chou, Anthony Romilio, Donghao Wang, Yu Zhang, W Scott Persons, Miaoyan Wang

**Affiliations:** 1 State Key Laboratory of Biogeology and Environmental Geology, China University of Geoscience (Beijing), Beijing, China; 2 School of the Earth Sciences and Resources, China University of Geoscience (Beijing), Beijing, China; 3 Dinosaur Trackers Research Group, University of Colorado at Denver, Denver, United States; 4 Research Institute of Experiment and Detection of Xinjiang Oil Company, PetroChina, Karamay, China; 5 Saurierwelt Paläontologisches Museum, Neumarkt, Germany; 6 Yingliang Stone Natural History Museum, Nan’an, China; 7 Institute of Resources and Environment, Key Laboratory of Biogenic Traces & Sedimentary Minerals of Henan Province, Collaborative Innovation Center of Coalbed Methane and Shale Gas for Central Plains Economic Region, Henan Polytechnic University, Jiaozuo, China; 8 Faculty of Petroleum, China University of Petroleum (Beijing) at Karamay, Karamay, China; 9 School of Biological Sciences, The University of Queensland, Brisbane, Australia; 10 Mace Brown Museum of Natural History, Department of Geology and Environmental Geosciences, College of Charleston, Charleston, United States

**Keywords:** Tetrapod ichnofaunas, Shorebird tracks, Junggar Basin, Tugulu Group, Theropod, Thyreophoran, Pterosaur, Turtle

## Abstract

Rich tetrapod ichnofaunas, known for more than a decade, from the Huangyangquan Reservoir (Wuerhe District, Karamay City, Xinjiang) have been an abundant source of some of the largest Lower Cretaceous track collections from China. They originate from inland lacustrine clastic exposures of the 581–877 m thick Tugulu Group, variously divided into four formations and subgroups in the northwestern margin of the Junggar Basin. The large Huangyangquan track assemblages occur in the Lower layer/Subgroup II. Similarly-composed track assemblages also occur at the smaller Asphaltite site in the Upper Layer/Subgroup III. The Huangyangquan assemblages have yielded more than 1,500 identified tracks including abundant tracks of avian and non-avian theropods, pterosaurs and turtles and less abundant tracks of stegosaurs. Previous avian track identifications have been reassessed to conclude that *Moguiornipes robustus* is a taphotaxon and *Koreanaornis dodsoni* might be better accommodated in the ichnogenus *Aquatilavipes* which appears to be the dominant avian ichnotaxon. The avian track *Ignotornis* is also recognized and represents the first occurrence of this ichnogenus in China. Although the Huangyangquan assemblages lack some of the larger components (e.g., sauropodan and ornithopodan tracks) known from other Lower Cretaceous localities, the association of abundant tracks of smaller tetrapods (avian and non-avian theropods, pterosaurs and turtles) appears to be representative of lacustrine basin faunas of this region, and are an excellent example of the shorebird ichnocoenosis/ichnofacies concept. This is the first comprehensive review and re-analysis of an important Lower Cretaceous ecosystem.

## Introduction

Multiple important dinosaur faunas are known from the Early Cretaceous of China. Skeletal faunas include the Jehol Biota in northeastern China, which contains abundant psittacosaurids and feathered dinosaurs (Zhou, Barrett & Hilton, 2003), and the titanosauriform dinosaur fauna from Henan Province (Xu et al., 2012). Track faunas include assemblages from the Chabu area, Inner Mongolia (Lockley et al., 2018), the Yishu fault zone area, Shandong Province (e.g. Xing et al., 2013c, 2018c), other large, theropod-dominated, track site complexes from Shandong (Li et al., 2015; Lockley et al., 2015), the ornithopod-dominated Lotus site from Chongqing (Xing et al., 2015c) and the Sichuan Basin, all of which record faunal compositions different from that of the skeletal records (Xing et al., 2014c, 2015b; Xing & Lockley, 2016). More consistent dinosaur faunal compositions are known from skeletons and track records in the Lanzhou-Minhe Basin, Gansu Province, which both contain abundant ornithopods and sauropods (You et al., 2006; Zhang et al., 2006; Xing et al., 2013a, 2015a).

**Figure 1:**
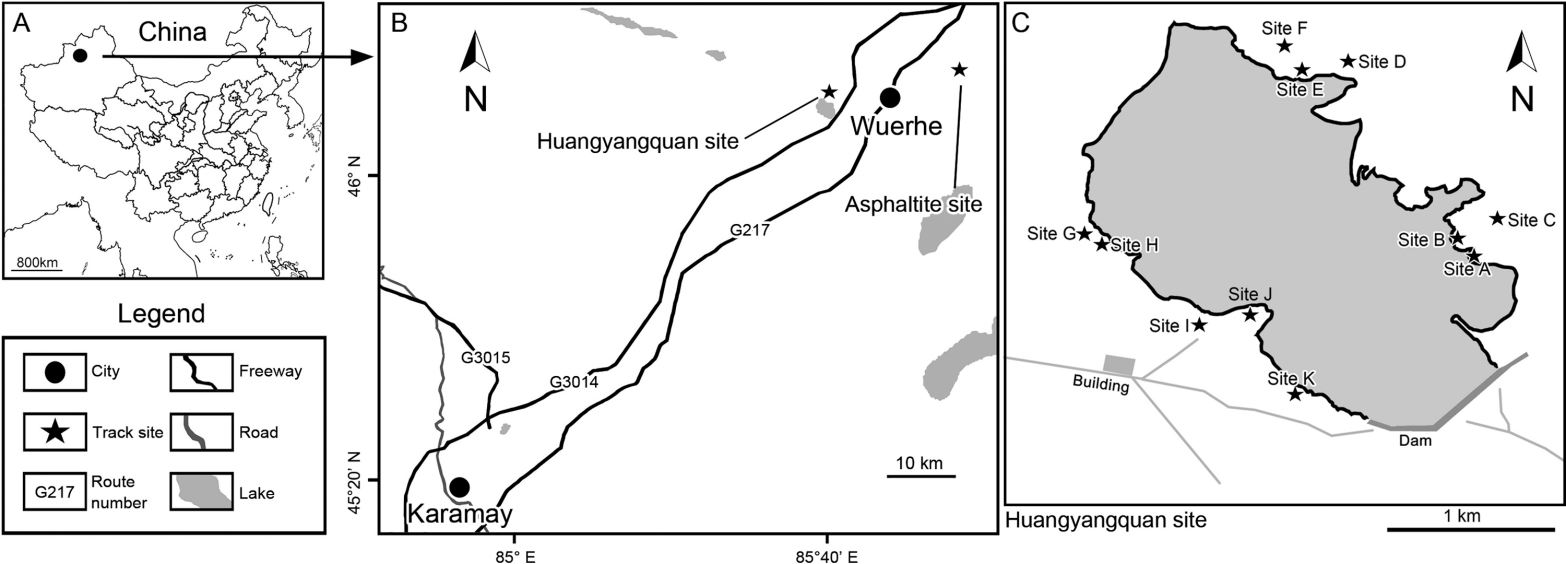
Location of the Huangyangquan track site in Xinjiang, China. (A) East Asia region; (B) Wuerhe area; (C) Huangyangquan sites (A–K). This Huangyangquan area is currently protected by the local reservoir management department. There are isolation fences around the reservoir, which protects the footprint to a certain extent.

The Lower Cretaceous Tugulu Group is widely distributed in the Junggar Basin. It is in unconformable or disconformable contact with Jurassic and Paleozoic strata, and represents a series of lacustrine deposits dominated by mudstone (Lin, 1997). The well-known Early Cretaceous *Dsungaripterus-Psittacosaurus* fauna (Dong, 1973a, 1973b; Dong, Paik & Kim, 2001) has been found in this region, but is represented by only sparse material. Xing et al. (2011) described the first bird-dominated and dinosaur track assemblages from the Huangyangquan site, in the Wuerhe area in the northwestern margin of the Junggar Basin (Fig. 1). He et al. (2013) reported large pterosaur pes tracks and bird tracks from the same site. The stegosaur tracks *Deltapodus curriei* (Xing et al., 2013d) and turtle tracks *Emydhipus* isp. (Xing et al., 2014a) are also known. Dinosaur, bird and pterosaur footprints have also been found at the Lower Cretaceous Asphaltite (=Liqingshan) site (Xing et al., 2013b).

In 2010–2012, a new expedition series, led by the main author (LX) and funded by the Key Laboratory of Vertebrate Evolution and Human Origins of Chinese Academy of Sciences, Institute of Vertebrate Paleontology and Paleoanthropology, explored the Wuerhe area. Abundant dinosaur tracks were discovered. The main authors of this paper (LX and ML) also found dense pterosaur track assemblages in 2015. In 2019, the Dinosaur Lab, from China University of Geosciences (Beijing) (LX, CC, DW), discovered a new assortment of vertebrate tracks. This manuscript will describe the new track records of the Huangyangquan and Asphaltite sites, as well as review and discuss the vertebrate ichnotaxonomy of these sites.

## Geological setting

### Tugulu group—depositional environment, stratigraphy and age

The subsidence center affecting Cretaceous strata in the Junggar Basin is located centrally within the basin, such that sedimentary thickness presents a uniform change of gradient leading to the basin edges (Cai, Chen & Jia, 2000). The Tugulu Group is a set of inland lacustrine clastic rocks and is exposed around the Wuerhe area, in the northwestern margin of the Junggar Basin, with a thickness of 581–877 m. In different regions, the group is in unconformable contact with Cretaceous and Jurassic strata, respectively. It is overlain by 1–10 m of upper Pleistocene–Holocene, alluvial and eolian sediments and poorly sorted alluvium (Academy of Prospecting & Developing, Xinjiang Bureau of Petroleum, 1977; Cai, Chen & Jia, 2000).

**Figure 2:**
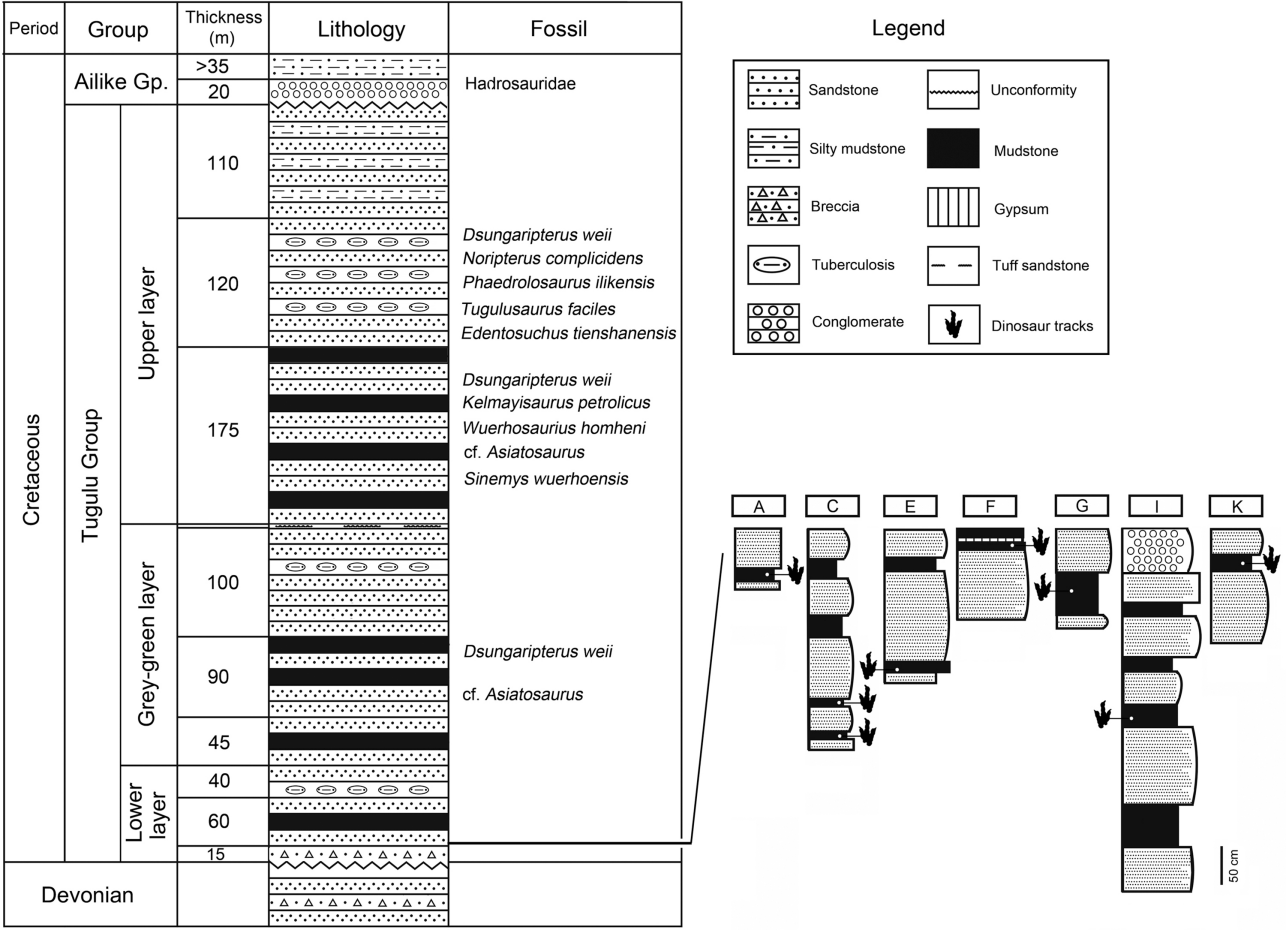
Stratigraphic section of the Lower Layer of the Tugulu Group at the Wuerhe area and Huangyangquan track sites (A, C, E, F, G, I and K) (emended from Dong, 1973a).

The subunits of the Tugulu Group are inconsistent across the Junggar Basin. The Tugulu Group is most complete at the southern margin, and can be divided into four formations in ascending order (from base to top), the Qingshuihe, Hutubihe, Shengjinkou, and Lianmuqin formations (the latter has been miss-spelled as Lianmugin in some publications). The Tugulu Group, in the northwestern margin of the Junggar Basin, can be divided into three or four subunits/ subgroups (Yang, Wang & Lu, 2008). These consist of Upper, Grey-green, and Lower layers, none of which are readily correlated with the four formations from the southern and eastern margins of the basin (Academy of Prospecting & Developing, Xinjiang Bureau of Petroleum, 1977, 1996, 1997; Dong, 1973a) (Fig. 2). Generally, the Upper layer can be compared to the Lianmuqin Formation (e.g. Maisch, Matzke & Sun, 2004). The Grey-green layer can be compared to the Shengjinkou Formation (Yang, Wang & Lu, 2008). The Lower Layer of the Tugulu Group, which has been further subdivided into eight layers (Qi, Cheng & Yang, 1995), may correlate with the better-defined, stratigraphically Qingshuihe and/or Hutubihe formations (Xing et al., 2011).

Jiang et al. (2008) divided the Tugulu Group of the Wuerhe area into four subgroups. Subgroup I is exposed on both sides of the Baiyang River, west to the Wuerhe area, and dominated by medium–thick bedded sandstone with mudstone. Subgroup II is a set of gray-green and yellow-green thick-bedded sandstone with red and brown-gray clastic rocks, exposed in the surroundings of the Wuerhe area. Subgroup III is a large set of gray-green sandstone and mudstone, interbedded with red, brown-red and reddish-brown banded mudstone, mainly exposed in the asphaltite area. Subgroup IV is a set of lacustrine gray-green sandstone and mudstone, interbedded with red, reddish brown and brown-red mudstone, exposed around the asphaltite area, east to the Wuerhe area.

Tracks from the Wuerhe area are concentrated in two regions: the Huangyangquan site and the Asphaltite site. The Huangyangquan site belongs to the Lower layer/Subgroup II of Tugulu Group (Fig. 2). The lower part of Subgroup II is characterized by gray-green, gray-yellow mega-thick and thick-bedded sandstone, with light yellow, light green or red thin-bedded mudstone. Conglomerate beds of unequal thickness are always developed in the bottom of the trough cross-bedded sandstones, which contain iron and caliche nodules. The mudstone contains secondary gypsum. The upper part of Subgroup II is light gray-green medium-thick bedded sandstone, interbedded with light gray-green sandstone with cross stratification and brown-red mudstone (Jiang et al., 2008). As shown in Fig. 2 there are 11 sites (A–K of Fig. 1) known to have yielded tracks in an area defined by the Huangyangquan reservoir of little more than 2 km^2^. As also shown in Fig. 2, multiple track bearing layers, are associated with the finer grained mudstone facies in the thin stratigraphic sequences exposed around the reservoir shore.

The asphaltite site belongs to the Upper Layer of the Tugulu Group/Subgroup III of the Tugulu Group. Trough cross beddings are always developed in the sandstone. The sedimentary environment is characterized by delta plain facies, which are superposed by delta lobes, consisting of several distributary channels, crevasse splay sand bodies, and interdistributary bay fine-grained sediments (Jiang et al., 2008).

After Jiang et al. (2008) the Tugulu Group of the Wuerhe area was mainly deposited in a shallow delta sedimentary system. Based on the particle size, the four subgroups in this region can be divided into two sedimentary cycles and show a rhythmic succession, from bottom to top, of coarse–fine–coarse–fine. This reflects a shallowing–deepening–shallowing–deepening of water levels in a lake system. The strata are mainly gray-green, and show a transition from gray-green to gray-green and red deposits, and finally to gray-green interbedded with red deposits, indicating that the depositional climate was becoming progressively drier.

The Tugulu Group is considered Lower Cretaceous, however, the definitive age of each layer is not known with certainty. Wu & Liu (1988) considered the Lower Cretaceous Tugulu Group to be roughly equivalent to the Qingshan Group of Shandong Province, the Guyang Group of Inner Mongolia, and the Jehol Group of northern Hebei and western Liaoning. Eberth et al. (2001) considered the Hutubihe Formation of to be of Hauterivian–Barremian age and the Lianmuqin Formation to be of Aptian–Albian age. Brusatte, Benson & Xu (2012) considered the Lianmuqin Formation within the Valanginian to Albian (i.e., ca. 140–99.6 Ma). Based on the *Rotellaria* Zones, Yang, Wang & Lu (2008) considered the Qingshuihe–Lianmuqin formations to be Early Cretaceous Berriasian–Barremian in age. Specifically, the Qingshuihe Formation is considered Berriasian, the Hutubihe Formation Berriasian–Valanginian, the Shengjinkou Formation Hauterivian, and the Lianmuqin Formation Barremian. Xi et al. (2019) considered the Qingshuihe Formation to be middle Berriasian–lower Valanginian, the Hutubihe Formation to be lower Valanginian–lower Barremian, the Shengjinkou Formation to be lower Barremian–lower Aptian, and the Lianmuqin Formation to be lower Aptian–middle-upper Albian.

Sedimentary features and track preservation

Tracks from Site A are located on a siltstone surface, with visible parallel bedding. There are two layers of tracks at Site C, located on the argillaceous siltstone surface. Tracks at Site E are located on nodules, and mud cracks are present on the track-bearing bedding surface. Tracks at Site F are located on siltstone, some are on nodules, and there are obvious oblique beds and interbeds of siltstone and gypsum. The track-bearing bedding surface at Site G preserves asymmetrical ripple marks, that indicate relatively weak hydrodynamic energy. There are abundant argillo-arenaceous nodules within the track-bearing siltstone of Site I, however, no tracks are preserved on the nodules. Tracks at Site K are located on the siltstone bedding surfaces, and obvious oblique bedding is present in the overlying sandstone beds. Tracks from at least three sites are preserved on nodules, and footprints may help to form the nodules by compacting the basal sediments. In addition, one pterosaur skull was discovered from a nodule at Site J, and has been interpreted as *Dsungaripterus*.

**On some surfaces footprints are associated with mudcracks.** HYQ-E-8 preserves at least four tracks directly associated with mud cracks, of which HYQ-E-8-1 and 2 are relatively well-preserved, and three claw marks of the latter are fused with mud cracks in the same direction. This is an instance of footprints helping to generate mud cracks as reported by Allen (1987). The presence of mud cracks suggests that the sediments were exposed above the water surface, and indicates cyclic wet-dry environments. HYQ-E-10 preserves four tracks with mud cracks, but without obvious evidence of the mud cracks having been directly generated by the tracks. The external morphological changes of HYQ-E-10 are stronger than HYQ-E-8, indicating wet sediments in this region. YLSNHM00978, YLSNHM00979 and YLSNHM00980 are all preserved with large-scale mud cracks. The mud crack surrounding YLSNHM00978 is 1.5 cm wide, 2 cm high, and there are relatively badly preserved *Arenicolites* or *Skolithos* in the heel, indicating the *Scoyenia* ichnofacies. There are obvious external morphological changes in YLSNHM00979. The intervals between the three toes are strongly compressed. Digit IV and the heel of YLSNHM00980 are fused with mud cracks in the same direction. There are also mud cracks fairly developed in YLSNHM01241. The cracks evidently formed earlier than the bird tracks, as in many instances the presence of the bird tracks destroyed or generated mud cracks. The most representative is YLSNHM01241-24, one ~15 cm long trackway, consisting of approximately three successive tracks. The Digit III and heels of these tracks are connected by mud cracks, but without distinct external morphological changes. Assuming a surface that dried out, to create desiccation tracks, it is possible to infer that the clearer, deeper tracks, with wider digit traces, were made first on a wetter substrate, and the shallower, tracks with thinner digit traces, were registered later on a firmer, drier substrate. This specimen (YLSNHM01241) also reveals five radial traces which are up to ~10 cm in diameter. These have a general resemblance to the deep water trace “*Glockeria*” which Książkiewicz (1977) described under the generic category of rosetted structures which includes several morphologically similar rosetted or stellate ichnogenera, including forms that may have originated in shallow or shoreline environments. Possible trace maker include worms, and arthropods.

### Invertebrate traces

**Figure 3:**
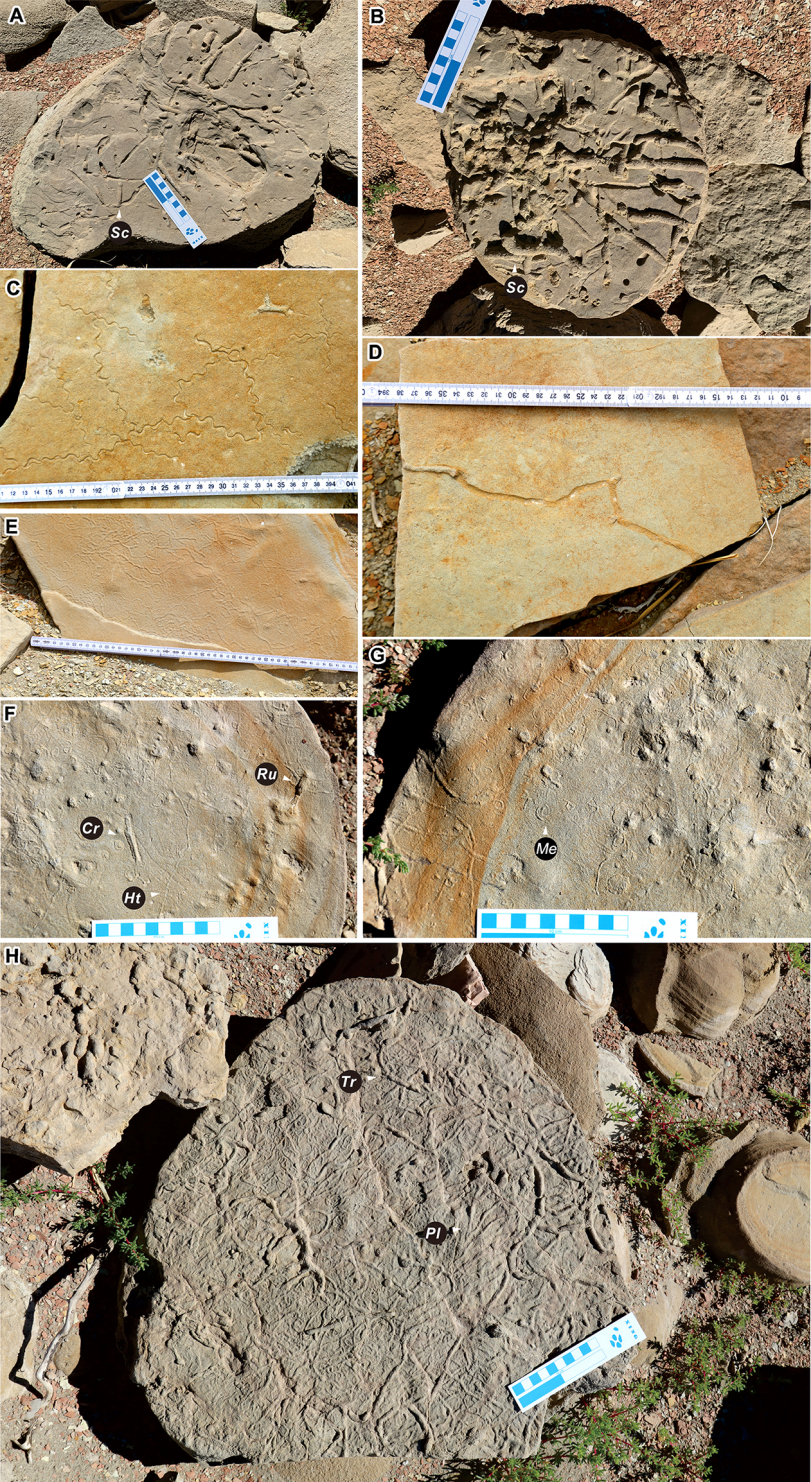
Invertebrate traces from the Huangyangquan area. *Scoyenia gracilis* (=Sc) (A, B), *Cochlichnus anguineus* (C), *Helminthoidichnites tenuis* (=Ht) (D, E, F), *Mermia carickensis* (=Me) (F, G), *Cruziana problematica* (=Cr) (F, G), *Rusophycus* isp. (=Ru) (F, G), *Treptichnus* isp. (=Tr) (H), *Planolites montanus* (=Pl) (H). Scale bar in (A), (B), (F), (G) and (H) = 10 cm; ruler in (C), (D), (E) scale in centimeter.

Invertebrate traces are abundant in the Huangyangquan area and can be divided into the several types listed below:

*Scoyenia gracilis* (Figs. 3A, 3B): Straight gently curved, unbranched burrows with meniscate spreiten. Burrows are vertical to horizontal. Commonly crossing each other, with secondary successive branching. Burrows are preserved as epichnia. Longitudinally strait lineations were observed in the wall. *Taenidium* and *Beaconites* are back-filled burrows similar to *Scoyenia*, but differ in lacking the striated lineations of *Scoyenia* (Häntzschel, 1975). *Scoyenia* is an indicator of continental ichnofacies.

*Cochlichnus anguineus* (Fig. 3C): Regularly meandering trails about 0.4 mm in diameter. Traces show a sine curve and are preserved as epichnial forms. *Cochlichnus* has been interpreted as a locomotion or grazing trace (Buatois, Mángano & Maples, 1997), probably attributable to nematodes. In a continental context, *Cochlichnus* were made near a sediment-water-air interface, and are common in lake shoreline deposits.

*Helminthoidichnites tenuis* (Figs. 3D, 3E, 3F): These are irregularly winding horizontal, mostly smooth ribbons or grooves in the upper bedding surface.

*Mermia carickensis* (Fig. 3G): Very thin, 0.1 mm wide, consists of densely looped grooves. Some segments show a winding course. In fresh water, *Mermia* is interpreted as locomotion trials produced by worms.

*Cruziana problematica* (Fig. 3F): Straight to winding, hypichnial, double ridges, divided by a distinct median furrow, and probably produced by arthropods.

*Rusophycus* isp. (Fig. 3F): Small bilobate mounds with parallel lobes, preserved in convex hyporelief. Also reported in fluvial and shallow lacustrine soft-ground media, and probably produced by arthropods.

*Treptichnus* isp. (Fig. 3H). Subhorizontal burrow consisting of one series of J-shaped segments. Segments connected in a zigzag irregular pattern near their ends.

*Planolites montanus* (Fig. 3H): Small, unlined, curved to contorted, horizontal burrows, preserved as semireliefs. Burrow diameter ranges between 1 and 2 mm. It is interpreted as an infaunal grazing trace produced by an annelid or other type of worm (Pemberton & Frey, 1982).

**Figure 4:**
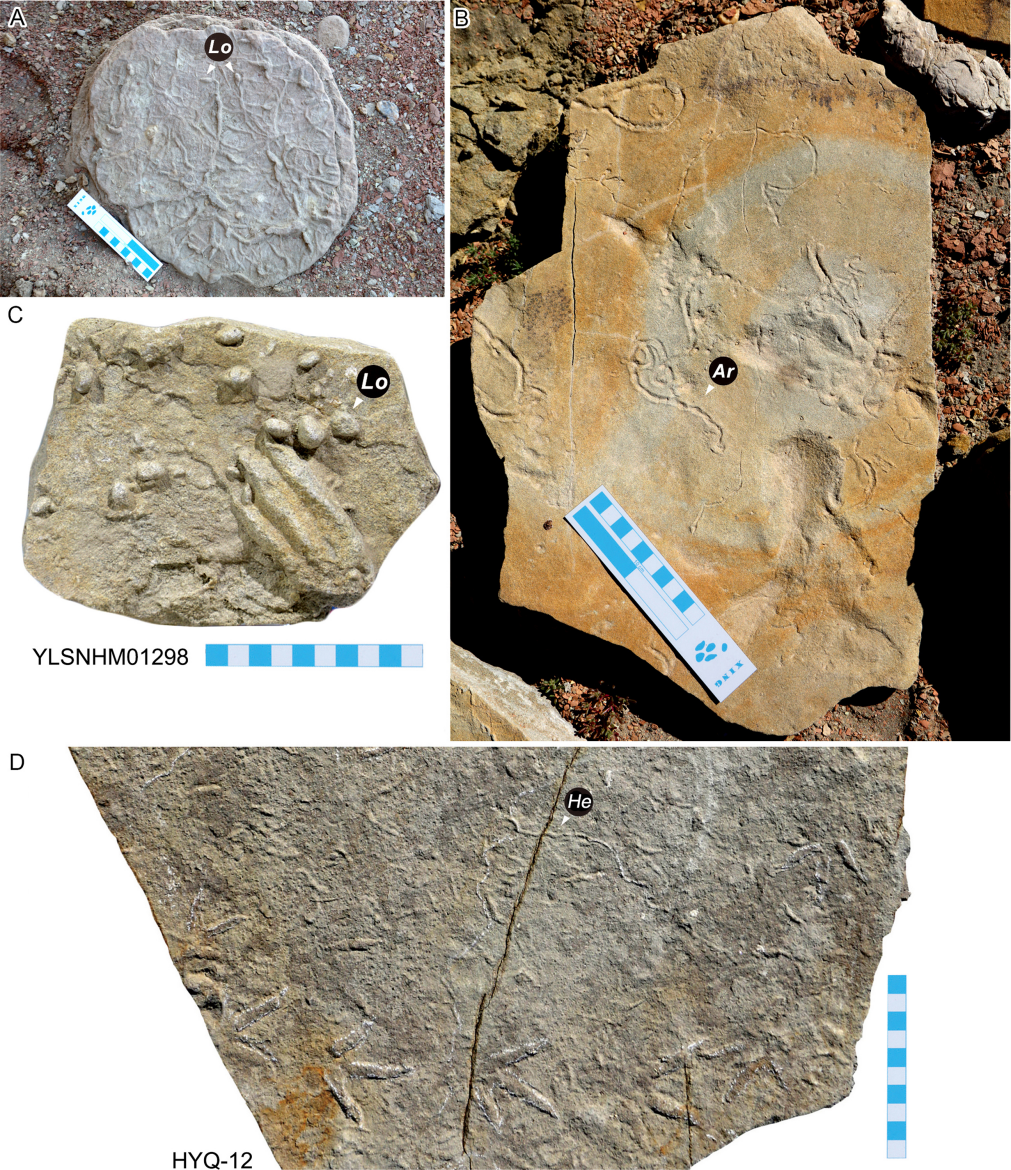
Invertebrate traces from the Huangyangquan area. *Lockeia siliquaria* (=Lo) (A, C), *Archaeonassa fossulata* (=Ar) (B) and *Helminthopsis abeli* (=He) (D). Scale bar = 10 cm

*Lockeia siliquaria* (Figs. 4A, 4C): Almond-shaped traces preserved as convex hyporelief tarping at both ends. Surface commonly smooth. *Lockeia* is attributed to the burrowing activity of bivalves (Osgood, 1970).

*Archaeonassa fossulata* (Fig. 4B): Occurring as positive epireliefs, with two convex parallel lateral levees.

*Helminthopsis abeli* (Fig. 4D): Irregularly winding, unbranched, horizontal burrows, with massive filling. No crosscutting. Preserved as epichnial forms. *Helminthopsis* has no loops (Hofmann & Patel, 1989), whereas *Helminthoidichnites* displays only occasional loops and, in *Gordia*, loops are the most characteristics feature.

Radiating traces in YLSNHM01227 and YLSNHM01241: Circle-shaped, radiating burrows with numerous arms (radii) preserved in convex hyporelief.

This trace fossil assemblage is assigned to the *Scoyenia* ichnofacies, which may be distributed in floodplains, ponds, lake margins, ephemeral lakes and humid interdunes. Considering sedimentary characteristics, the track-bearing bedding is interpreted as a shallow lacustrine and nearshore lacustrine deposit (Gu et al., 2003; He et al., 2013) based on (1) common interbeds of sandstone and mudstone, (2) abundant silty components, (3) common parallel beddings or oblique beddings (indicating higher energy hydrodynamic environments), (4) rare ripple marks, (5) developed mud cracks, (6) nodules (arenopelitic) with abundant bioturbation structures (including invertebrate traces and vertebrate tracks), (7) uncomplicated lithology with only sandstone, mudstone, and very few conglomerate interbeds, and (8) nearly parallel or horizontal bedding.

## Materials & methods

### Huangyangquan area

The Huangyangquan Reservoir is located 15 km northwest of Wuerhe District, Karamay City, and was completed in 1991. This rectangular reservoir is ~2.74 km long from northwest to southeast, and ~1.74 km from northeast to southwest, with a circumference of 9.67 km. There are at least 11 track sites in this region, designated Site A–K, with a total of 608 reported tracks, including non-avian and avian theropod, stegosaur, pterosaur and turtle tracks. Sites A and C are dominated by turtle tracks, Site B is dominated by stegosaur tracks, Sites D–I, and K are dominated by non-avian theropod tracks, Site J is dominated by pterosaur tracks.

### Collection

The collection of dinosaur tracks in the Wuerhe area has been accomplished in two stages. In the first stage, which occurred before 2011, isolated specimens were collected by the local Moguicheng Dinosaur and Bizarre Stone Museum (MGCM), most of these were discovered around Huangyangquan, although specific locality information was not recorded. They have been described by Xing et al. (2011). In the second stage our team has focused on stratigraphic information, furthermore type specimens of *Deltapodus curriei*, which are original natural molds from in situ horizons, were discovered (Xing et al., 2013d). In 2019, due to space and funding constraints, most MGCM specimens (including tracks and skeletons) were moved to the Yingliang Stone Natural History Museum, Shuitou, Fujiang Province, China. New specimen numbers were issued (prefixed with YLSNHM, see Supplement Information 1 for the list). Beginning in 2015, investigations were conducted along the margin of the Huangyangquan Reservoir, and all specimens collected on the original horizons were prefixed with HYQ. These HYQ specimens were reposited in YLSNHM, as well as the new tracks discovered during 2015–2019 investigations that are described herein.

## Methods

According to the standard methods of Leonardi (1987) and Lockley & Hunt (1995), the maximum length, maximum width, pace length, stride length, pace angulation and rotation of the dinosaur tracks were measured. The track length, width, pace length, pace angulation stride length, outer trackway width, and inner trackway width were measured for pterosaur trackways based on the method of Xing et al. (2013e). The methods of Olsen (1980), Weems (1992), and Lockley (2009) were used to measure the mesaxony of tridactyl tracks. Mesaxony refers to the degree to which the central digit (III) protrudes anteriorly beyond the medial (II) and lateral (IV) digits.

The HYQ-7 trackway and HYQ-E-11 track were photographed using a Canon EOS (5D Mark III) digital camera from different viewpoints in a series of overlapping images under artificial lighting conditions. A scale-corrected, digital surface model (resolution = 0.141 mm/pix) was created from methods adapted from Falkingham et al. (2018) by adding images to Agisoft Metashape Professional Edition (version 1.5). Another ex situ fossil specimen YLSNHM00974 was photographed using an iPhone 6 digital camera (4.15 mm lens) from different viewpoints under artificial lighting conditions. A scale-corrected, digital surface model (resolution = 0.149 mm/pix; 44.8 points/mm^2^) was also created by Agisoft. Both 3D digital models were then positioned to the center of the cartesian coordinate system using Meshlab (64bit_fp v2016.12; Cignoni et al., 2008), and visualized using the ambient occlusion filter in CloudCompare (v2.6.1 64 bit; www.cloudcompare.org). False-colour elevation and contour maps utilized filters in Paraview (version 5.0.0 64 bit; Ahrens, Geveci & Law, 2005) following the procedure adapted from Xing et al. (2018c).

Interpretive outline tracings were made using transparency film covering the tracks and then digitalized with a vector-based drawing software.

## Results

### Non-avian theropod tracks

#### Description

**Figure 5:**
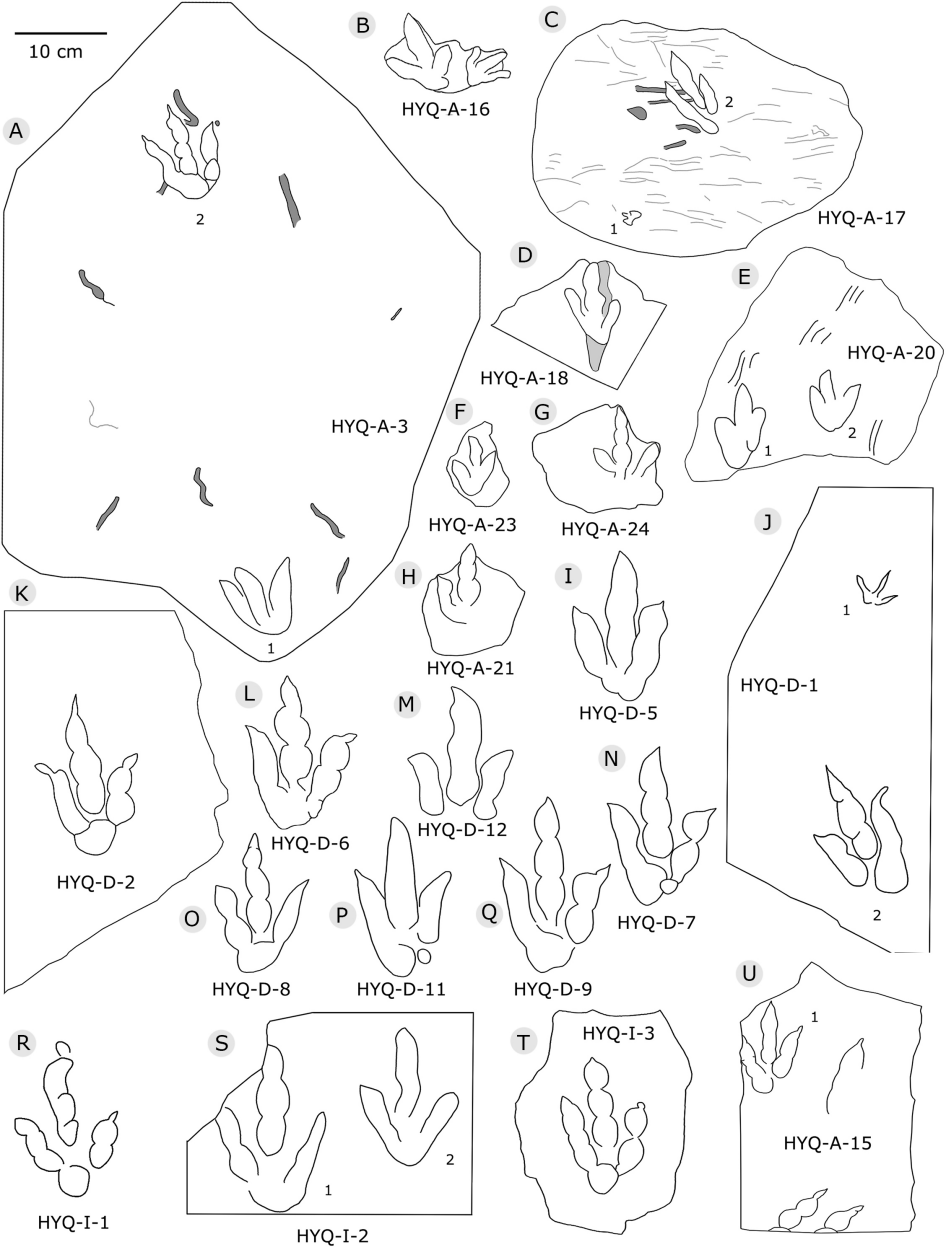
The interpretive outline drawing of theropod tracks at Huangyangquan sites: HYQ-A-3 (A), 16 (B), 17 (C), 18 (D), 20 (E), 23 (F), 24 (G), 21 (H); HYQ-D-5 (I), 1 (J), 2 (K), 6 (L), 12 (M), 7 (N), 8 (O), 11 (P) 9 (Q); HYQ-I-1 (R), 2 (S), 3 (T), and HYQ-A-15 (U).

**Figure 6:**
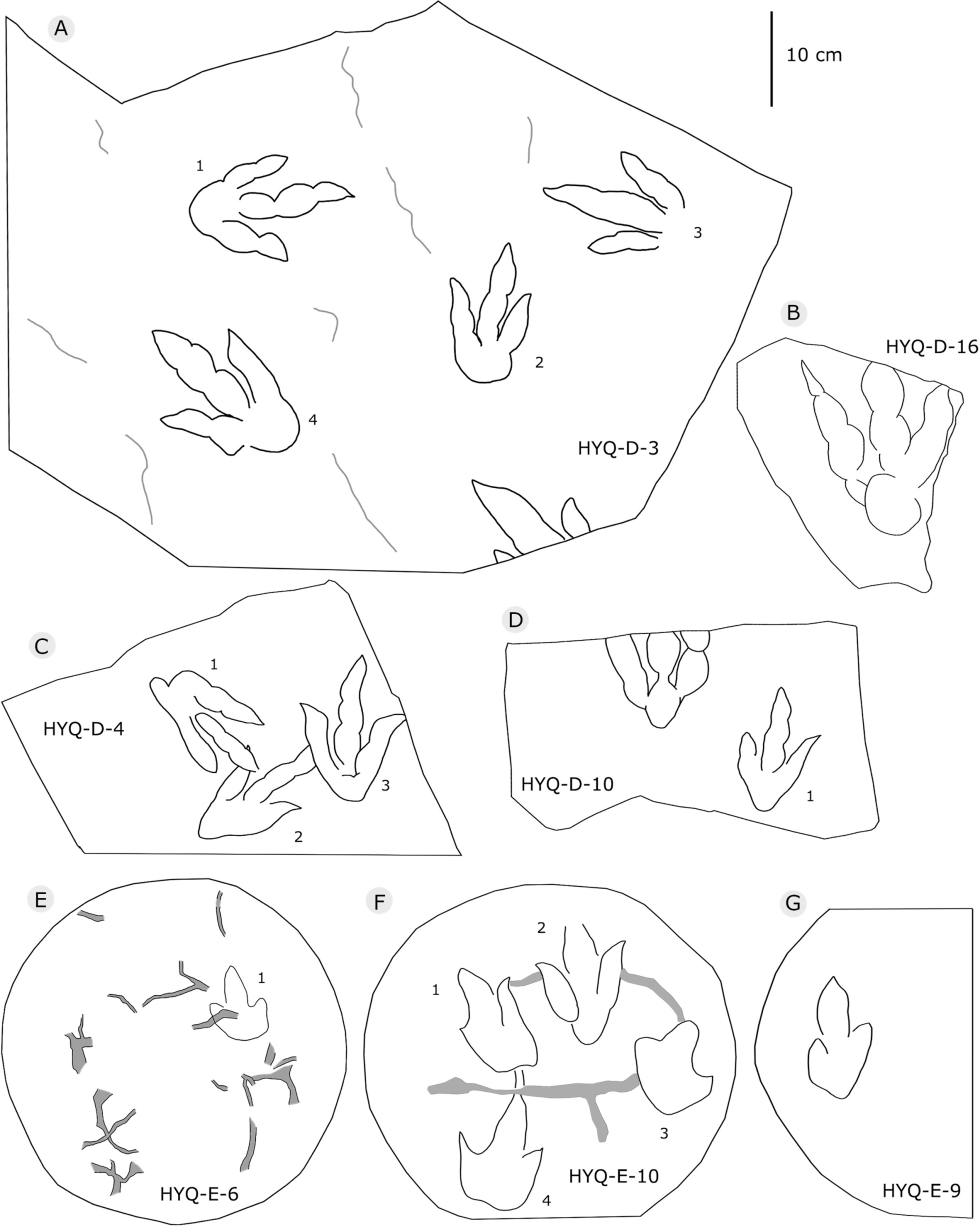
The interpretive outline drawing of theropod tracks at Huangyangquan sites: HYQ-D-3 (A), 16 (B), 4 (C), 10 (D); HYQ-E-6 (E), 10 (F), 9 (G).

**Figure 7:**
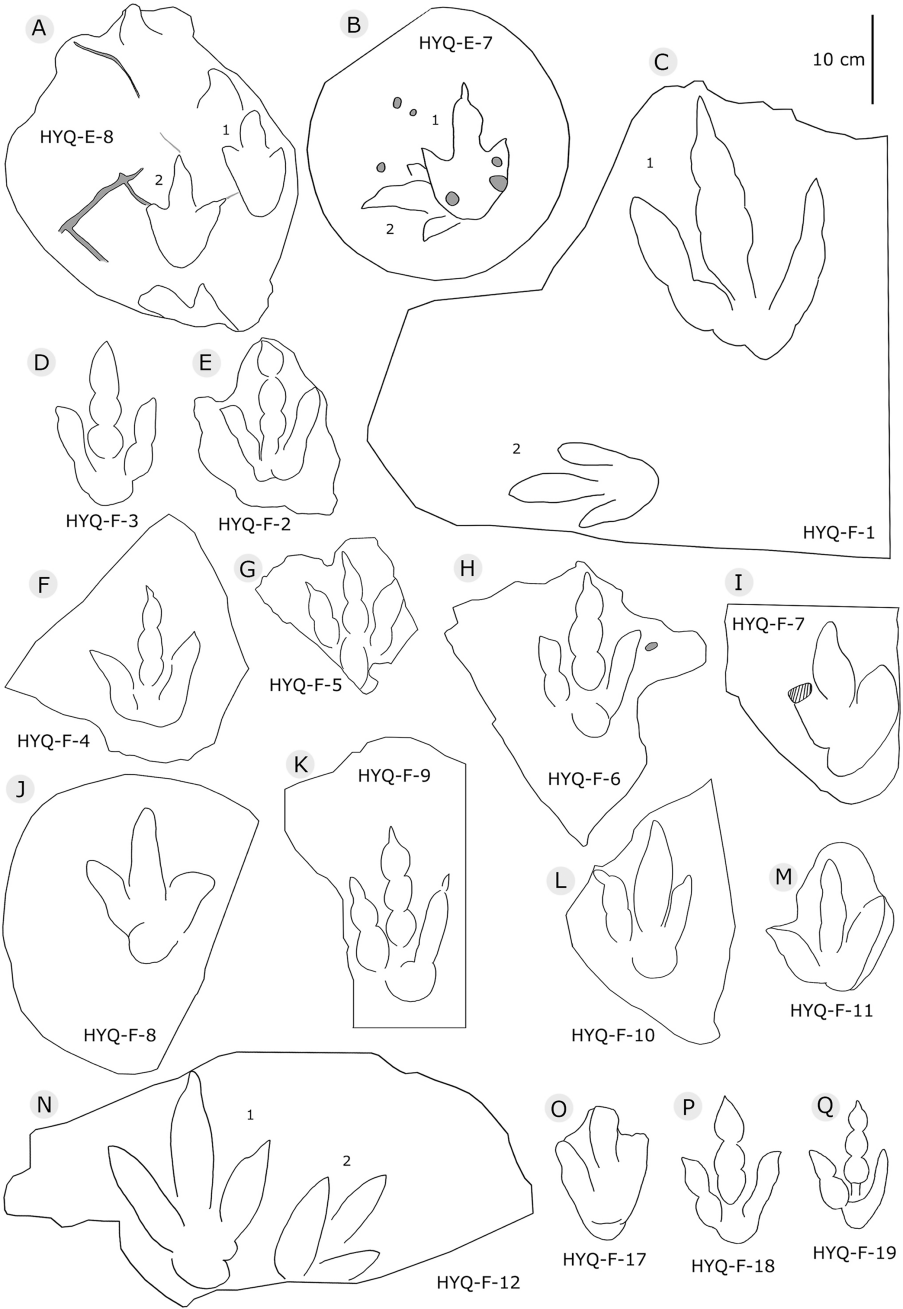
The interpretive outline drawing of theropod tracks at Huangyangquan sites: HYQ-E-8 (A), 7 (B); HYQ-F-1 (C), 3 (D), 2 (E), 4 (F), 5 (G), 6 (H), 7 (I), 8 (J), 9 (K), 10 (L), 11 (M), 12 (N), 17 (O), 18 (P), 19 (Q).

**Figure 8:**
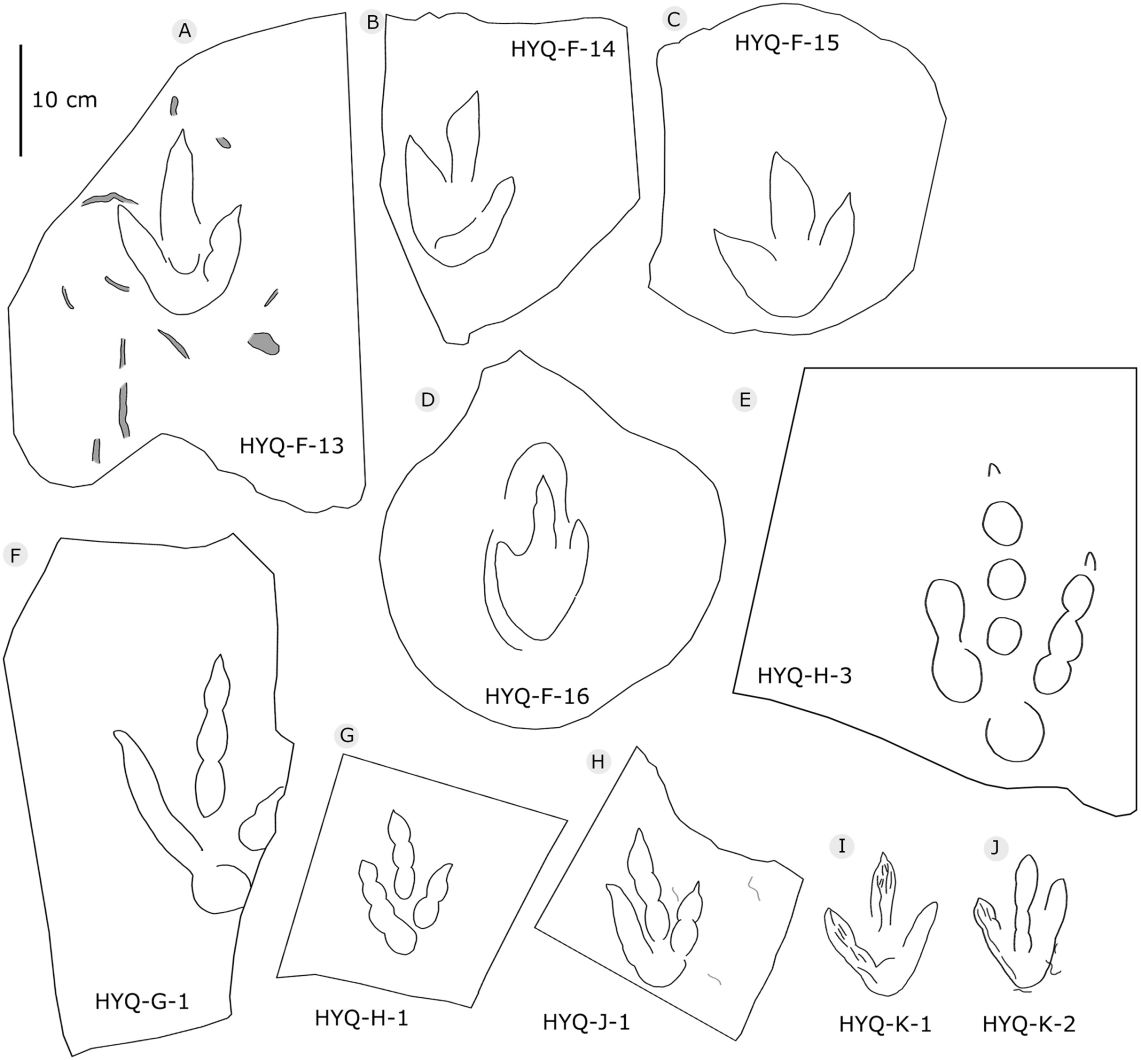
The interpretive outline drawing of theropod tracks at Huangyangquan sites: HYQ-F-13 (A), 14 (B), 15 (C), 16 (D); HYQ-H-3 (E); HYQ-G-1 (F); HYQ-H-1 (G); HYQ-J-1 (H); HYQ-K-1 (I), 2 (J).

**Figure 9:**
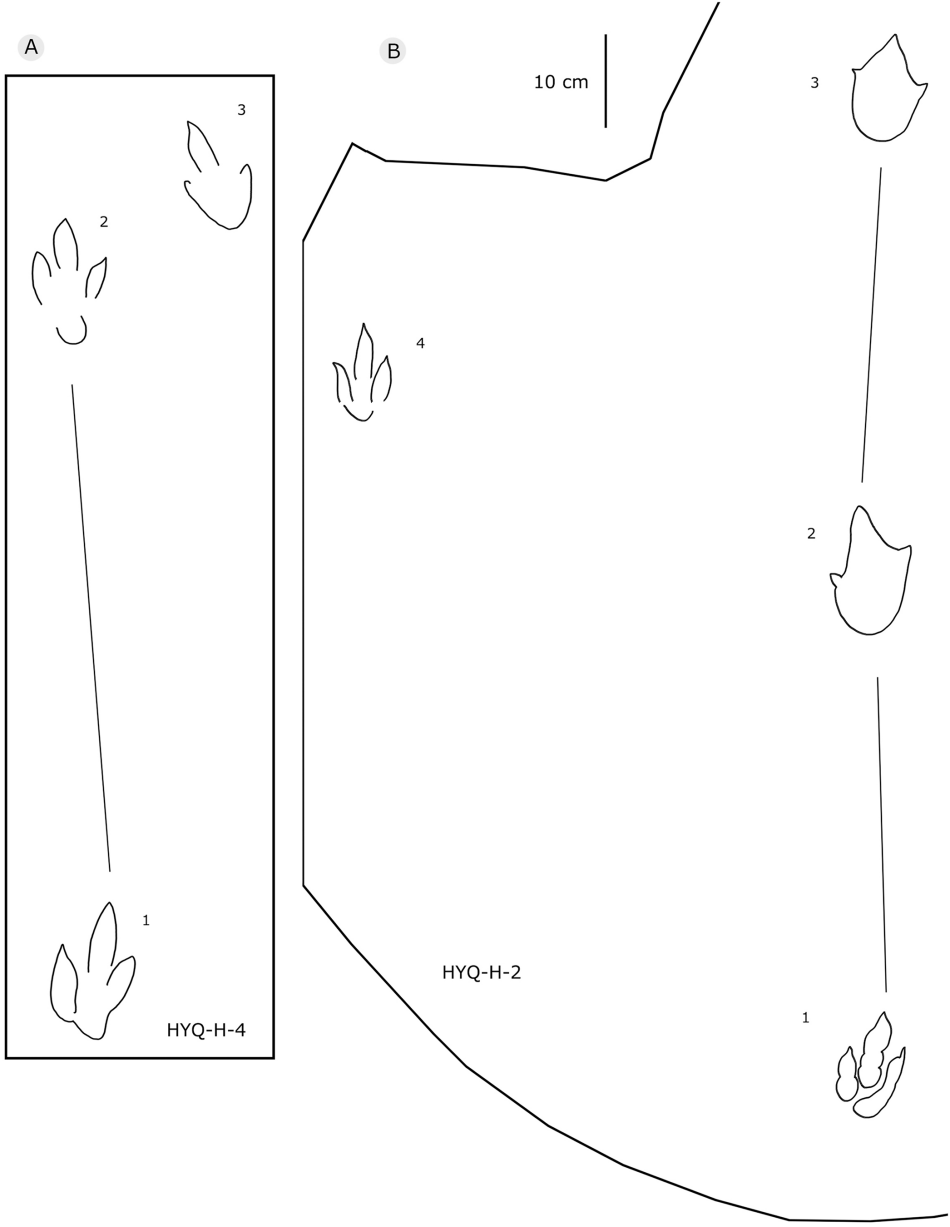
The interpretive outline drawing of theropod tracks at Huangyangquan sites: HYQ-H-4 (A), 2 (B).

**Figure 10:**
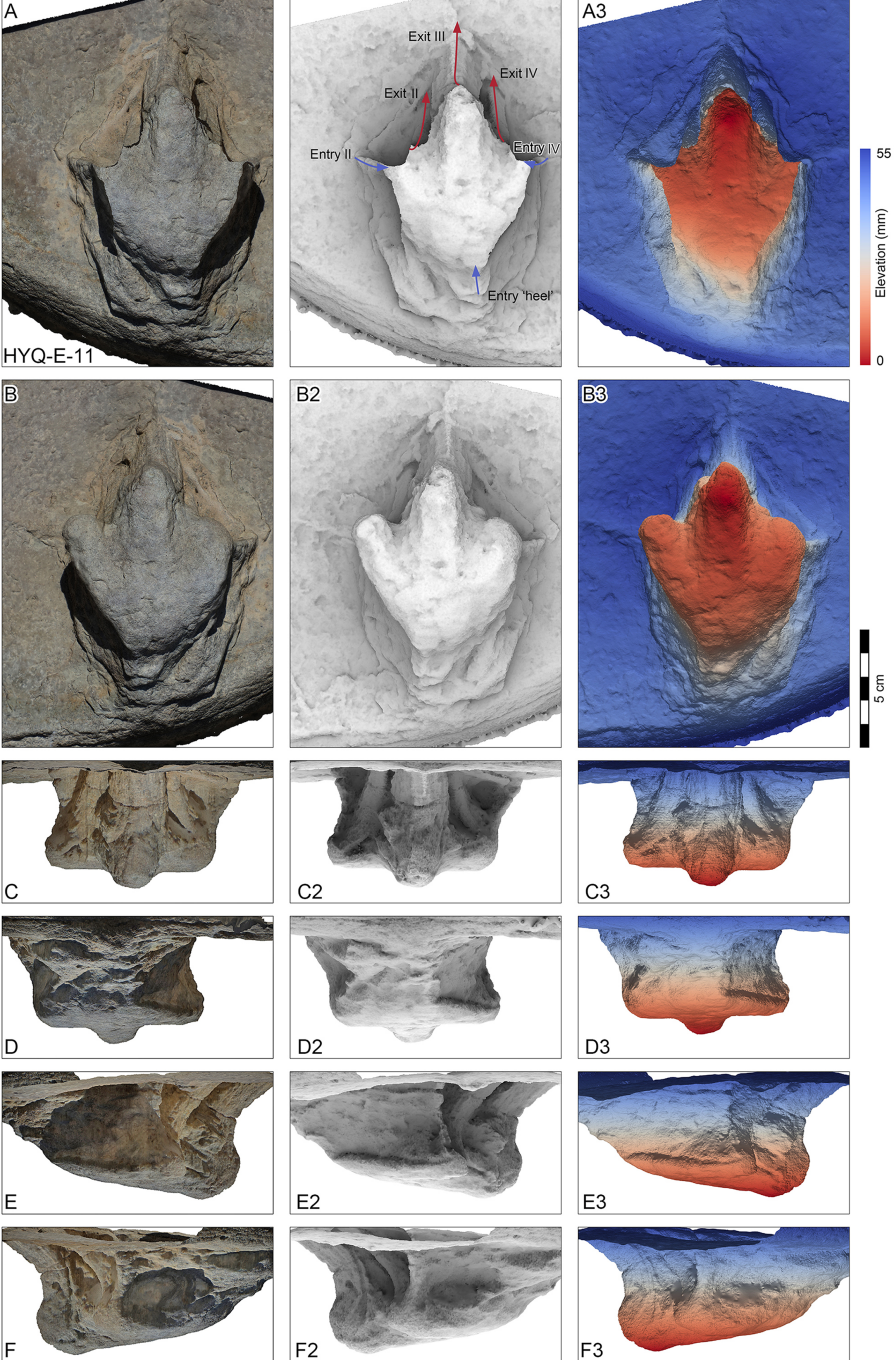
Perspective of HYQ-E-11 (left pedal impression). (A) Dorsal view; (B) Ventral view; (C) Anterior view; (D) Posterior view; (E) Medial view; and (F) Lateral view. The track surface images are shown as orthophotographs, ambient occlusion images, and elevation images.

**Figure 11:**
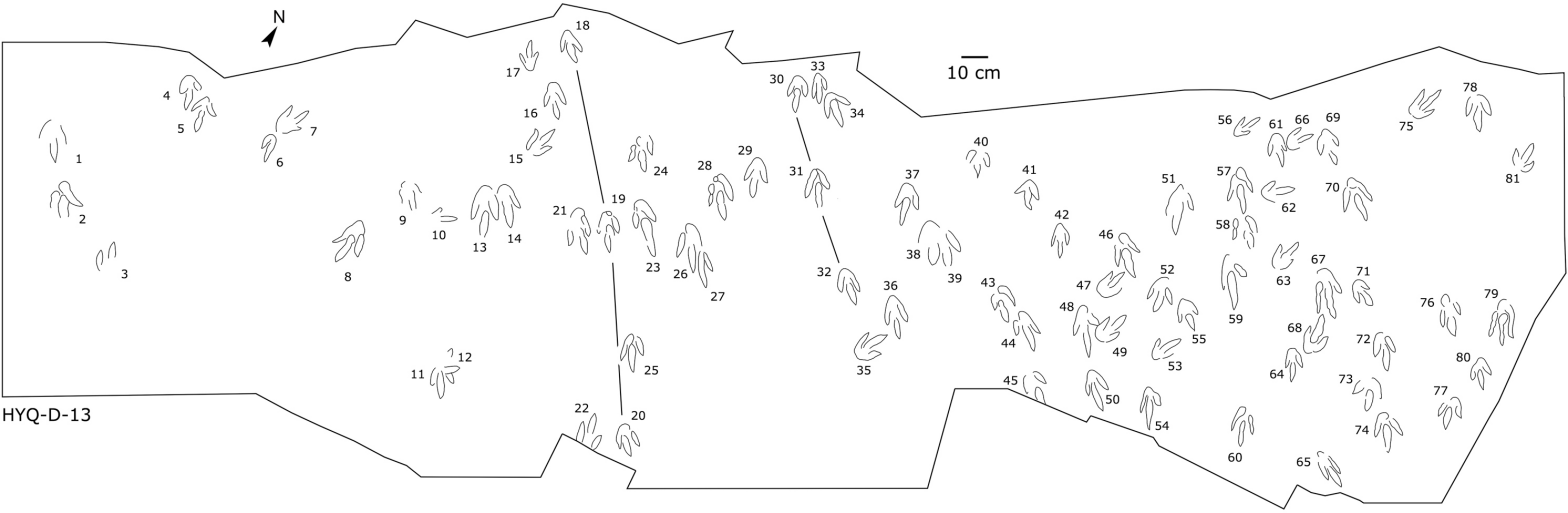
The interpretive outline drawing of theropod tracks at Huangyangquan sites: HYQ-D-13.

**Figure 12:**
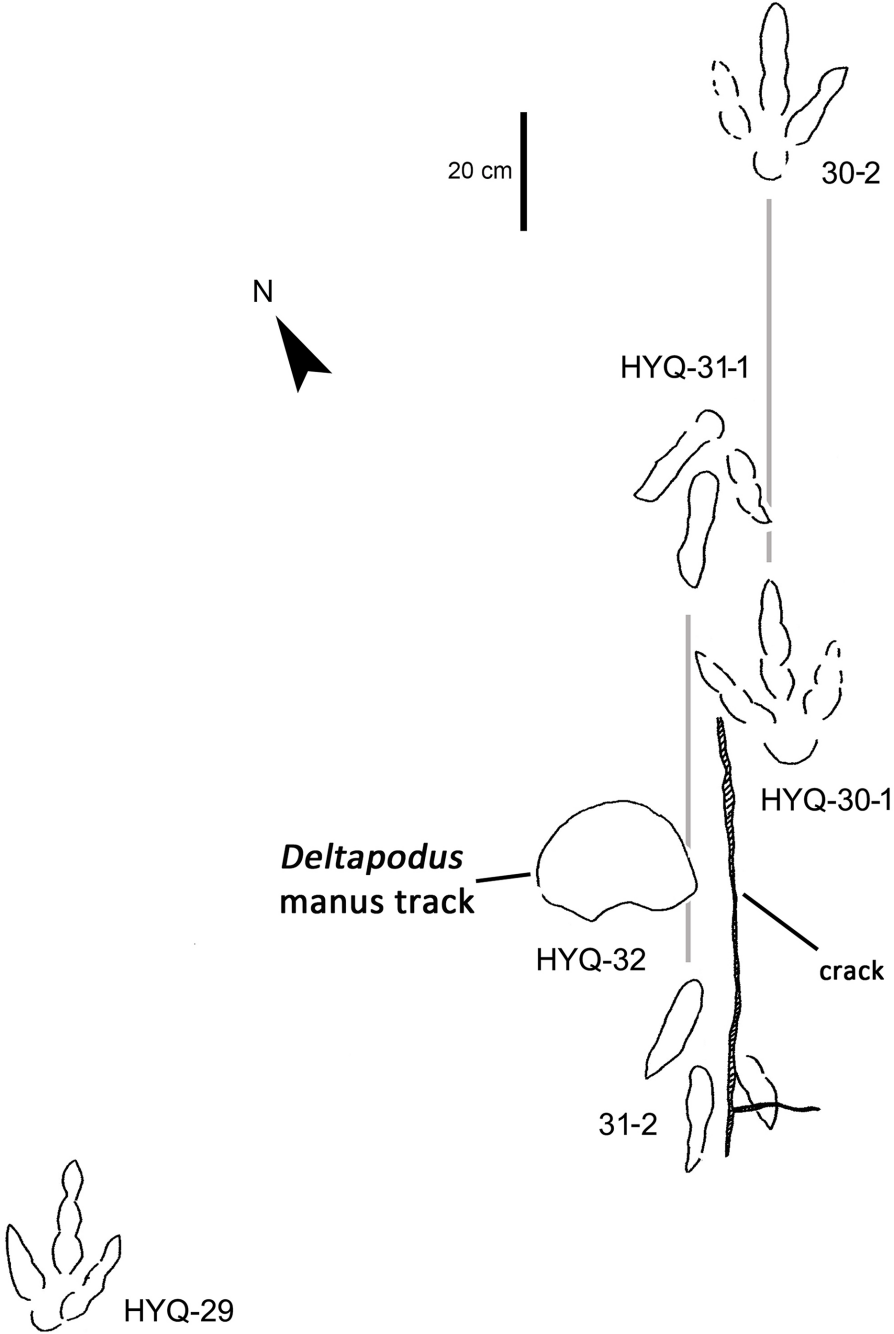
The interpretive outline drawing of theropod tracks at Huangyangquan sites: HYQ-29, 30-1, 30-2, 31-1, 31-2, 32.

**Figure 13:**
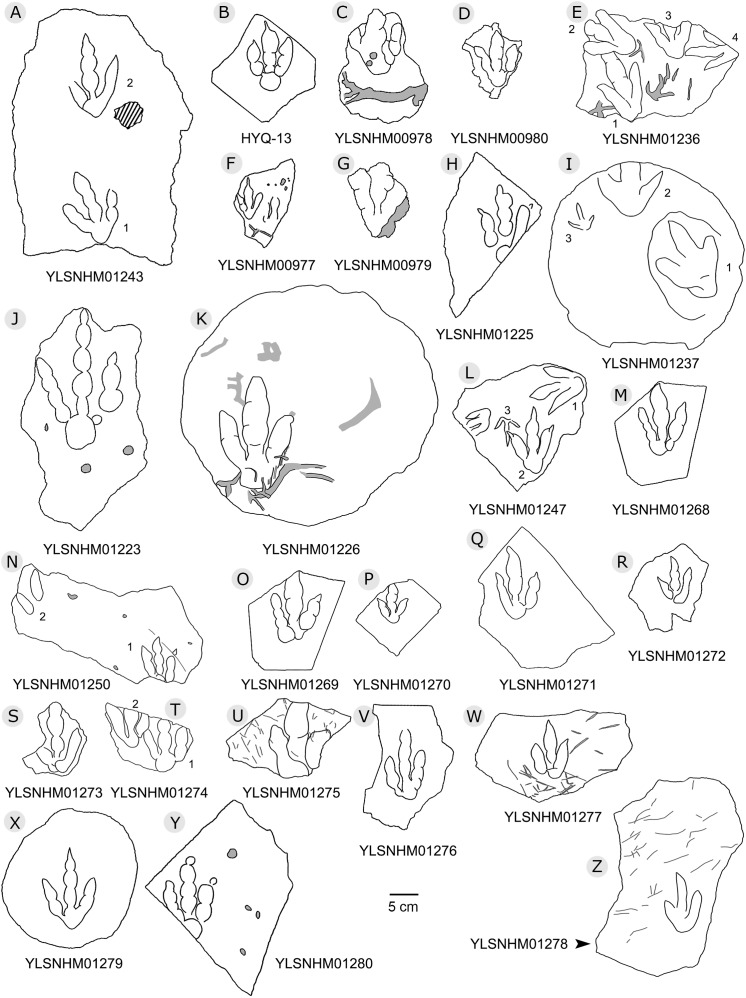
The interpretive outline drawing of theropod tracks at Huangyangquan sites. Specimens numbers: YLSNHM01243 (A); HYQ-13 (B); YLSNHM00978 (C), 00980 (D), 01236 (E), 00977 (F), 00979 (G), 01225 (H), 01237 (I), 01223 (J), 01226 (K), 01247 (L), 01268 (M), 01250 (N), 01269 (O), 01270 (P), 01271 (Q), 01272 (R), 01273 (S), 01274 (T), 01275 (U), 01276 (V), 01277 (W), 01279 (X), 01280 (Y), 01278 (Z).

**Figure 14:**
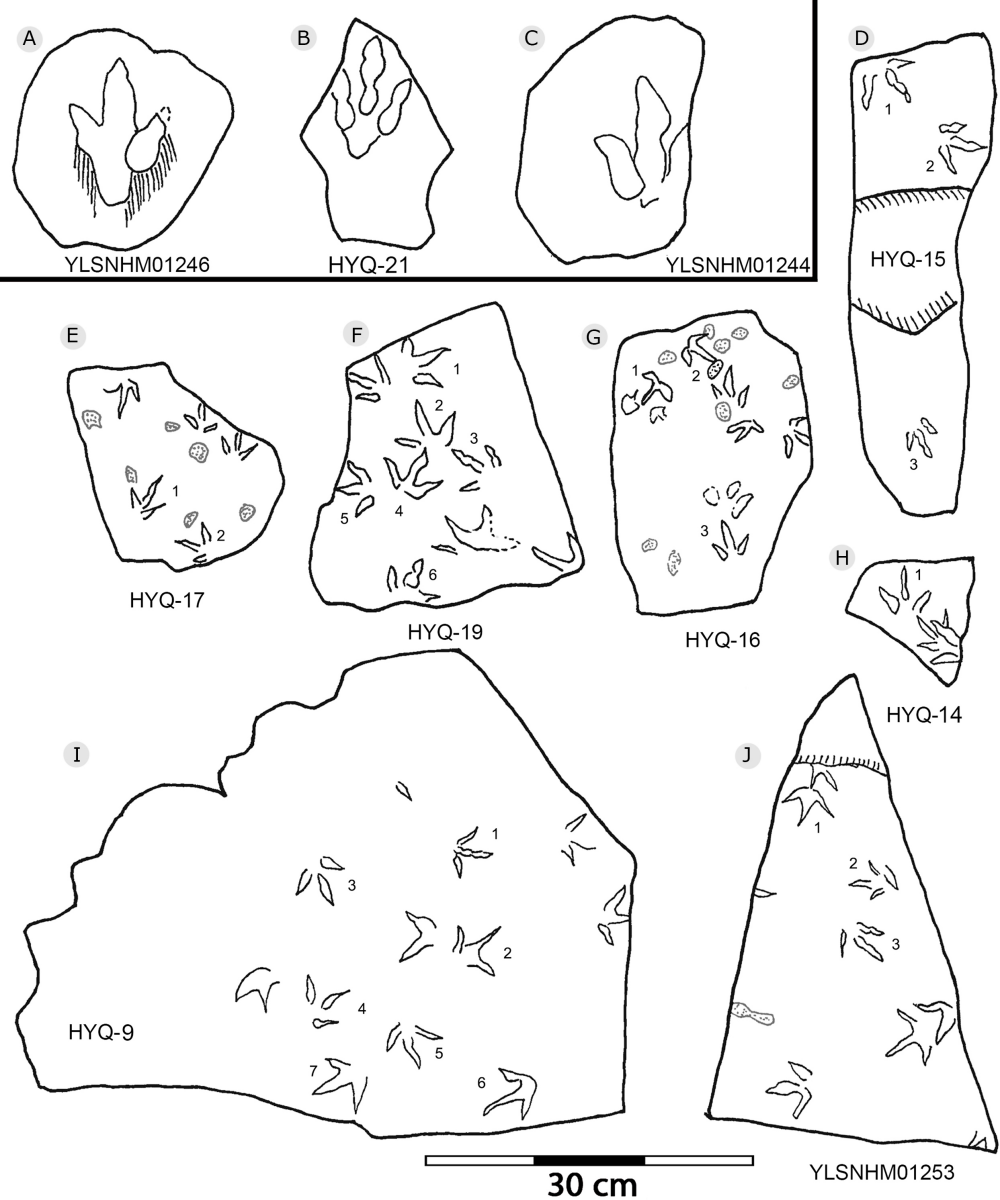
The interpretive outline drawing of theropod and bird tracks at Huangyangquan sites: YLSNHM01246 (A); HYQ-21 (B); YLSNHM01244 (C); HYQ-15 (D), 17 (E), 19 (F), 16 (G), 14 (H), 9 (I); YLSNHM01253 (J).

**Figure 15:**
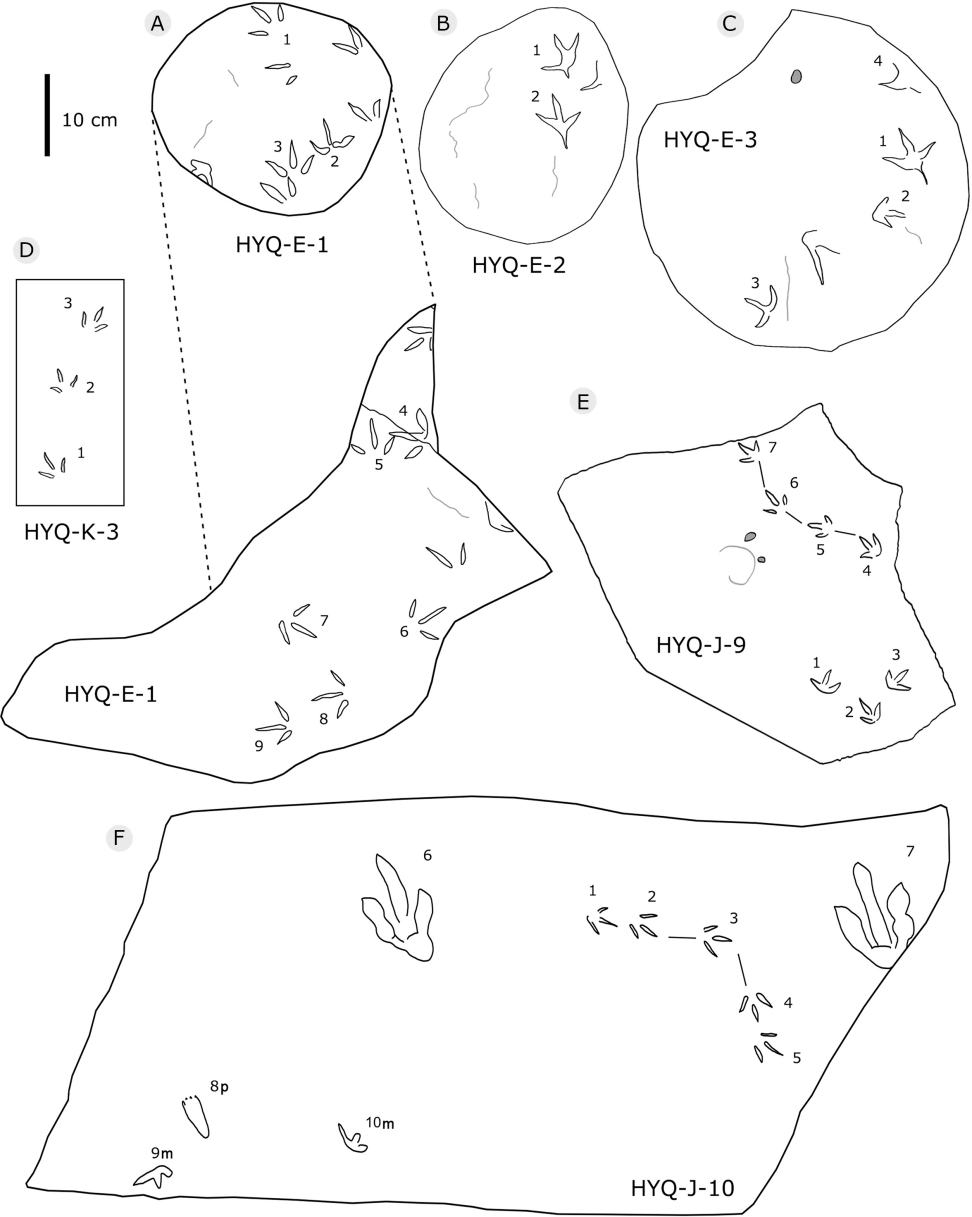
The interpretive outline drawing of theropod and bird tracks at Huangyangquan sites: HYQ-E-1 (A), 2 (B), 3 (C); HYQ-K-3 (D); HYQ-J-9 (E), 10 (F).

**Figure 16:**
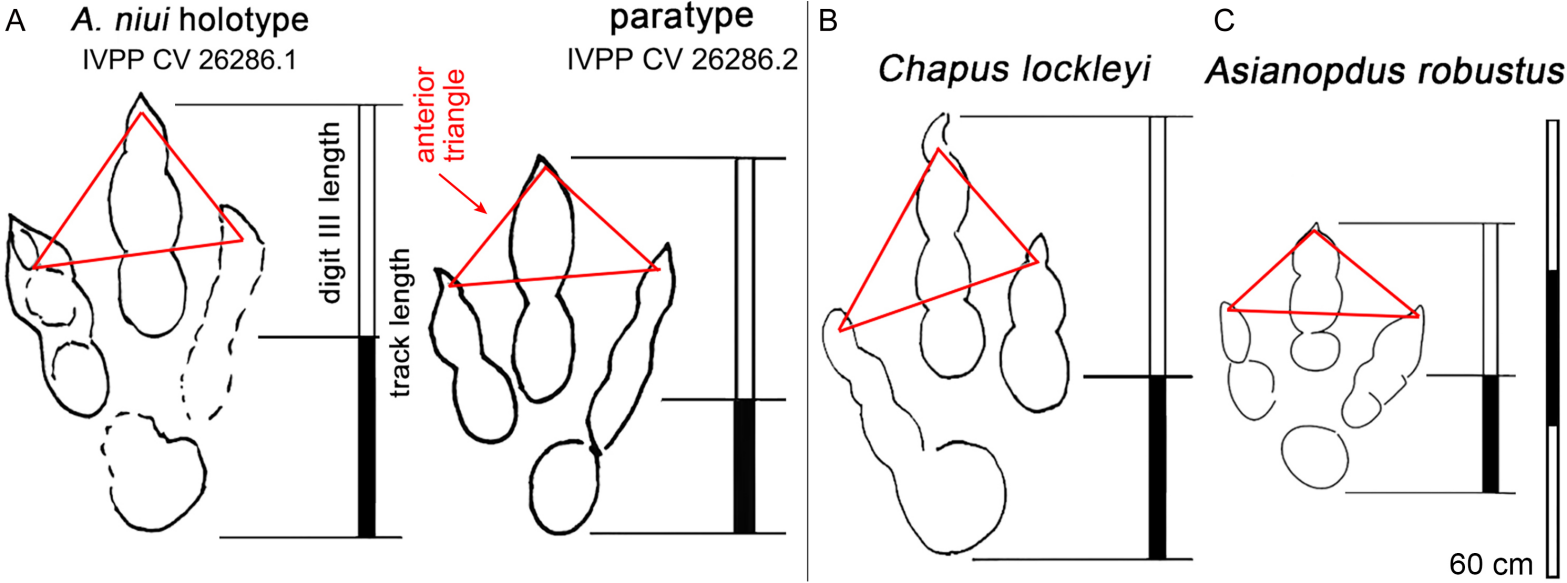
*Asianopudus niui*. Comparisons between holotypes of *Asianopudus* and *Chapus* from China. (A) *A. niui*, (B) *C. lockleyi* and (C) *A. robustus*, showing anterior triangle and ratio of digit III/Footprint length. Note similarities between *A. niui* and *C. lockleyi*. See text for details.

**Figure 17:**
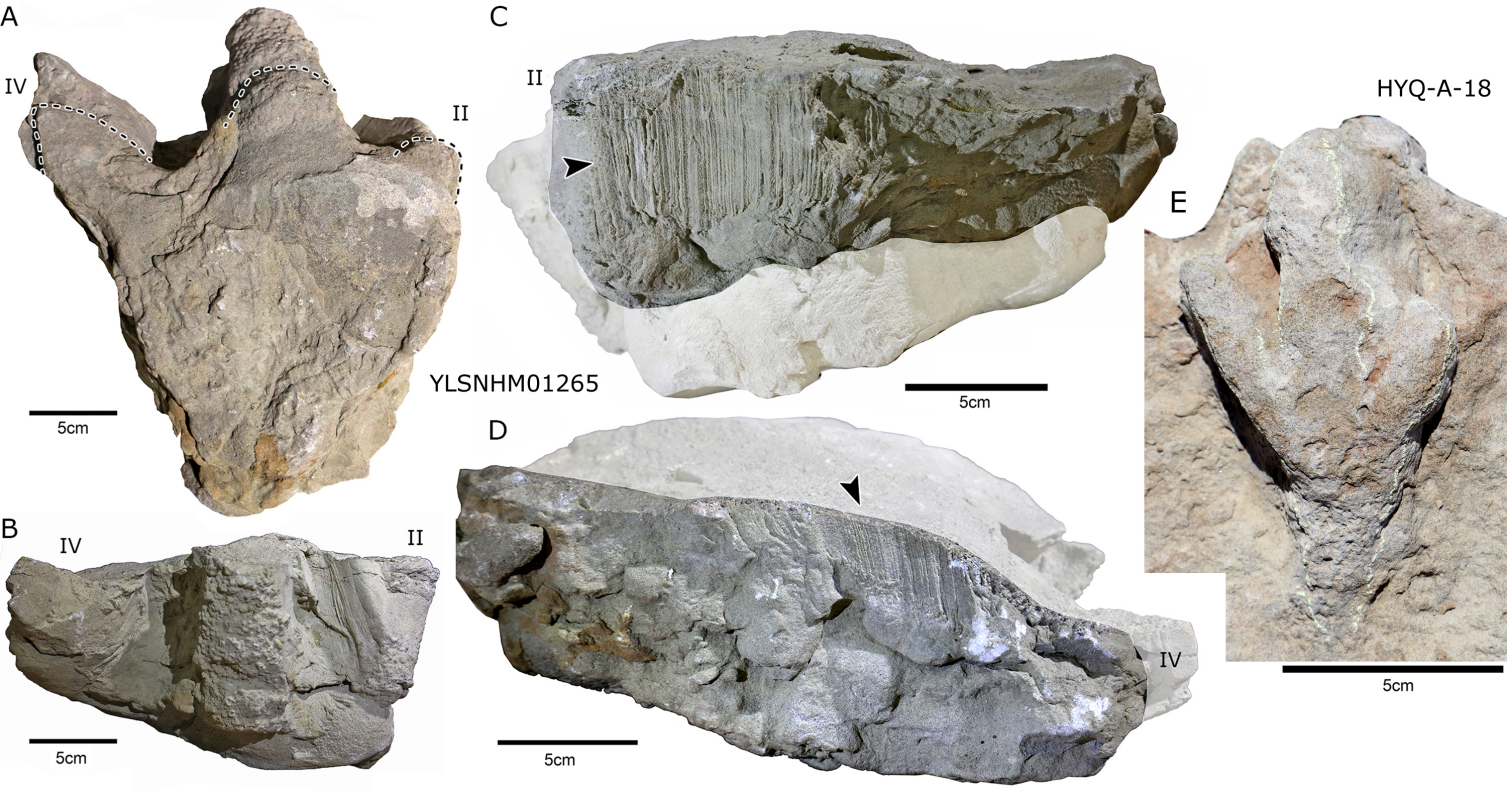
Photograph of theropod track YLSNHM01265 and HYQ-A-18 at Huangyangquan site: Dorsal (A), Anterior (B), Lateral (C) and Medial (D) views of YLSNHM01265; Dorsal (E) view of HYQ-A-18. The black arrow indicate the scale scratch lines along the side.

**Figure 18:**
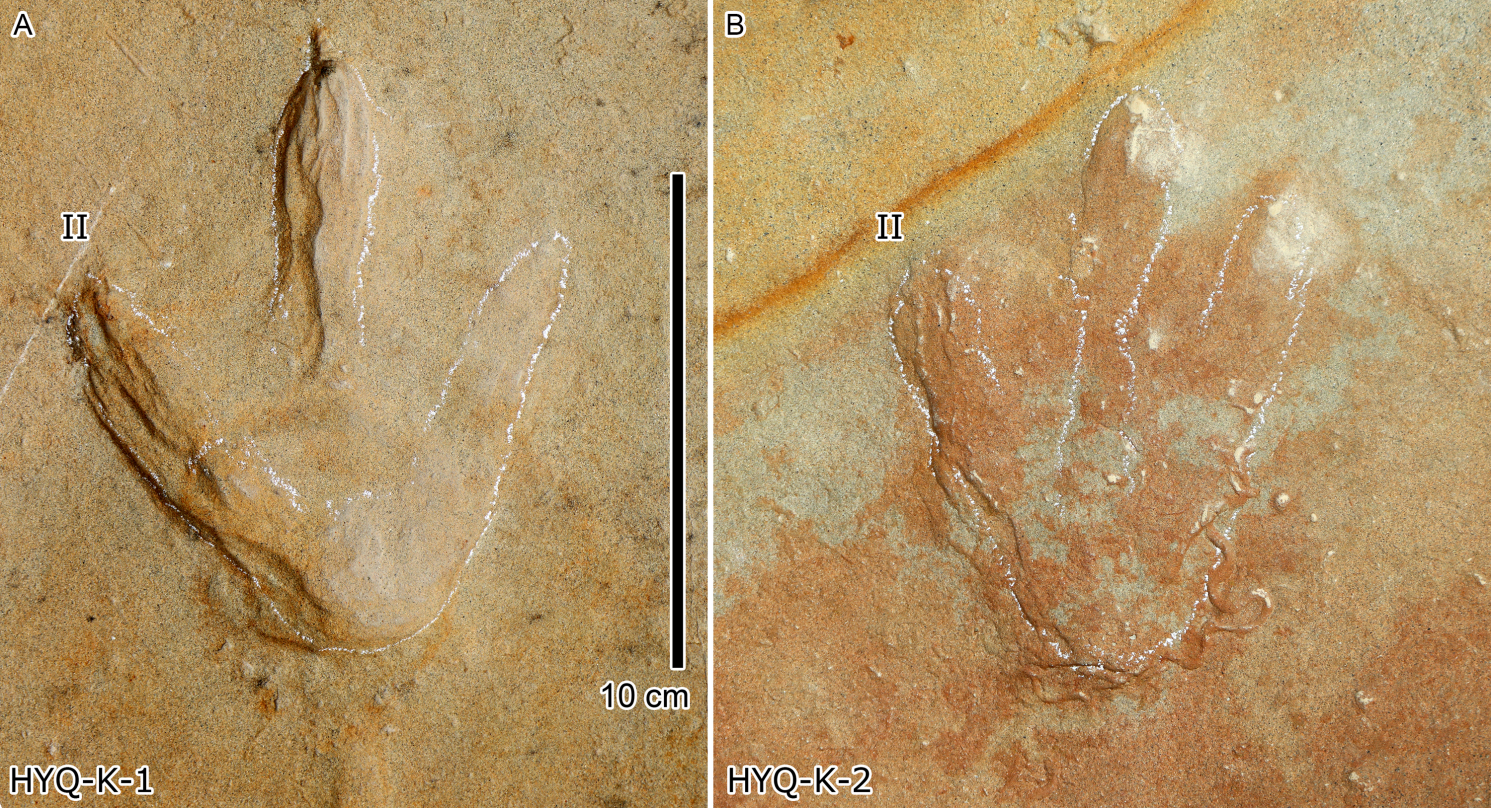
Photograph of theropod track HYQ-K-1 (A) and 2 (B) at Huangyangquan site.

In total 202 non-avian theropod tracks were counted in the Huangyangquan area (see Table 1 for detailed list, and Supplement Information 2 for the measurements) (Figs. 5–18), and therefore represent about one third (202/608 = 33.2%) of the total track sample recorded to date. Of the 176 well-preserved tracks, the smallest is 5.3 cm in length, and the largest is 31.5 cm in length. These tracks fall into four size categories: <10 cm, 16 tracks; 10–19 cm, 147 tracks; 20–29 cm, 10 tracks; >30 cm, 3 tracks. So, the vast majority (163/176 = 92.6%) are less than 20 cm in length, and can be labelled as grallatorid. Within the interval of 10–19 cm, 51 tracks fall within the range of 10.7–13.4 cm and almost as many (50) tracks fall within the range of 13.5–16.1 cm. The length/width ratios vary from 1.1 to 3.0. Twelve tracks have a length/width ratio of 1.1–1.2, 50 tracks have a ratio of 1.3–1.5, 70 tracks have a ratio of 1.6–1.9, and 24 tracks have a ratio of 2.0–3.0. With regards to mesaxony 26 tracks fall in the range of 0.36–0.47, 40 in the range of 0.48–0.58, 43 in the range of 0.59–0.69, and 21 in the range of 0.7–0.8. Based on these measurements and other morphological traits, the theropod tracks can be coarsely divided into three morphotypes. These morphotypes are not necessarily identical to different ichnotaxa. They can also represent extramorphological (substrate-related) variations of the same ichnotaxon, or be part of a size (growth)-related continuum. They might even indicate possible synonymies of ichnotaxa that have formerly been considered as distinct. Hereafter we describe and discuss these morphotypes and their possible ichnotaxonomic relationships without giving a formal assignment.

**Table 1:** Comparison of the type material of the monospecific ichnogenus *Chapus* and the three ichnospecies of *Asianopdus*.

	Track			Tm.	Theropod			Bird			Stegosaur			Pterosaur			Turtle		
	To.	Is.	Tw.		Ra.	Is.	Tw.	Ra.	Is.	Tw.	Ra.	Is.	Tw.	Ra.	Is.	Tw.	Ra.	Is.	Tw.
Huangyangquan site																			
Field site A	237	235	1	236	5.5%	12	1	–	–	–	–	–	–	36.9%	87	0	57.6%	136	0
Field site B	20	20	0	20	–	–	–	–	–	–	100.0%	20	0	–	–	–	–	–	–
Field site C	54	54	0	54	–	–	–	–	–	–	–	–	–	–	–	–	100.0%	54	0
Field site D	105	99	2	100	97.0%	95	2	1.0%	1	0	2.0%	3 (2)*	0	–	–	–	–	–	–
Field site E	55	55	0	55	20.0%	11	0	45.5%	25	0	–	–	–	29.1%	16	0	5.5%	3	0
Field site F	21	21	0	21	100.0%	21	0	–	–	–	–	–	–	–	–	–	–	–	–
Field site G	1	1	0	1	100.0%	1	0	–	–	–	–	–	–	–	–	–	–	–	–
Field site H	9	4	2	6	100.0%	4	2	–	–	–	–	–	–	–	–	–	–	–	–
Field site I	4	4	0	4	100.0%	4	0	–	–	–	–	–	–	–	–	–	–	–	–
Field site J	97	79	4	83	3.6%	3	0	7.2%	4	2	–	–	–	89.2%	72	2	–	–	–
Field site K	5	2	1	3	66.7%	2	0	33.3%	0	1	–	–	–	–	–	–	–	–	–
Total A-K	608	574	10	583	–	153	5	–	30	3	–	22	0	–	175	2	–	193	0
Li, Jiang & Wang, 2020	15	4	3	7	–	4	3	–	–	–	–	–	–	–	–	–	–	–	–
Museum	929	867	17	883	–	45	3	–	557	11	–	29 (28)	2	–	91	1	–	145	0
Total	1552	1445	30	1473	14.5%	202	11	40.8%	587	14	3.5%	50	2	18.3%	266	3	22.9%	338	0
Asphaltite site																			
Total	44	44	0	44	61.3%	27	0	36.4%	16	0	–	–	–	2.3%	1	0	–	–	–

**Notes:**

* Indicates the number of trackmakers, such as “2 (1)”, a set of pes and manus traces left by one trackmaker.

(To), Total; (Is), Isolated; (Tw), Trackway; (Tm), Trackmaker; (Ra), Ratio. Numbers under Li, Jiang & Wang, 2020 refer to data from this paper; numbers under “Museum” refer to museum specimens.

Morphotype A consists of tracks shorter than 20 cm, and is best represented by the well-preserved tracks HYQ-D-2, HYQ-D-9, HYQ-I-1, and HYQ-I-3 (Figs. 5, 13 in part). The length/width ratio ranges between 1.3 and 1.9, the divarication angles range between 36° and 72°, and the mean length/width ratio of the anterior triangle ranges between 0.47 and 0.8. The phalangeal pad formula is x-3-3-4-x (including metatarsophalangeal pads II and IV) or x-2-3-4-x (excluding metatarsophalangeal pad II). All the digits preserve sharp claw marks. There is a distinct interphalangeal space between the proximal pad I of digit III and the metatarsophalangeal pad of digit IV. The distance between the proximal ends of digits II and III and the metatarsophalangeal region is apparently larger than that between the proximal end of digit IV and the metatarsophalangeal region. An elongate and swollen metatarsophalangeal area consists of the larger metatarsophalangeal pad of digit IV and a smaller, medial metatarsophalangeal pad behind digit II, likely belonging to digit II. The proximal (metatarsophalangeal) area of the footprint is larger than any individual digital pad trace.

Only a few trackways of morphotype A are preserved, each consists a single step or stride. The mean pace length of five such trackways is 4.6 times (3.0–5.4 times) the length of the footprint. The trackways are very narrow, with an average pace angulation of 170° (166°–175°), and are rotated inwards towards or outwards from the axis of the trackway, the latter is more common. For example, three tracks, HYQ-H-2-1–3 (Fig. 9), are clearly rotated ~16° outwards from the axis of the trackway, while tracks HYQ-D-13-18–20 (Fig. 11) are rotated ~8° outwards from the axis of the trackway, and tracks HYQ-D-13-30–32 are rotated ~17° inwards from the axis of the trackway. A heel is absent in HYQ-D-12 (Fig. 5), which is rarely seen in the Huangyangquan area, and the interdigital divarication between digit II and digit IV is therefore extended to 78°.

Morphotype B comprises tracks that are 26.1–31.5 cm in length, e.g. represented by HYQ-F-1-1, HYQ-H-3, HYQ-29, 30 and 31 (Figs. 6 in part, 7, 8, 12, 13 in part). HYQ-H-3 (Fig. 8) is well preserved as a natural mold (concave epirelief) and shows a typical tridactyl form. The length is 26.1 cm and the length/width ratio is 1.8. The

typical theropod pad configuration is visible, with 2, 3, and 4 digital pads corresponding to digits II, III and IV respectively. Distal claw traces are also clearly visible in digits III and IV. The metatarsophalangeal pad is located axially posterior to the axis of digit III and is well-developed and sub-round. The total divarication angle between digits II and IV is 45°, and the divarication angle between digit II and digit III is 20° being less than between digit III and digit IV which is 25°. The length/width ratio of the anterior triangle is 0.51.

HYQ-30 and HYQ-31 (Fig. 12) tracks with lengths of 30–31.5 cm are poorly preserved, however, relatively large sub-round heels are present in some tracks, such as HYQ-30-1. These tracks are similar to HYQ-H-3 in morphology, but with a lower length/width ratio (~1.3). The mean length/width ratio of the anterior triangle in HYQ-30 and HYQ-31 tracks is 0.46–0.58. The pace lengths of HYQ-30 and HYQ-31 are 3.2 and 3.3 times the length of the footprint.

Morphotype C consists of 12 tracks with a length/width ratio of 1.1–1.2 and a length of 7.7 cm–25.2 cm. Most of them are poorly preserved and lack distinct pad impressions. They may represent an extramorphological (substrate-related) variation of Morphotype A. The most representative is HYQ-E-11 (Fig. 10), which is 7.7 cm in length, has a length/width ratio of 1.2. The divarication angle is 70° between digits II and IV. The length/width ratio of the anterior triangle is 0.50.

### Comparison & discussion

Based on limited specimens, Xing et al. (2011) assigned theropod tracks from the Huangyangquan site to: cf. *Jialingpus* isp. (six complete natural casts on a single slab cataloged as MGCM.H1–4, 7, and 8), *Asianopodus* isp. (one isolated specimen, MGCM.H6), and *Kayentapus* isp. (one isolated specimen, MGCM.H5). Xing et al. (2014b) reviewed the *Jialingpus* specimens from China, including the type material of *Jialingpus yuechiensis*, and Dijiaping and Bawangzhuang specimens from the Ordos Basin. In general *Jialingpus* reflects a small to medium-sized theropod trackmaker which registered grallatorid tracks akin to *Grallator* (*sensu lato*). However, a distinctive, centrally placed, swollen metatarsophalangeal area is a distinctive and diagnostic feature of well-preserved *Jialingpus*, making it different from *Eubrontes*–*Grallator*–*Anchisauripus* plexus tracks. In the best-preserved specimens of *Jialingpus*, a crease separates the small metatarsophalangeal pad region behind digit II and the larger region behind digit IV. This trait is clearly present in the theropod morphotype A tracks from the Huangyangquan site, which can, therefore, be assigned to *Jialingpus*.

*Jialingpus* trackways are rare. Only two pairs of footprints represent possible steps in the type material of *Jialingpus* (Zhen, Li & Zhen, 1983) where the mean pace length is 4.5 times (3.6 and 5.4 times) the length of the footprint. The Huangyangquan morphotype A tracks allow credible comparisons with *Jialingpus* tracks and trackways, the pace length being 4.6 times the footprint length.

Xing et al. (2014b) considered the similarities of *Jialingpus* and *Asianopodus*. Both have a large, metatarsophalangeal region and widely divaricated digits. *Asianopodus* was first defined based on tracks from Japan (Matsukawa et al., 2005; Matsukawa, Lockley & Li, 2006) and is also distributed in Lower Cretaceous strata of Shandong and Gansu provinces and other regions within China (Xing et al., 2014d; Li et al., 2015; Lockley et al., 2015). *Jialingpus* differs from *Asianopodus* in size and the metatarsophalangeal structure, reflected in the size of the heel pad. Referring to the type material of the ichnospecies of *Asianopodus*, *A. pulvinicalx*, the tracks range from ca. 27.0–30.0 cm in length (Matsukawa et al., 2005), which is substantially larger than those of *Jialingpus* (approximately 10–20 cm in length). In addition, the “heel” pad is more robust and mostly undivided in *Asianopodus* if compared to *Jialingpus*, in which a small metatarsophalangeal portion behind digit II is visible (Xing et al., 2014b, 2014d). However, the different “heel” pad and its configuration could also be related to ontogenetic growth or vary with substrate-conditions. Moreover in some *Asianopodus*-like tracks (e.g. HYQ-F-1 and HYQ-F-12 in Fig. 7, HYQ-29 in Fig. 12) a smaller metatarsophalangeal pad behind digit II is visible as well. Furthermore some smaller *Jialingpus*-like tracks show a single large metatarsophalangeal area (e.g. HYQ-I-2, HYQ-D-2 in Fig. 5).

Li, Jiang & Wang (2020) described *Asianopodus niui* from the Lower Cretaceous Wuerhe area, Xinjiang. The preservation grades of *A. niui* are between 1 and 2 on the scale of (Belvedere & Farlow, 2016) creating significant differences in track morphology in the same trackway. Thus, we consider there are two reasons, that there is little justification for establishing a new ichnospecies. First, Belvedere & Farlow (2016) and Marchetti et al. (2019) argue against naming new ichnotaxa based on preservation grades of 2 or less. Second, *A. niui* does not present any uniquely diagnostic morphological features. Among the purportedly diagnostic information provided by Li, Jiang & Wang (2020), the Wuerhe specimens show larger footprint size (47.0–56.0 cm) and low mesaxony (0.34 from the holotype and 0.44 from paratype: Fig. 3 outline map). While, it is true that the tracks are large, it is somewhat puzzling that Li, Jiang & Wang (2020) assigned these tracks to *Asianopodus* because almost all *Asianopodus* tracks are much smaller with a maximum foot length of 32.0 cm (Fig. 16; Table 2). Conversely the tracks are more similar to *Chapus* (Li et al., 2006b) in size and other features such as digit III/footprint length: Table 2.

**Table 2:** Comparison of the type material of the monospecific ichnogenus Chapus and the three ichnospecies of *Asianopdus*.

Track #	L	W	L/W	M	Digit III/L	PL/SL	Source
*C. lockleyi* L1	58.2	42.6	1.36	0.53	0.62	–	Li, Bai & Wei, 2011; Lockley et al., 2018
*C. lockleyi* R1	50.0	39.0	1.28	0.45	0.59	–	Li, Bai & Wei, 2011; Lockley et al., 2018
*C. lockleyi* L2	53.0	40.0	1.32	0.45	0.59	–	Li, Bai & Wei, 2011; Lockley et al., 2018
*C. lockleyi* mean	53.7	40.5	1.32	0.48	0.60	126.6/233	Li, Bai & Wei, 2011; Lockley et al., 2018
*A. pulvinicalyx*	29.5	20.5	1.44	0.45	0.56	91.0/–	Matsukawa et al., 2005
*A. robustus*	32.0	26.0	1.23	0.40	0.55	–	Li, Bai & Wei, 2011; Lockley et al., 2018
*A. niui* holotype	56.0	42.0	1.33	0.64	–	172/332	Li, Jiang & Wang, 2020
*A. niui* paratype	47.0	31.0	1.52	0.52	0.62	–	Li, Jiang & Wang, 2020

**Note:**

(L), maximum length; (W), maximum width; (PL), pace length; (SL), stride length; (M), Mesaxony; L/W is dimensionless.

The *A. niui* holotype track, IVPP CV 26286.1, is the largest. Li, Jiang & Wang (2020) report a length/width ratio of 1.5, and a mesaxony value of 0.34. We consider the outline morphologies presented by these authors to be misleading. Thus, we have redrawn the outlines of this track (Fig. 16) and the paratype (IVPP CV 26286.2) and show that these values for IVPP CV 26286.1 appear to be questionable and should be 1.33 and 0.64 respectively: i.e., the holotype is less elongate but more strongly mesaxonic .

The description and documentation by Li, Jiang & Wang (2020: fig. 3) lacks the diagnostic 2-3-4 pad configuration and claw morphologies typical of theropods. This is partly due to the suboptimal preservation. We have herein added a re-interpretation of the holotype and paratype material (Fig. 16) and consider *A. niui* to be a *nomen dubium* based on lack of any clearly diagnostic features that distinguish it from other theropod track morphotypes. It should be stressed here that according the preservation guidelines of Belvedere & Farlow (2016) and Marchetti et al. (2019), increasingly encouraged by the ichnological community, the type material by which *A. niui* was introduced, is too poorly preserved and should not form the basis of a new ichnotaxon.

Most of the Huangyangquan tracks that fall into morphotype C tracks are likely to be poorly preserved tracks and extramorphological variations of morphotypes A and B. However, HYQ-E-11 is well-preserved and similar to track MGCM.H5 described by Xing et al. (2011). The latter is 13.4 cm in length, with a length/width ratio of 1.3 and mesaxony of 0.5. The metatarsophalangeal pads of these two tracks appear to be weaker than in morphotype A. Xing et al. (2011) assigned MGCM.H5 to *Kayentapus* isp., based on the V-shape metatarsophalangeal pads and wide divarication angle (70°). Compared with HYQ-E-11, the V-shape metatarsophalangeal pads are probably part of the metatarsal pad. *Kayentapus* is the ichnogenus name originally applied to relatively large (pes length ∼35 cm) tridactyl tracks of a bipedal theropod dinosaur first described from the Lower Jurassic Kayenta Formation of Arizona, by Welles (1971). The type ichnospecies *Kayentapus hopii* is more gracile than *Eubrontes* and is characterized by the absence of a hallux impression and the preservation of the metatarsophalangeal pad of digit IV well separated from the rest of the digit impressions (Welles, 1971; Lockley, Gierlinski & Lucas, 2011). However, these traits are not present in the Huangyangquan morphotype C. Due to the small quantity of well-preserved tracks, based on medium mesaxony and low length/width ratios, morphotype C is also similar to small-sized *Eubrontes* -like tracks (Olsen, Smith & McDonald, 1998; Lockley, 2009), however too poorly preserved to give a concrete assignment.

### Special preservation

***“3D” tracks and Scratch lines:*** HYQ-A-18 (Figs. 5, 17) preserves elongate metatarsophalangeal pads, at an angle to the track surface, and digit III with an outwards displacement drag mark, with width similar to digit II. It indicates that the trackmaker’s foot was impressed into the sediments at an angle, and then leaving a posterior mark, suggesting a wet sediment. A similar situation also occurs in YLSNHM01226, (Fig. 13) where the proximal end of digit II shows distinct scratch lines, and there are ~5 scratch lines within the well-preserved, 4.5 mm wide region, and the average line width is 0.8 mm. These scratch lines were probably made when individual pedal scales dragged through the sediment as the tracks were registered (Cobos et al., 2016). YLSNHM01246 is 13.7 cm long, (Fig. 14) located on nodules, and was described by Xing et al. (2011). The scale scratch lines of digit IV average 1.3 mm wide, and there are 6–7 lines per centimeter.

YLSNHM01265 is 25.2 cm in length (Fig. 17) with well-preserved scratch lines, which are mainly distributed in the outside of digits II and IV. The 10.3 cm long region of digit IV contains 22 well-preserved scratch lines, 0.7–1.7 mm in length (averaging 1.2 mm), and has ~8 scratch lines per centimeter. The 4.3 cm long region of digit IV also preserves scratch lines, 17 of which are 0.7–1.4 mm in length (averaging 1 mm), with ~8 scratch lines per centimeter. This indicates that the foot-scale sizes of the YLSNHM01265 trackmaker are relatively consistent, with an average of 1–1.2 mm in width. Theses scratch lines imply the direction of motion of the toes. They were impressed into the sediments at an almost vertical angle, then tucked and moved forward from the sediments. Distinct clues of toe tucking are visible between digit II and III of YLSNHM01265. The top surface of the track is 28 cm in length, obviously longer than the bottom (25.2 cm), which may be attributable to the backward motion of the foot. As Xing et al. (2011) listed, similar scale scratch lines are known from theropod tracks in the Lower Jurassic Moenave Formation, Utah (Milner, Lockley & Kirkland, 2006) and the Upper Cretaceous St. Mary River Formation, Alberta (Nadon, 1993), North Horn Formation, Utah (Difley & Ekdale, 2002), and Nemegt Formation, Mongolia (Currie, Badamgarav & Koppelhus, 2003). Similar scratch lines have been reported from other sites: e.g., the scratch lines associated with *Grallator* from the late Triassic of Colorado (Gaston et al., 2003, fig. 3) and Cretaceous tracks from Utah (Lockley, 2018). The larger the track, the fewer the scratch lines per centimeter, for example, the Mongolian tracks MPD 100F/12 is 68.9 cm long and has 5 to 6 scratch lines per centimeter (Currie, Badamgarav & Koppelhus, 2003).

HYQ-E-11 is 7.7 cm in length, (Fig. 10) without clear scratch lines, but shows distinct elements of the ‘anatomical track’. It shows the entry and exit positions of parts of the foot. Similar to YLSNHM01265, the toes were impressed into the sediments with a splayed status, the footprint was made with a divarication angle of 70°, then, the two lateral toes tucked, with divarication angle declining to 42°, and the foot left the sediments. The track may not represent the opening and tucking of the trackmaker’s toes, but it does display the movement of the trackmaker.

***Round claw impressions:*** YLSNHM01280 (Fig. 13) has an incomplete digit IV and well-preserved digits II and III. The round claw impressions at the distal end of digits II and III are discernable, because the trackmaker’s claws were impressed into the sediments at a wide angle and, when the trackmaker withdrew the pes from the substrate, sediments retracted and collapsed back. These round claw impressions are still the deepest parts of the track. Similar situations also occur in other theropod tracks (e.g. Wilson, Marsicano & Smith, 2009; Xing et al., 2014e).

***Wrinkle structures:*** Partial regions within tracks HYQ-K-1 and 2 (Figs. 8 and 18) preserve distinct wrinkle structures, especially in digits II and III of HYQ-K-1. Features of HYQ-K-1 and HYQ-K-2 are: (1) missing phalangeal pads; (2) wrinkles occurring only inside the deeper toes, being absent in the shallowest digit IV; (3) running in the same (namely longitudinal) direction as the toes. There are many possibilities explaining these wrinkle structures, such as the high degree of plasticity of the bedding surfaces, and strong continuity of the sediments, making it possible that the footprint can form an outline, but cannot leave details of phalangeal pads. Lockley et al. (2006a, p. 88) described Cretaceous ornithopod tracks with “longitudinal wrinkles sub-parallel to the axis of the digit impressions. Such wrinkles are not uncommon in tracks and under-tracks preserved in fine-grained sediments”. Likewise, Lockley (2018, p. 383) described a large ornithopod track cast where “The track walls show vertical striations caused during registration of the foot, and the underside of the cast shows many small ridges representing sand filled casts of cracks generated by registration of the foot.” Recently Falkingham, Turner & Gatesy (2020) explained wrinkle structure in footprints by the formation of penetrative tracks, where the foot leaves a series of imprints on successive layers by penetrating these with the digits.

### Avian theropod tracks

#### Description

**Figure 19:**
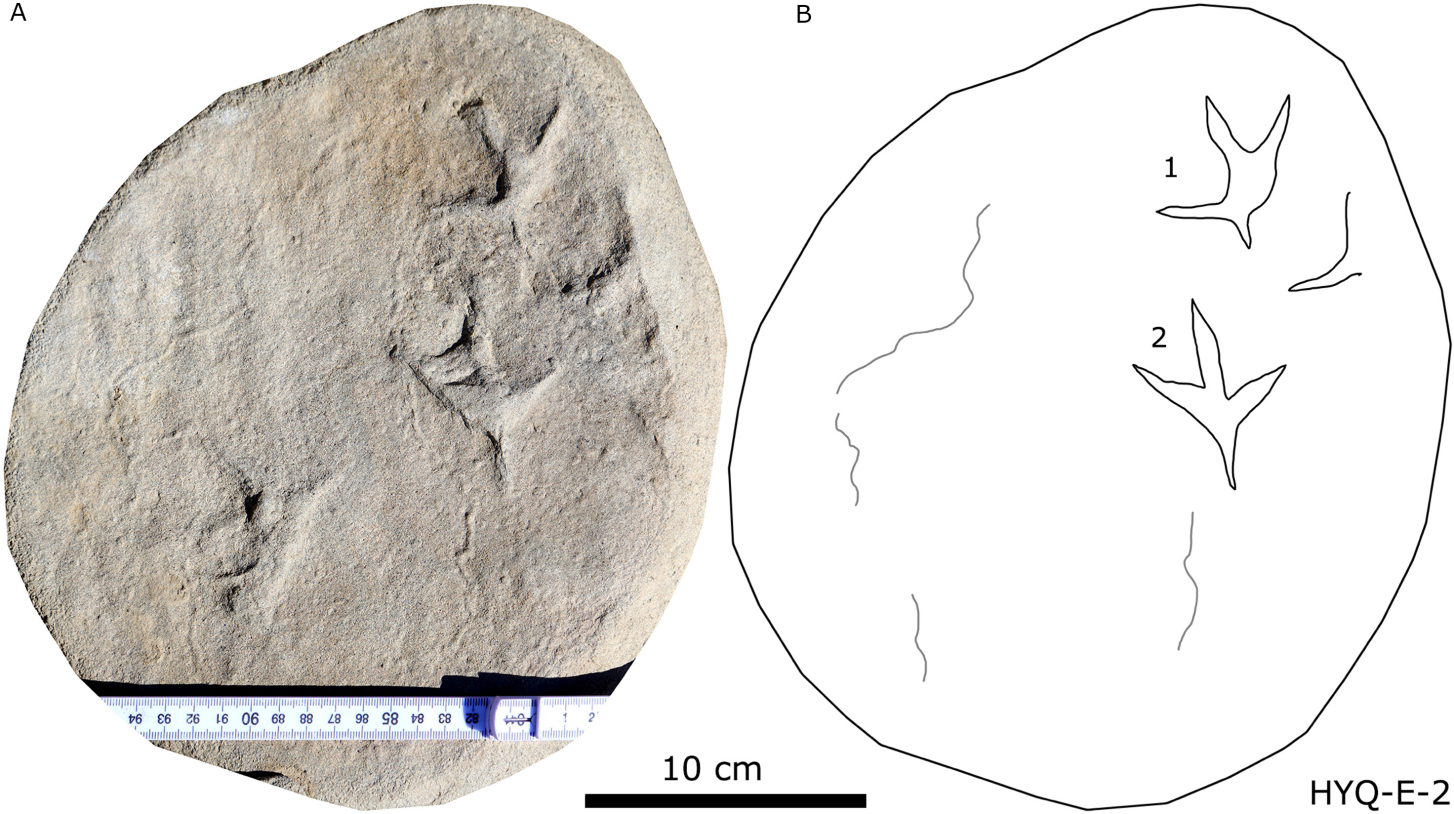
Photograph (A) and interpretive outline drawing (B) of bird tracks at Huangyangquan site E: HYQ-E-2.

**Figure 20:**
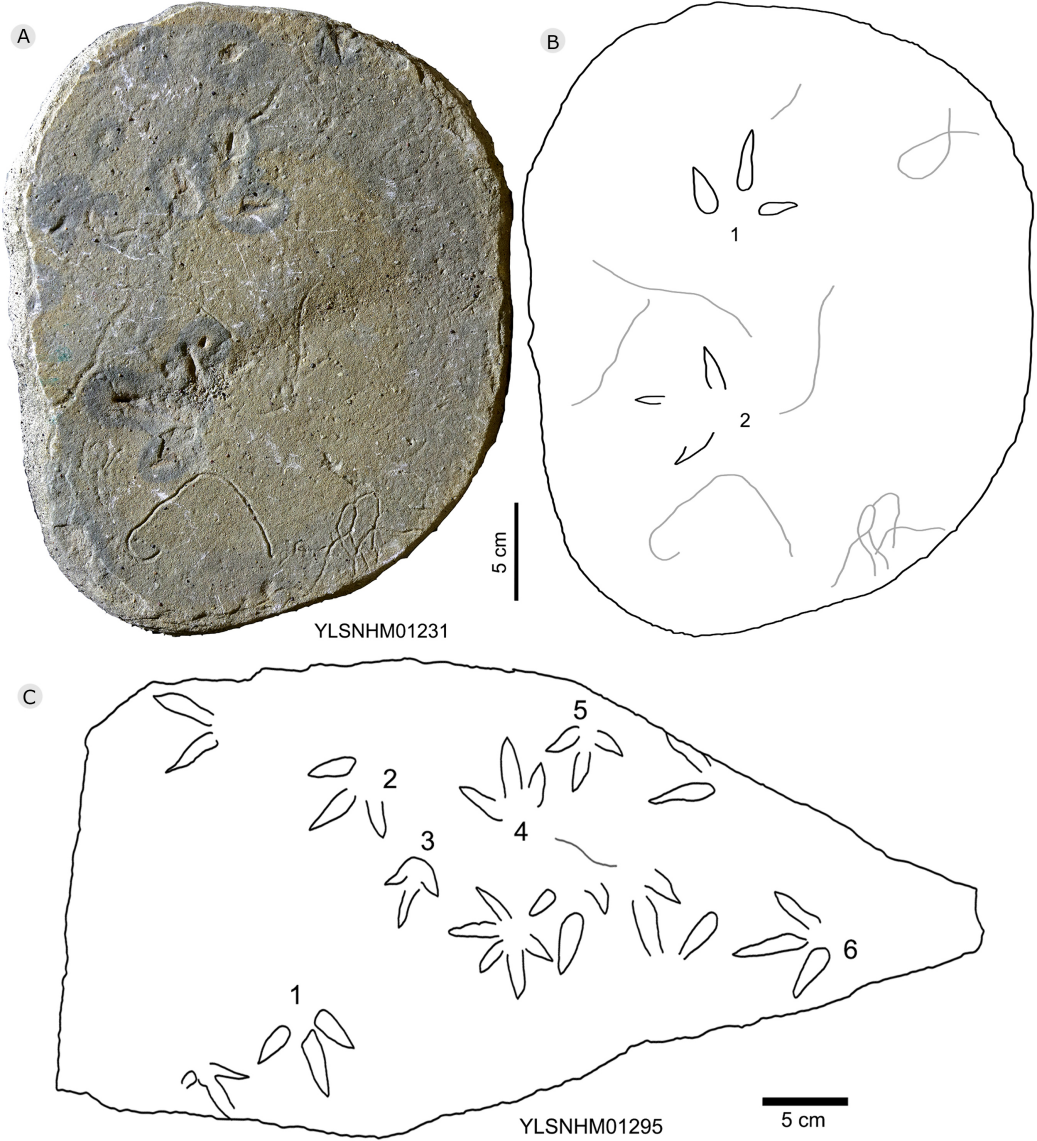
Photograph (A) and interpretive outline drawing (B) of bird tracks YLSNHM01231, and the photograph (C) of bird tracks YLSNHM 01295 at Huangyangquan sites.

**Figure 21:**
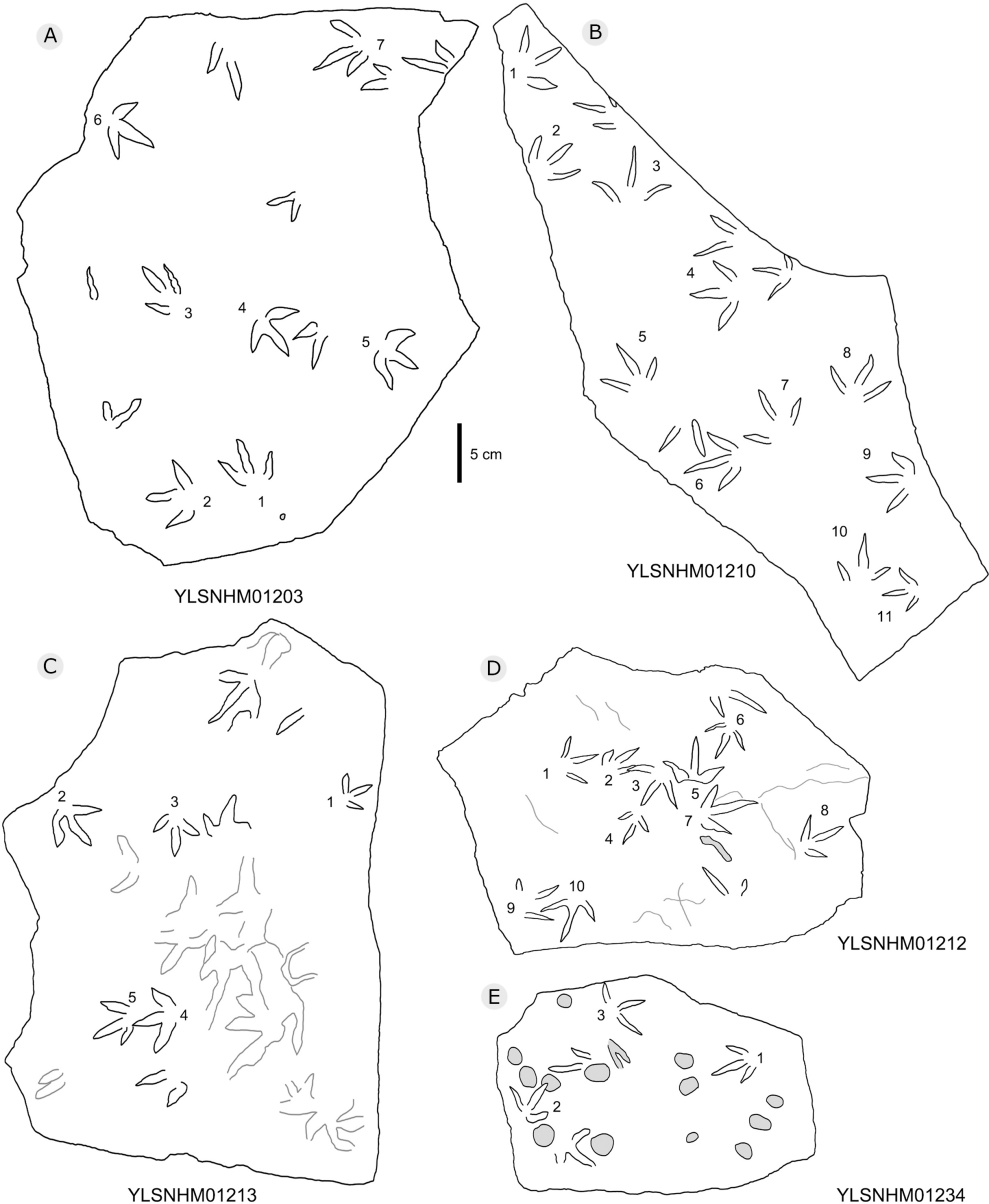
The interpretive outline drawing of bird tracks at Huangyangquan sites: YLSNHM01203 (A), 01210 (B), 01213 (C), 01212 (D), 01234 (E).

**Figure 22:**
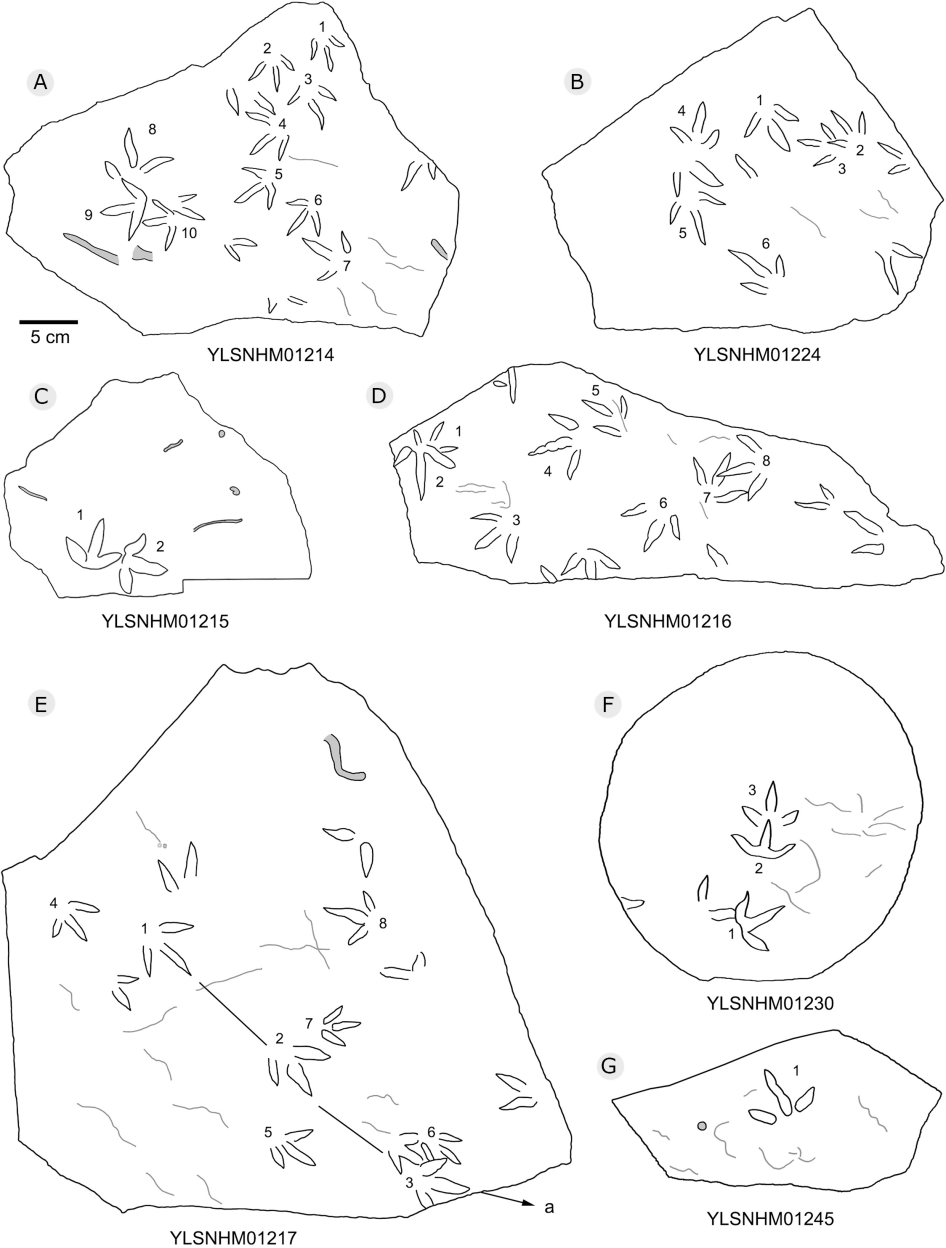
The interpretive outline drawing of bird tracks at Huangyangquan sites: YLSNHM01214 (A), 01224 (B), 01215 (C), 01216 (D), 01217 (E), 01230 (F), 01245 (G).

**Figure 23:**
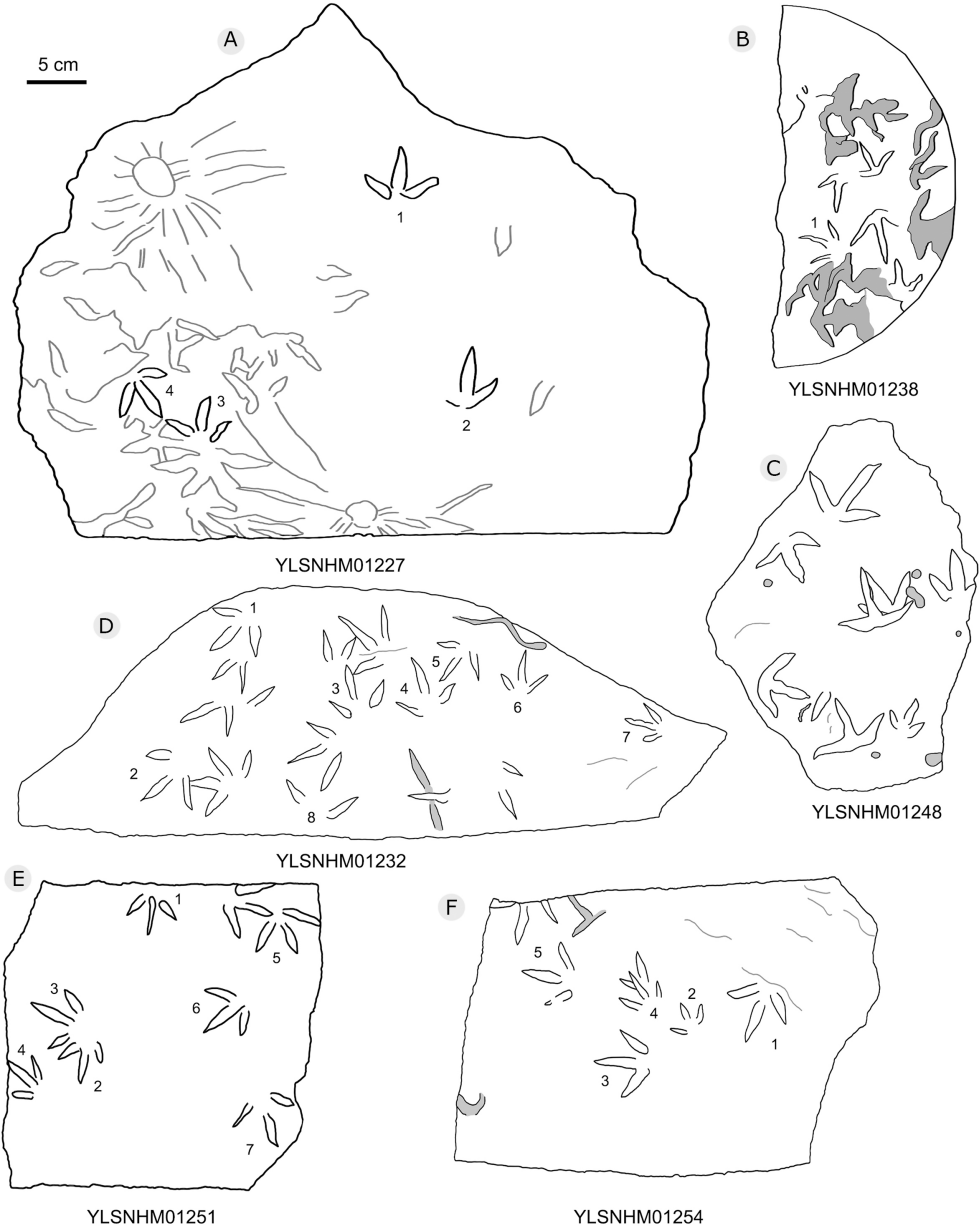
The interpretive outline drawing of bird tracks at Huangyangquan sites: YLSNHM01227 (A), 01238 (B), 01248 (C), 01232 (D), 01251 (E), 01254 (F).

**Figure 24:**
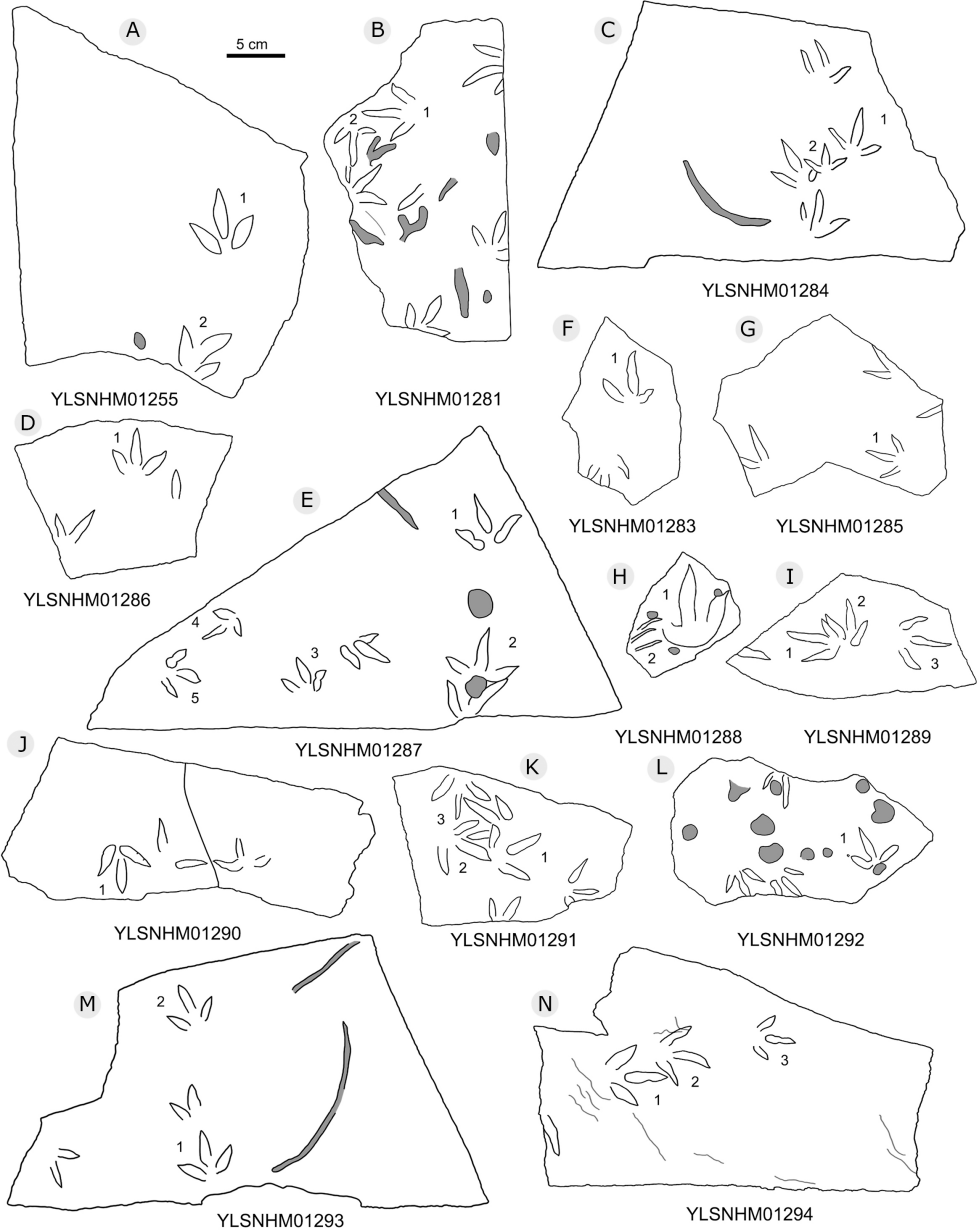
The interpretive outline drawing of bird tracks at Huangyangquan sites: YLSNHM01255 (A), 01281 (B), 01284 (C), 01286 (D), 01287 (E), 01283 (F), 01285 (G), 01288 (H), 01289 (I), 01290 (J), 01291 (K), 01292 (L), 01293 (M), 01294 (N).

**Figure 25:**
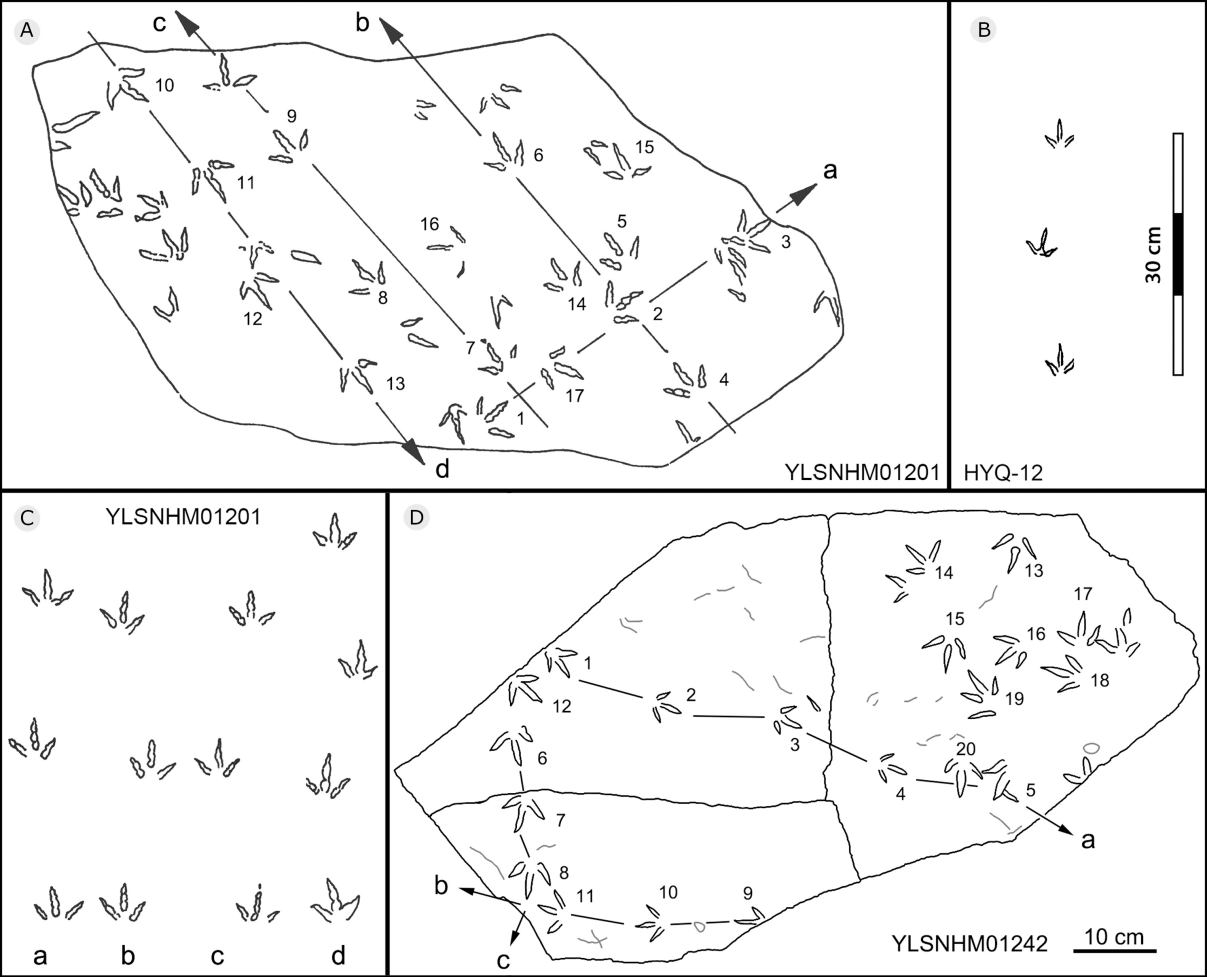
The interpretive outline drawing of bird tracks at Huangyangquan sites: YLSNHM01201 (A); HYQ-12 (B); YLSNHM01201 (C), 01242 (D).

**Figure 26:**
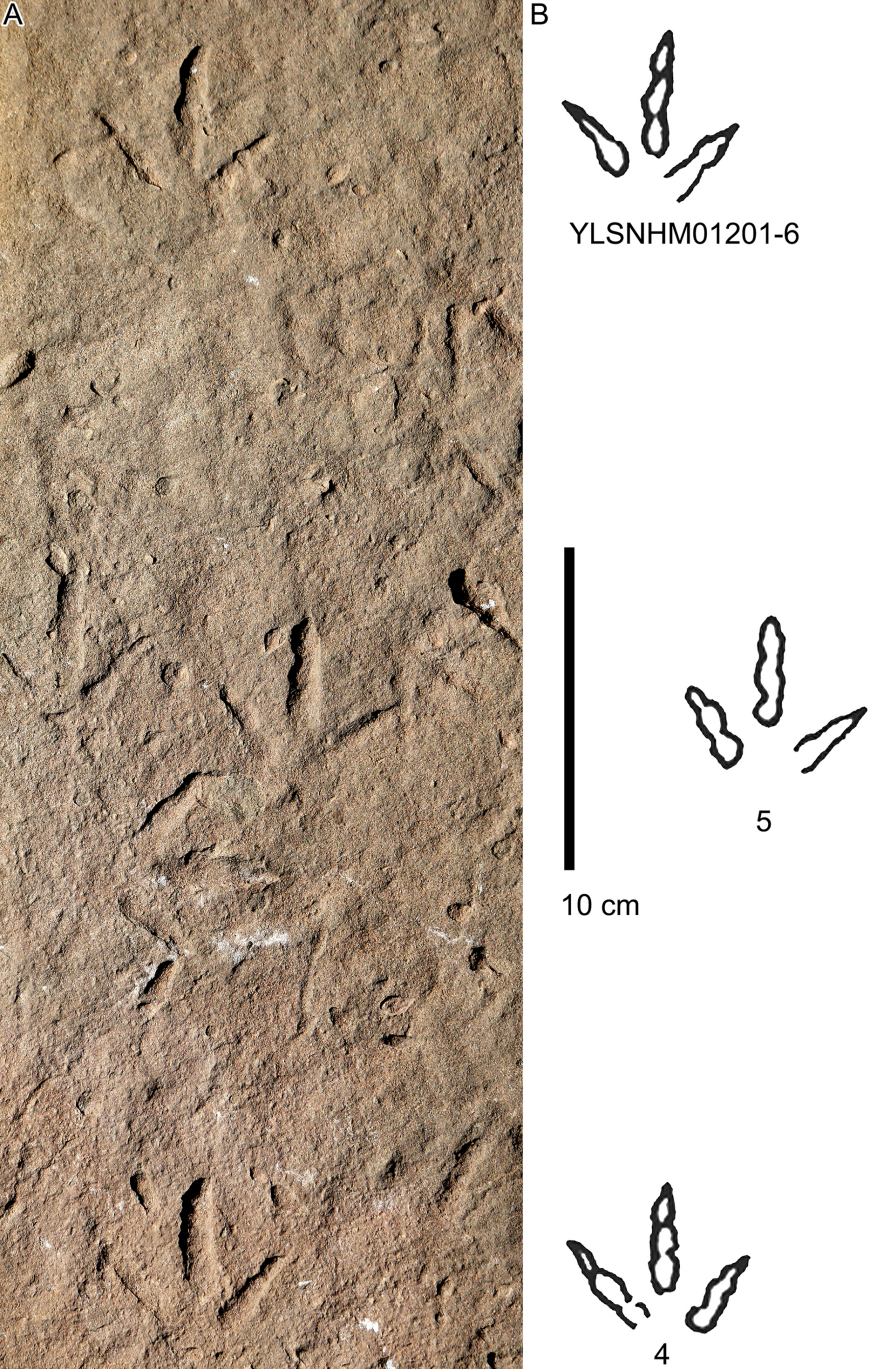
Photograph (A) and interpretive outline drawing (B)of bird tracks at Huangyangquan sites: YLSNHM01201-4–6.

**Figure 27:**
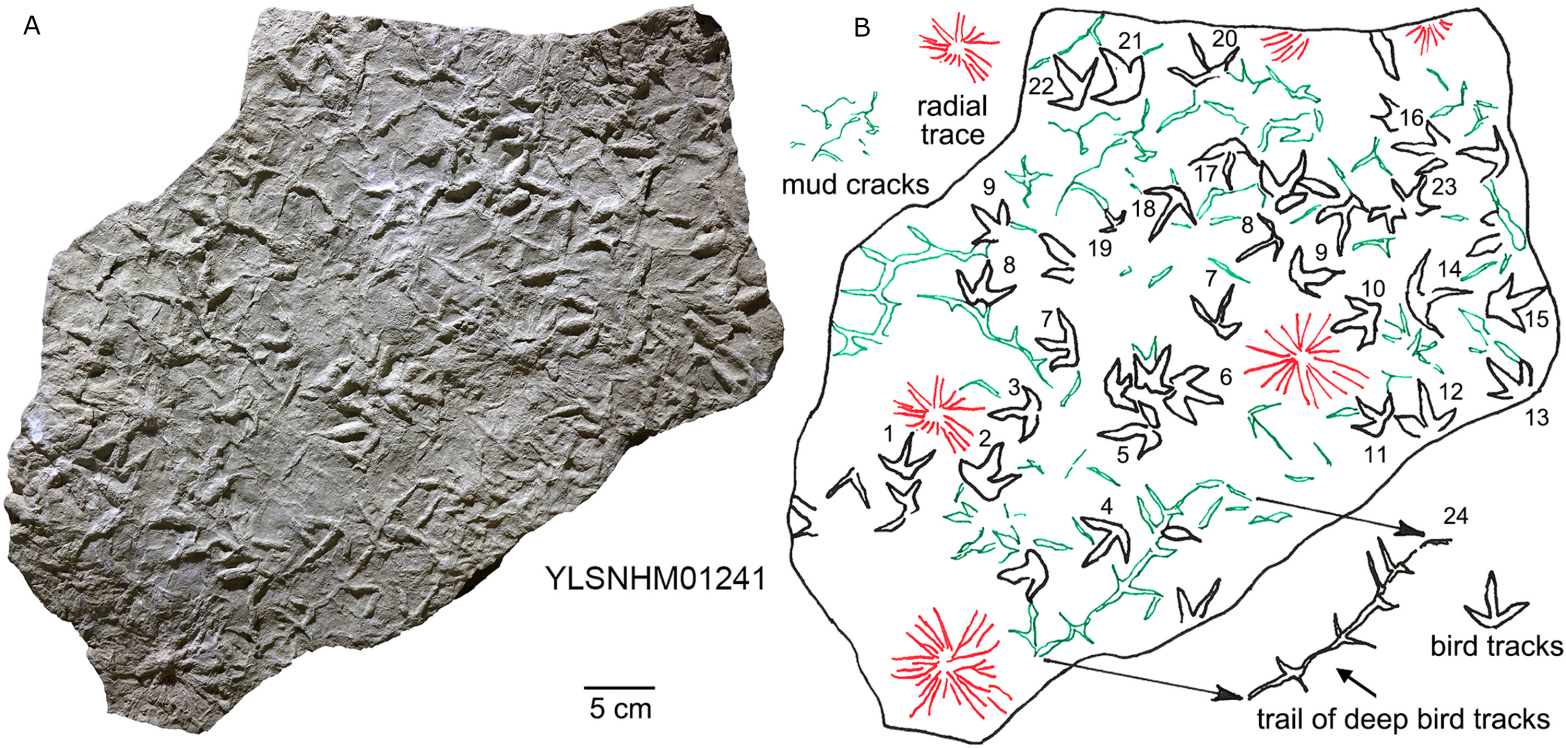
Photograph (A) and interpretive outline drawing (B) of bird tracks at Huangyangquan sites: YLSNHM01241.

**Figure 28:**
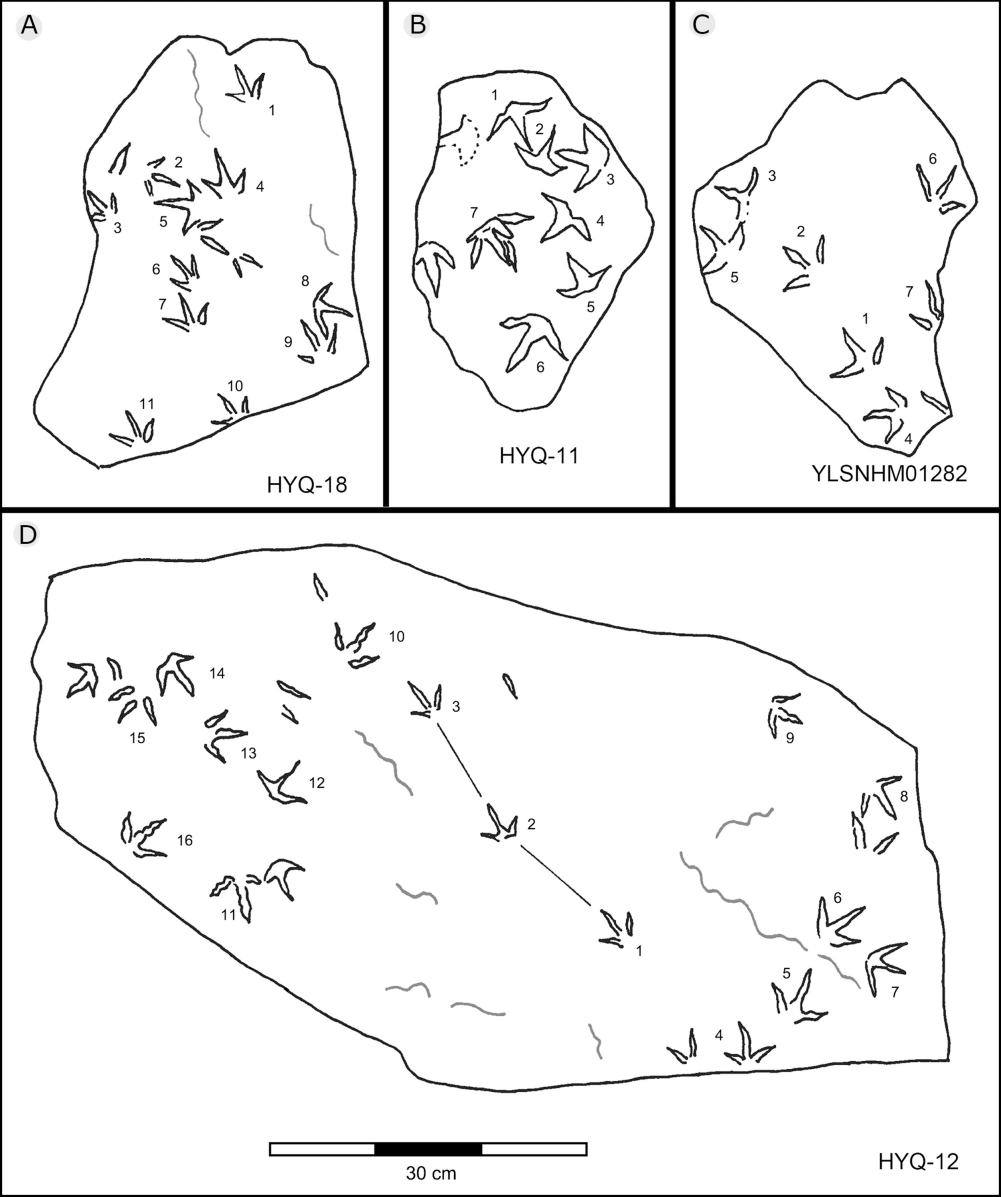
The interpretive outline drawing of bird tracks at Huangyangquan sites: HYQ-18 (A), 11 (B); YLSNHM01282 (C); HYQ-12 (D).

297 tracks from the Huangyangquan site were counted (see Table 1 for detailed list, and Supplement Information 2 for the measurements) (Figs. 2, 13, 14, 15, 19–28). The smallest track is 1.5 cm in length, and the largest is 6.4 cm in length. These tracks fall into seven size classes: <2.5 cm, one track; 2.5–3.0 cm, 11 tracks; 3.1–4.0 cm, 104 tracks; 4.1–4.4 cm, 56 tracks; 4.5–5.0 cm, 84 tracks; 5.1–5.9 cm, 38 tracks; >5.9 cm, 2 tracks. The mean length/width ratio is 0.9 (0.6–1.5). 240 tracks have a length/width ratio ranging from 0.6 to 1.0, 46 tracks range from 1.1 to 1.2, and 6 tracks have a ratio higher than 1.2. The mean mesaxony is 0.47, focussing on the range of 0.30–0.60 (261 tracks). Based on these measurements and other morphological traits, the tracks can be divided into two different morphotypes.

Morphotype A contains only four tracks, HYQ-E-2-1–2 (Fig. 19), and HYQ-E-3-1 and 4 (Fig. 15). These specimens are medium-sized, sub-symmetric, functionally tetradactyl bird tracks with three widely splayed digits (II–IV) and a posteriorly directed hallux. HYQ-E-2-2 is the best preserved and is 4.6 cm long with a length/width ratio of 0.9 and mesaxony of 0.43. The hallux is shallow when compared to the depths of digits II–IV. Digit III is the longest digit, slightly longer than digit IV, and substantially longer than digit II. Digit II is slightly longer than the hallux. The average percentages of lengths of hallux as compared to digits II and III are 91% and 68% (52% and 85% in HYQ-E-2-1, 67% and 65% in HYQ-E-3-1). Subtle web traces are present and confined to the proximal hypex between digits II and III. Digital pad impressions are absent. The divarication angles between digits II and III (59°) are larger than those between digits III and IV (42°).

All other bird tracks are assigned to morphotype B, which is best represented by YLSNHM01201(Figs. 25 and 26) trackway b, consisting of the tracks YLSNHM01201-4–6. These tracks lack hallux impressions. Their average length is 5.0 cm, length/width ratio is 0.9, and mesaxony is 0.42. Digit III is the longest digit. Digits II and IV are subequal in length. Digit II is broader than digits III and IV. Digits II and III are deeper than digit IV. Digits II and III have two and three pads, respectively, and the pads of Digit IV are unidentifiable. The divarication angles between digits II and III are smaller than those between digits III and IV. The average divarication angle between digits II and IV is 92° (range 89°–99°). The pace angulation is 160°. Tracks in the trackway exhibit slight positive (inward) rotation.

Besides trackway b, YLSNHM01201 also preserves trackways a, c, and d, which consist of 3, 3 and 4 tracks, respectively. Trackways b and c are parallel trackways and that share the same orientation. Trackway d is also parallel to trackway b, but with an opposite orientation. Trackway a is perpendicular to the other three trackways. Parallel trackways may indicate that the trackmakers were traveling as part of a social group. The ratio of the pace length to the track length of trackways a–d is 4.0, 3.6, 4.0, and 2.9, and the ratio of trackway YLSNHM01242-1–5 (Fig. 25) is 3.8. This ratio is 1.8 in YLSNHM01242-6–8, 3.4 in HYQ-12-1-1–3, and 1.9 in HYQ-J-9-4–5. Overall, for morphotype B trackways, these ratios mainly concentrate on the range of 2–4, representing different speeds.

Other Huangyangquan morphotype B tracks are similar to YLSNHM01201 trackway b. However, the digital pad impressions of most tracks are absent. HYQ-11-6 is the largest track, the length is 6.4 cm, and the length/width ratio is 0.9, representing the largest trackmaker in this region.

Tracks such as HYQ-E-1 and YLSNHM01231 (Figs. 15 and 20 respectively) all have a circle gray concentric trace, the color differences may be caused by water permeating along the beddings.

Most tracks display three widely divergent digit traces, but within some deeper tracks (such as YLSNHM01248: Fig. 23), the proximal ends of the three digits are joined and form a smoothly curved metatarso-phalangeal region.

### Comparison & discussion

Xing et al. (2011) described avian theropod footprints at the Huangyangquan site and classified them as *Koreanaornis dodsoni*, *Goseongornipes* isp., *Aquatilavipes* isp., and *Moguiornipes robusta*. These shorebird-morphotype tracks are the first solid evidence of avian theropods in the Tugulu Group. Xing et al. (2011) suggested that *Moguiornipes* tracks may represent avian theropods with lobed feet. However, such wide digit traces could also be the product of the widening of digit traces by dissolution of traces that were originally narrower. Similar preservation is seen in tracks from the Cretaceous Jingchuan Formation of the Chabu Region in Inner Mongolia: compare tracks illustrated by Li et al. (2009: fig. 10A–10C with fig. 10D, plates 1 and 2) and, Lockley, Matsukawa & Li (2012: figs. 3 and 4), with *Moguiornipes*. Some of these Chabu tracks were illustrated and labelled as *Aquatilavipes* by Li, Bai & Wei (2011) but later, on the basis of more abundant material some of the illustrated tracks were formally named *Tatarornipes* (Lockley, Matsukawa & Li, 2012) and distinctions were drawn between tracks with slender toe traces that more accurately reflected foot morphology and wider toed forms that reflected preservational effects. Thus we alert ichnologists to the possibility that *Moguiornipes* is a taphotaxon (sensu Lucas, 2001b) that represents a preservational variant of *Aquatilavipes* or *Tatarornipes*, evidently associated with sandy less cohesive substrates than those on which slender-toed tracks are registered. This conclusion is reinforced by the multivariate analysis of Buckley, McCrea & Lockley (2016) who found no statistical difference between *Moguiornipes robusta* and *Koreanaornis dodsoni*. As noted below, the latter ichnospecies is better accommodated in *Aquatilavipes* as *A. dodsoni* comb. nov.

Koreanornipodidae includes the small subsymmetric functionally-tridactyl tracks *Koreanaornis* (Kim, 1969; Lockley et al., 1992) and *Pullornipes* (Lockley et al., 2006b). The latter differs from *Koreanaornis* mostly in having a small postero-medially directed hallux, which is absent or very rare and inconspicuous in *Koreanaornis*. Features of *Koreanaornis dodsoni* that distinguish it from *K. hamanensis* include: smaller divarication angles between digits II and IV, greater overall track length and width, persistent absence of digit I, and absence of digital pad impressions. Compared with the original type *Koreanaornis hamanensis*, *K. dodsoni* is larger, with a reported, more variable size range of 3.0–6.3 cm (Xing et al., 2011). The larger measurement is markedly inconsistent with the identification as *Koreanaornis*. Likewise the *K. dodsoni* holotype (length 4.4 cm, width 5.7 cm) is more typical of *Aquatilavipes*, (ichnofamily Avipedidae according to Sarjeant & Langston, 1994) and raises the question of whether *K. dodsoni* would be better accommodated in *Aquatilavipes dodsoni* as a new combination. However, because Cretaceous shorebird tracks need a comprehensive ichnotaxonomic revision, we avoid a concrete assignment.

*Aquatilavipes* (Currie, 1981) is similar to *Koreanaornis*. It is important to note the size distinction between small type *Koreanaornis* and larger type *Aquatilavipes* because among modern shorebirds such size differences clearly represent different trackmaking species. Even though size is not a criterion for differentiation of otherwise identical ichnites (ichnotaxa), the distinction between *Koreanaornis* and *Aquatilavipes*, two of the first three avian ichnotaxa named from the Mesozoic, is well established in the ichnological literature (Kim, 1969; Currie, 1981; Lockley et al., 1992; Lockley & Harris, 2010). It is also relevant to note that two small ichnospecies in the avian ichnogenus *Avipeda* are very similar to *Koreanaornis* (Lockley & Harris, 2010; Lockley et al., 2020). According to Sarjeant & Langston (1994) *Avipeda* is the basis for the amended ichnofamilial concept Avipedidae which includes *Aquatilavipes*. Moreover, multivariate analysis clearly separates Koreanapodidae (mostly *Koreanaornis*) and Avipedidae (including *Aquatilavipes*) (Buckley, McCrea & Lockley, 2016).

The first avian theropod tracks reported from China were named *Aquatilavipes* as at the time most ichnologists, including the Chinese paleontologists working with the Canadian paleontologists (Zhen et al., 1987, 1995 following Currie, 1981) were not aware of the original, and somewhat obscure report of *Koreanaornis* (Kim, 1969). Therefore, various “*Aquatilavipes*” from China were subsequently reclassified as *Koreanaornis*, such as *Koreanaornis anhuiensis* (Jin & Yan, 1994; Lockley et al., 2013; Xing et al., 2018b). *Aquatilavipes swiboldae*, the type ichnospecies, was originally erected by Currie (1981) from the Lower Cretaceous of Canada to describe tracks of about 4–5 cm width and length. The main difference between *Koreanaornis* and *Aquatilavipes* is size, especially length which is the least variable parameter: *Koreanaornis* (width ranges from 2.5–4.4 cm) is smaller than *Aquatilavipes* (4–5 cm wide and long), and *Koreanaornis* occasionally displays a small hallux trace (Lockley et al., 1992; Anfinson et al., 2009). Xing et al. (2011) described *Aquatilavipes* isp. based on twenty-two complete natural casts on two slabs, and considered that these tracks differ from *Koreanaornis dodsoni* in size and in having low, flat metatarsophalangeal portions and slender digits. *Aquatilavipes* has been reported from Gansu (Zhang et al., 2006) and represents tracks too big to be classified as *Koreanaornis*.

In contrast to the ichnogenera *Koreanaornis* and *Aquatilavipes*, the latter representing the ichnofamily Avipedidae, *Ignotornis* and *Goseongornipes*, (ichnofamily Ignotornidae) are morphologically quite distinct (Mehl, 1931; Lockley et al., 2006b, 2009; Lockley & Harris, 2010). In contrast to *Ignotornis* and *Goseongornis*, *Koreanaornis* and *Aquatilavipes* lack a well-developed hallux (often lack a hallux at all) as well as interdigital webbing traces. *Ignotornis* and *Goseongornis* are slightly semipalmate tetradactyl bird tracks with three widely splayed digits (II–IV) and a comparatively well-developed posteriorly directed hallux. *Ignotornis*, however is larger than *Goseongornipes* with a more pronounced digit III-IV web confined to the proximal hypex between digits III and IV (Lockley et al., 2006a).

Previously Xing et al. (2011) assigned one trackway, YLSNHM01282 (Fig. 28), to *Goseongornipes* isp., based on two tracks with a small, caudally directed hallux. However, new observations show that the “thumb” of YLSNHM01282-2 and 5 may actually be an invertebrate trace: thus, the photo and interpretive outline drawing from Xing et al. (2011: fig. 5) has been re-illustrated correctly (Fig. 28). Thus, these specimens lack confirmed hallux traces. However, despite containing only four tracks, the newly recorded morphotype A

(HYQ-E-2, HYQ-E-3-1 and 4) shows clear posteriorly directed hallux impressions. The best example of an unequivocal avian trackway with hallux traces from the Huangyangquan assemblages is HYQ-E-2 which clearly shows all the characteristics of *Ignotornis* (Mehl, 1931; Lockley et al., 2009).

The Ignotornidae had a global distribution during the Cretaceous (Aptian–Cenomanian), with most reports confined to North America (Lockley & Harris, 2010), and localities in South Korea (Kim et al., 2006; Lockley, Matsukawa & Li, 2012). The Huangyangquan specimen HYQ-E-2 appears to be the first example of the Ichnogenus *Ignotornis* (ichnofamily Ignotornidae) reported from China. However, the related ichnogenus *Goseongornipes* isp. also represents the ichnofamily Ignotornidae (Kim et al., 2006; Lockley et al., 2006b). Thus although Xing et al. (2011) reported *Goseongornipes* isp. from the Huangyangquan site and this ichnogenus level idenitification is in doubt, we nevertheless confirm the presence of a few related ignotornid tracks including unequivocal *Ignotornis*. We note also that Xing et al. (2018a) documented *Goseongornipes* isp. from the Lower Cretaceous of Donghai County, Jiangsu Province, China. The new records of *Ignotornis* isp. from Huangyangquan extend the geographic and stratigraphic distribution of Ignotornidae.

### Stegosaur tracks

**Figure 29:**
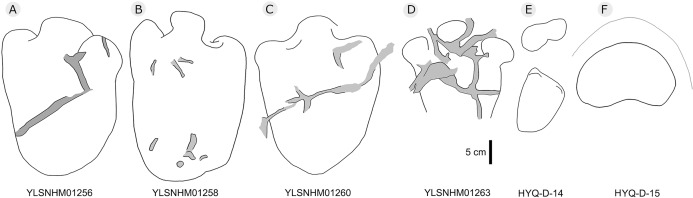
The interpretive outline drawing of stegosaur tracks at Huangyangquan sites: YLSNHM01256 (A), 01258 (B), 01260 (C), 01263 (D); HYQ-D-14 (E), 15 (F).

Xing et al. (2013d) described many thyreophoran tracks from Wuerhe area, which have been assigned to the stegosaur ichnotaxon *Deltapodus curriei* and are considered likely to be the tracks of *Wuerhosaurus homheni*. During new exploration, the main authors discovered 17 isolated pes traces (HYQ-B1p–16p) and one manus trace (HYQ-B17m) at site B, including some tracks representing a juvenile (Xing et al., 2021). Specimens in the YLSNHM collections (YLSNHM01256, 01258, 01260 and 01263), HYQ-32m, and specimens from site D (HYQ-D-14m and 14p, D-15m) will be described herein (see Table 1 for detailed list, and Supplement Information 2 for the measurements) (Fig. 29).

HYQ-D-14p and HYQ-D-14m are a set of manus and pes prints, with lengths of 5.4 and 12.7 cm and length/width ratios of 0.5 and 0.7, respectively. There is a small indentation in the posterior of the metacarpophalangeal region of HYQ-D-14m. The digit marks are ambiguous, however, the area ratio of the manus to pes can be calculated (1:2.3), the same value as for the type specimens (Xing et al., 2013d).

The lengths of the manus prints HYQ-D-15m (Fig. 29) and HYQ-32m (Fig. 12) is 14.5 and 19.8 cm, with the same length/width ratio of 0.7. These prints are entaxonic and oval-shaped, although somewhat irregular, and with indistinct digit marks from digit I–V.

The average length of the pes prints YLSNHM01256, 01258, 01260 is 33.1 cm (31.6 cm–34.7cm), with a length: width ratio of 1.7 (1.5–2.0). All tracks have three digits, the heel region is elongate and has smoothly curved margins. The best-preserved is YLSNHM01263. Though it only preserves the distal end, it exhibits three digits, each forming distinct, short, rounded impressions. Based on the trackway described by Xing et al. (2013d), the most robust, longest and deepest of the two outer digits is digit II. The medial digit III is almost as wide and long as digit IV. These tracks have weak mesaxony (anterior triangle length / width ratio), averaging 0.21 (0.20–0.23, *n* = 4).

Overall, the newly recorded large and small thyreophoran tracks from Huangyangquan site are comparable with *Deltapodus curriei* (Xing et al., 2013d). Though some poorly-preserved manus and pes prints show features of sauropod tracks (Lockley et al., 2002), the area ratio is comparable with that of *D. curriei*, indicating that sauropod tracks have not yet been recorded in the region.

### Pterosaur tracks

#### Description

**Figure 30:**
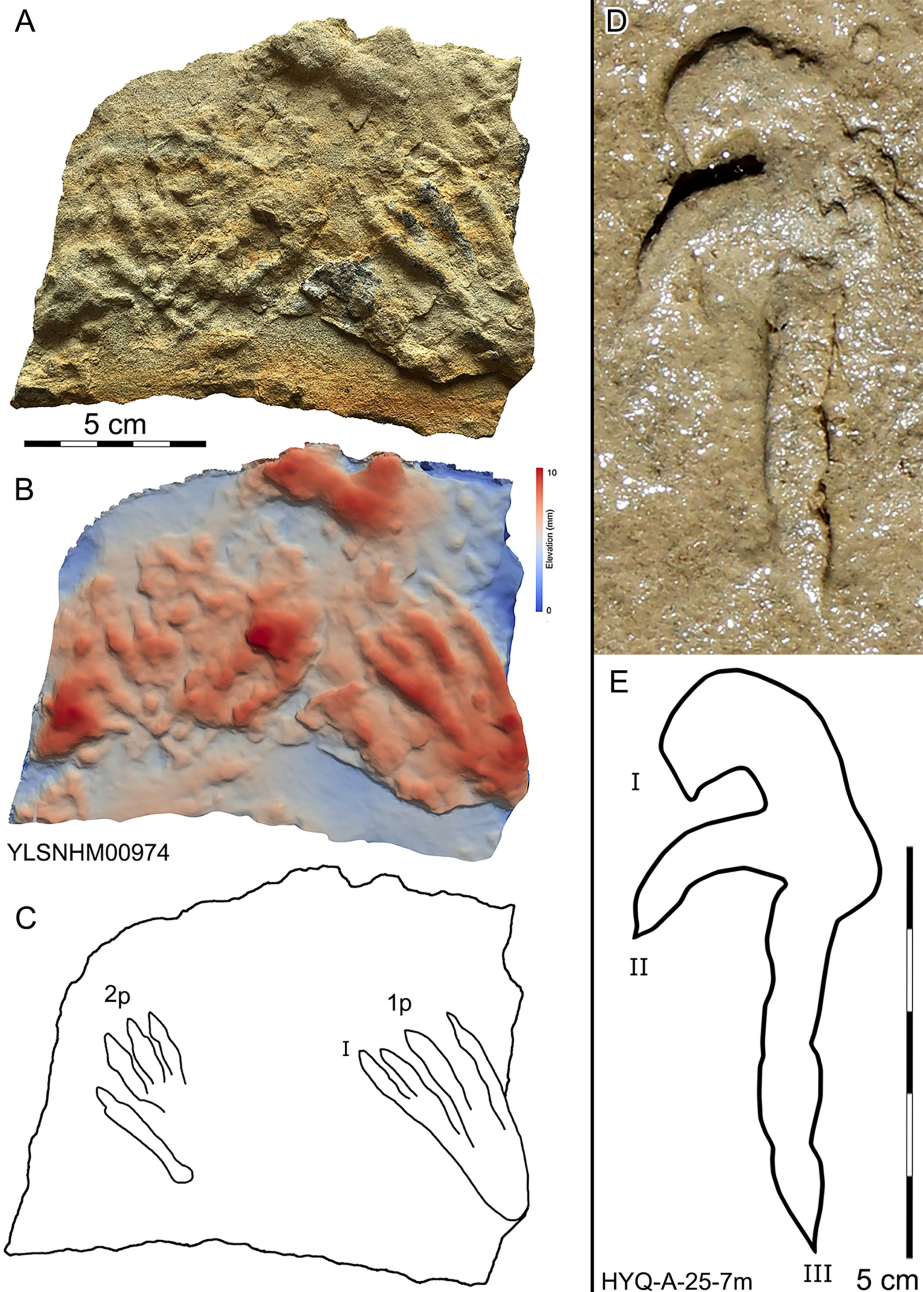
Photograph (A, D), elevation image (B) and interpretive outline drawings (C, E) of pterosaur tracks at Huangyangquan sites: YLSNHM00974 and HYQ-A-25-7m.

**Figure 31:**
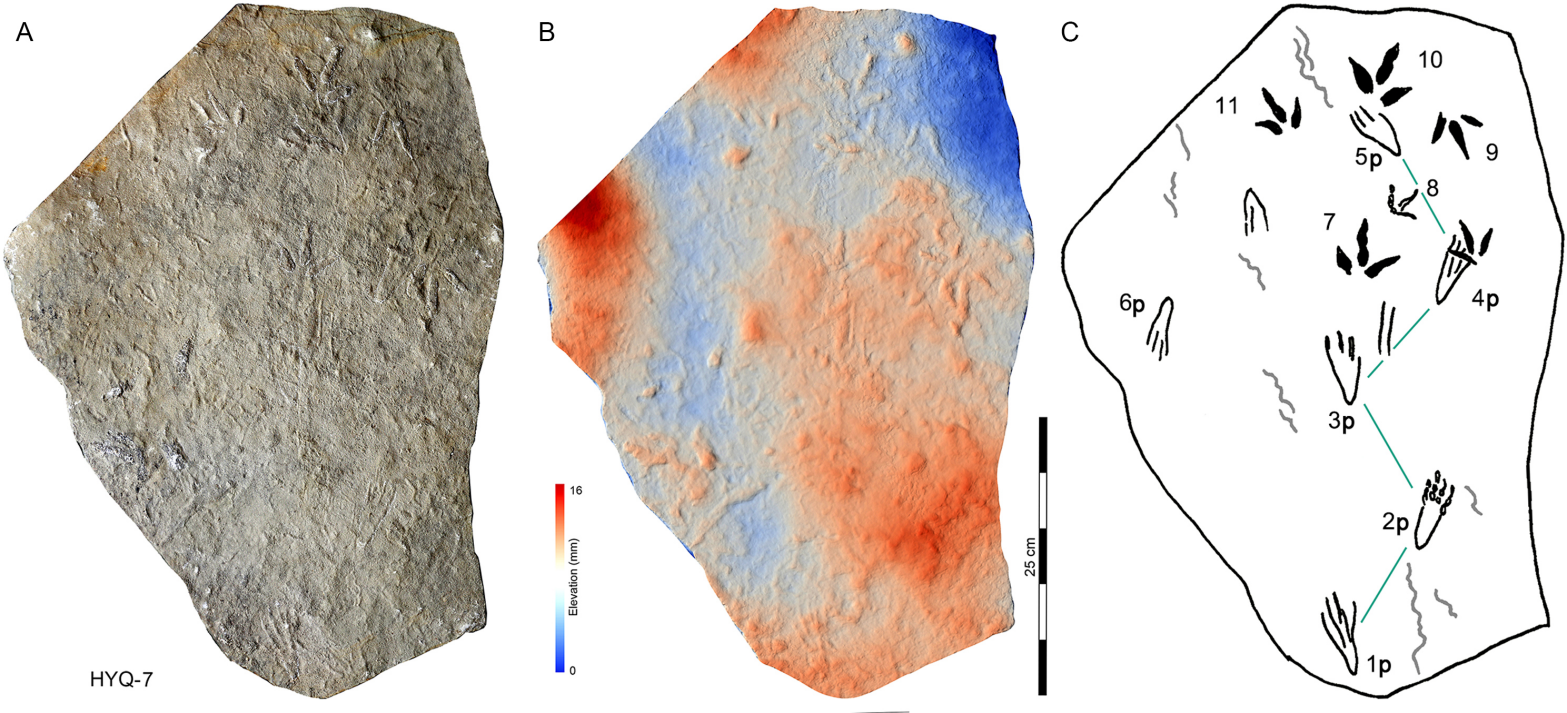
Photograph (A), elevation image (B) and interpretive outline drawing (C) of pterosaur tracks HYQ-7 at Huangyangquan sites.

**Figure 32:**
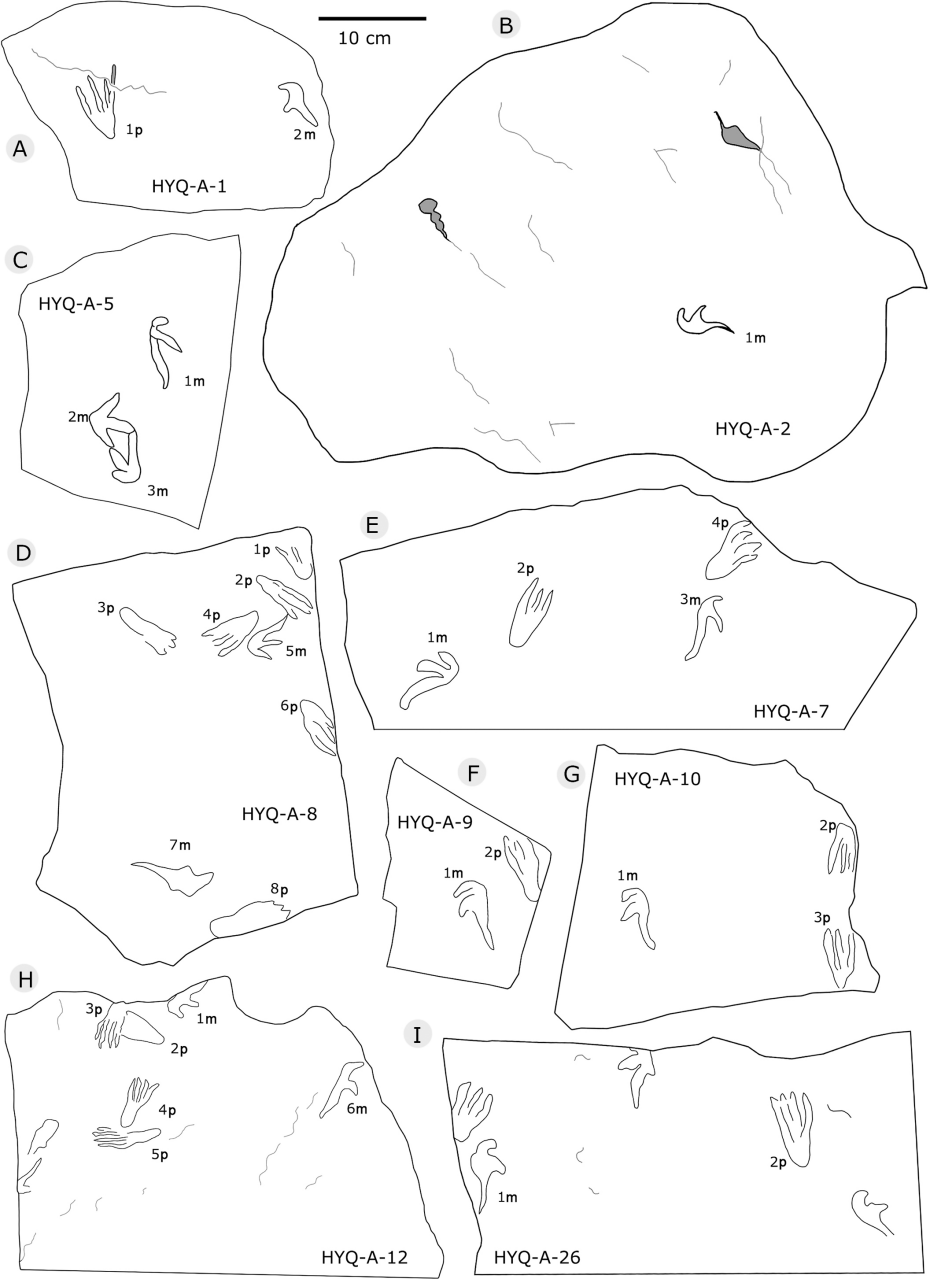
The interpretive outline drawing of pterosaur tracks at Huangyangquan sites: HYQ-A-1 (A), 2 (B), 5 (C), 8 (D), 7 (E), 9 (F), 10 (G), 12 (H), 26 (I).

**Figure 33:**
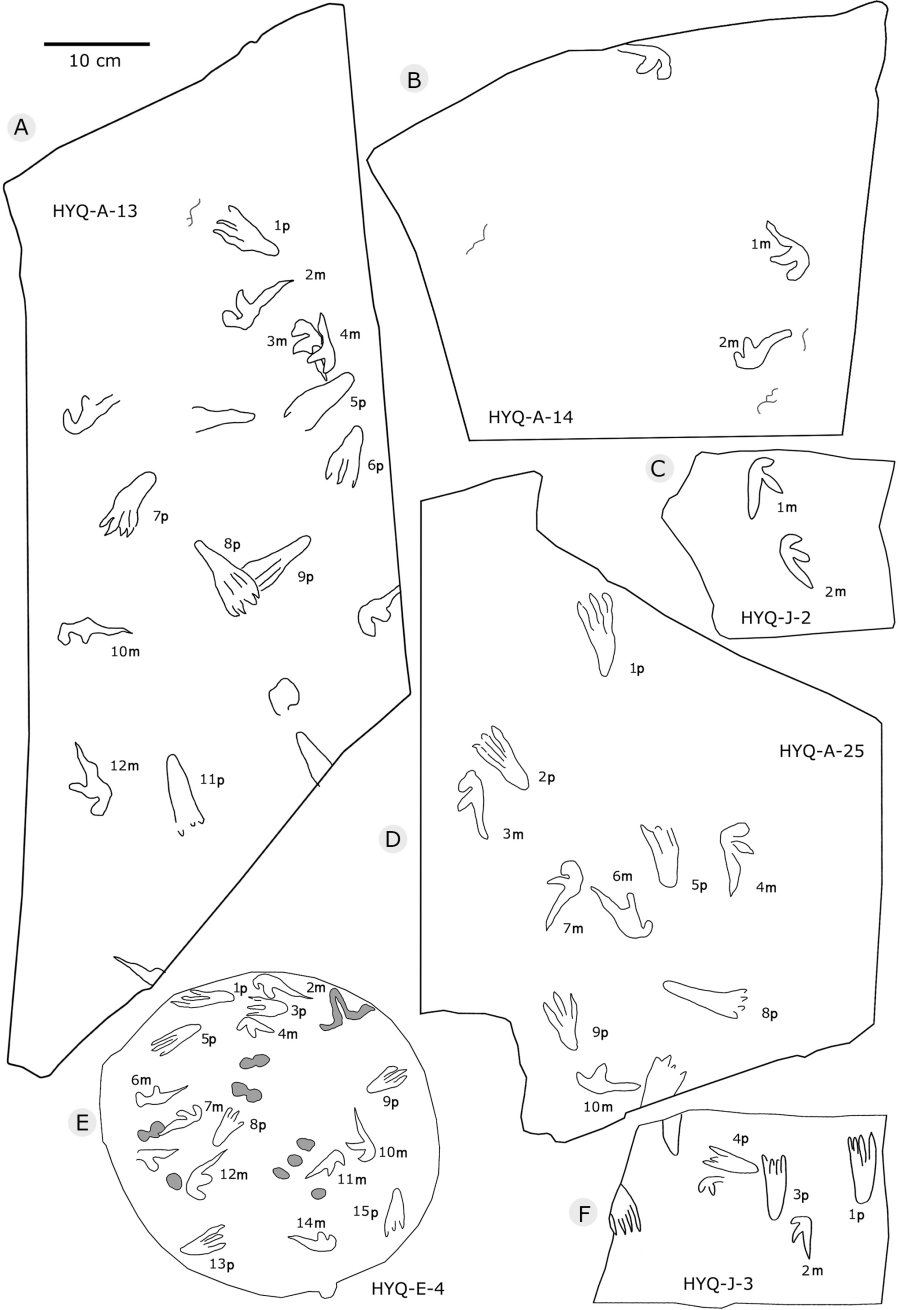
The interpretive outline drawing of pterosaur tracks at Huangyangquan sites: HYQ-A-13 (A), 14 (B); HYQ-J-2 (C); HYQ-A-25 (D); HYQ-E-4 (E); HYQ-J-3 (F).

**Figure 34:**
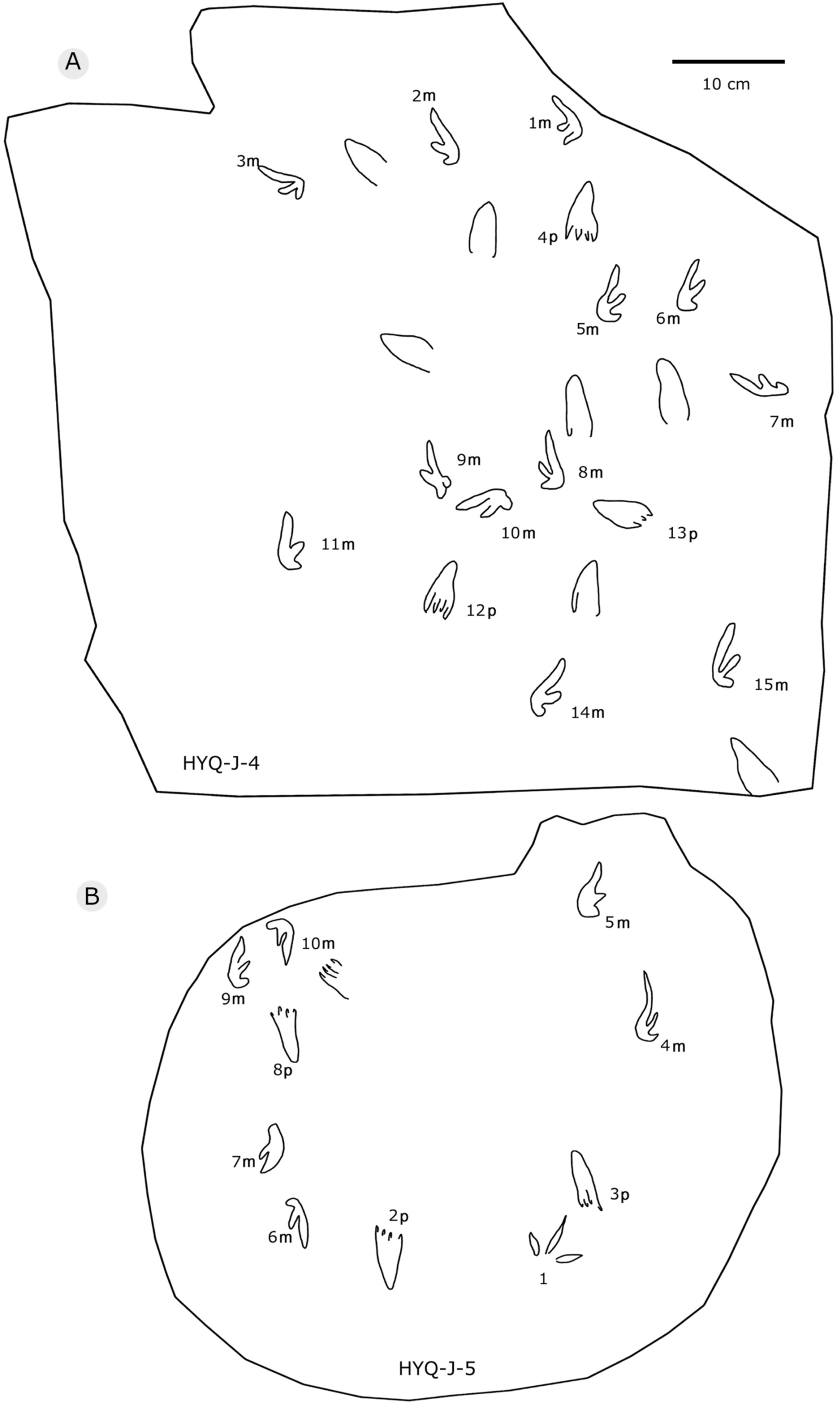
The interpretive outline drawing of pterosaur tracks at Huangyangquan sites: HYQ-J-4 (A), 5 (B).

**Figure 35:**
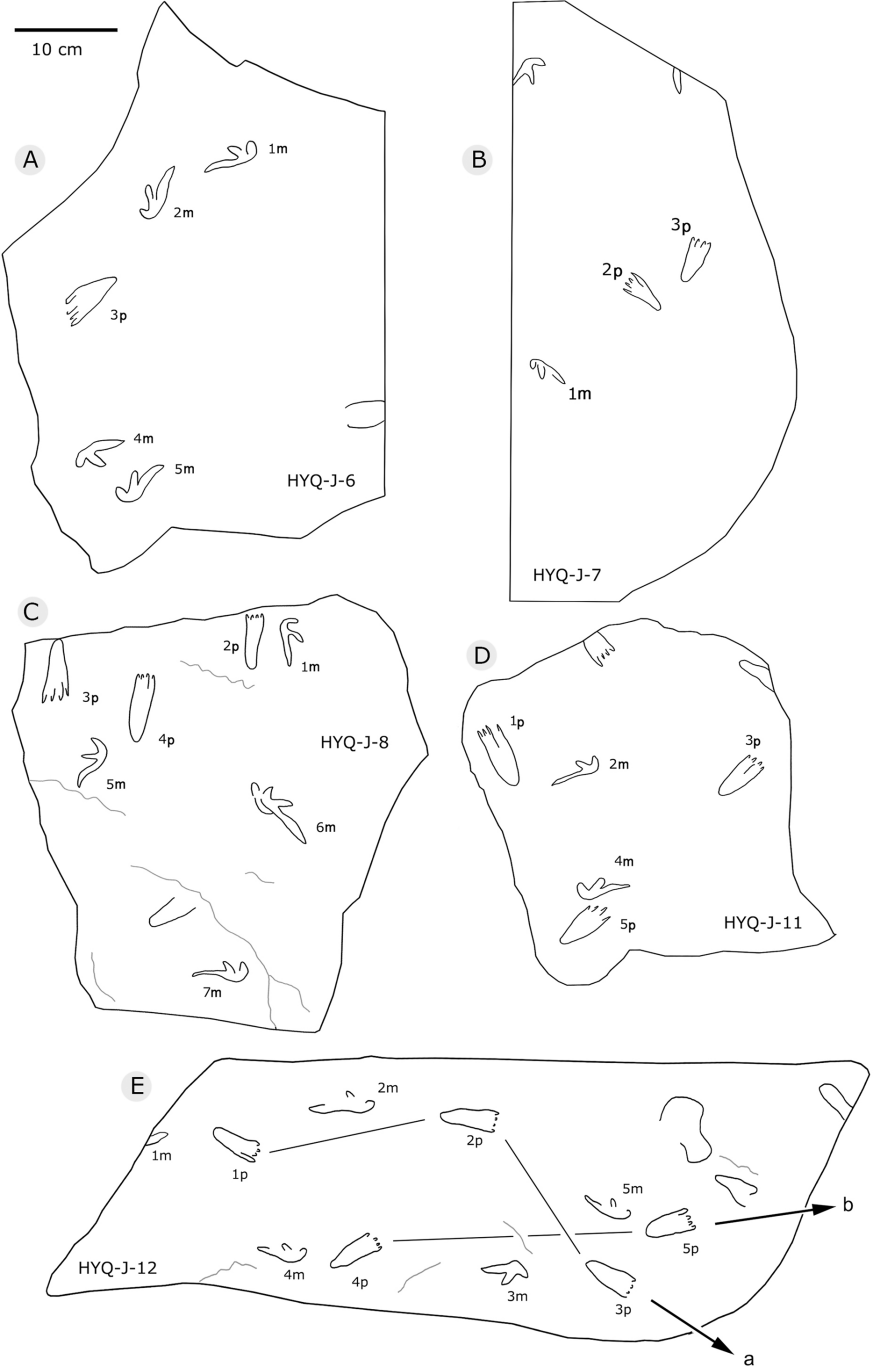
The interpretive outline drawing of pterosaur tracks at Huangyangquan sites: HYQ-J-6 (A), 7 (B), 8 (C), 11 (D), 12 (E).

**Figure 36:**
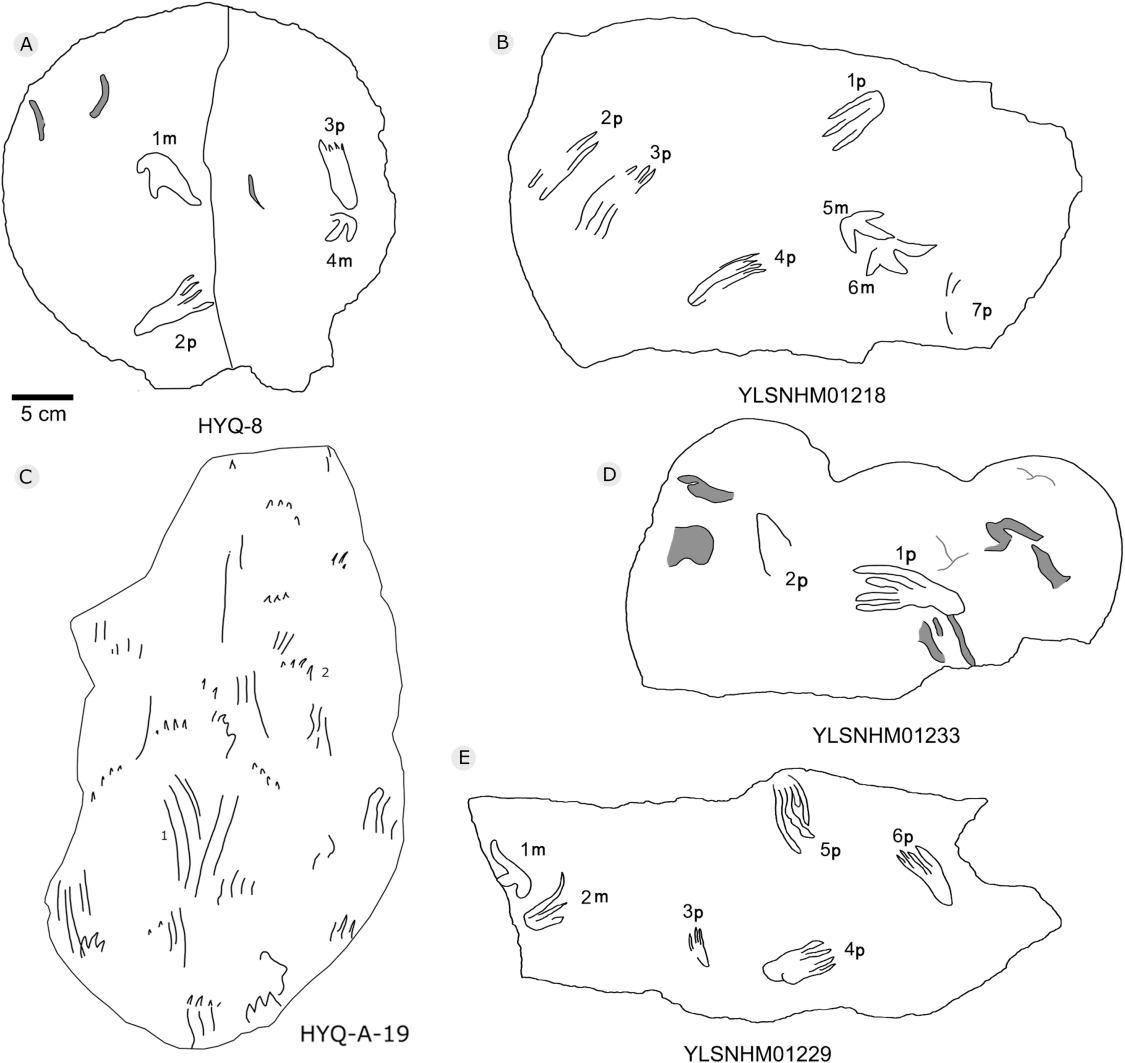
The interpretive outline drawing of pterosaur tracks at Huangyangquan sites: HYQ-8 (A); YLSNHM01218 (B); HYQ-A-19 (C); YLSNHM01233 (D), 01229 (E).

**Figure 37:**
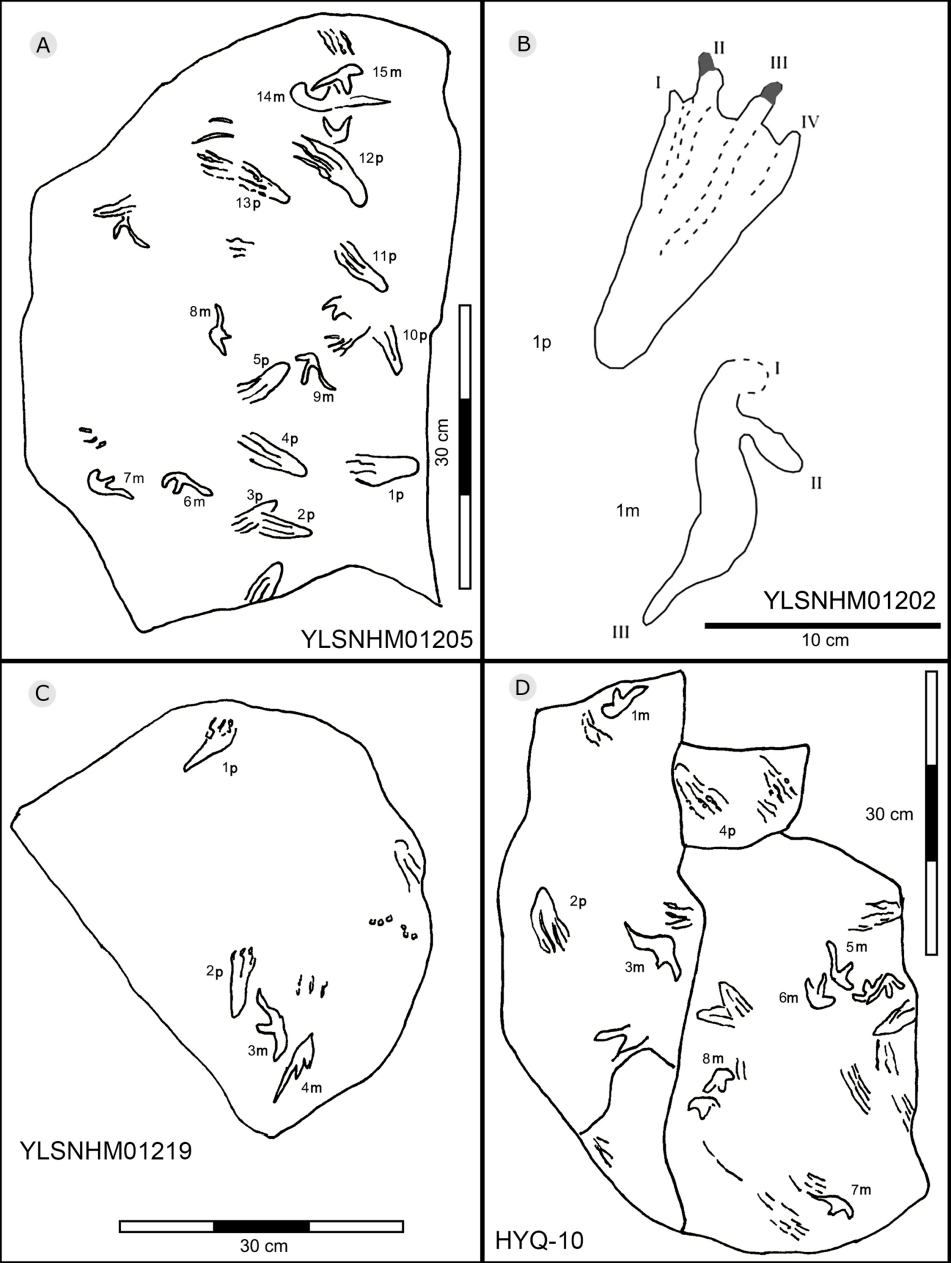
The interpretive outline drawing of pterosaur tracks at Huangyangquan sites: YLSNHM01205 (A), 01202 (B), 01219 (C); HYQ-10 (D).

**Figure 38:**
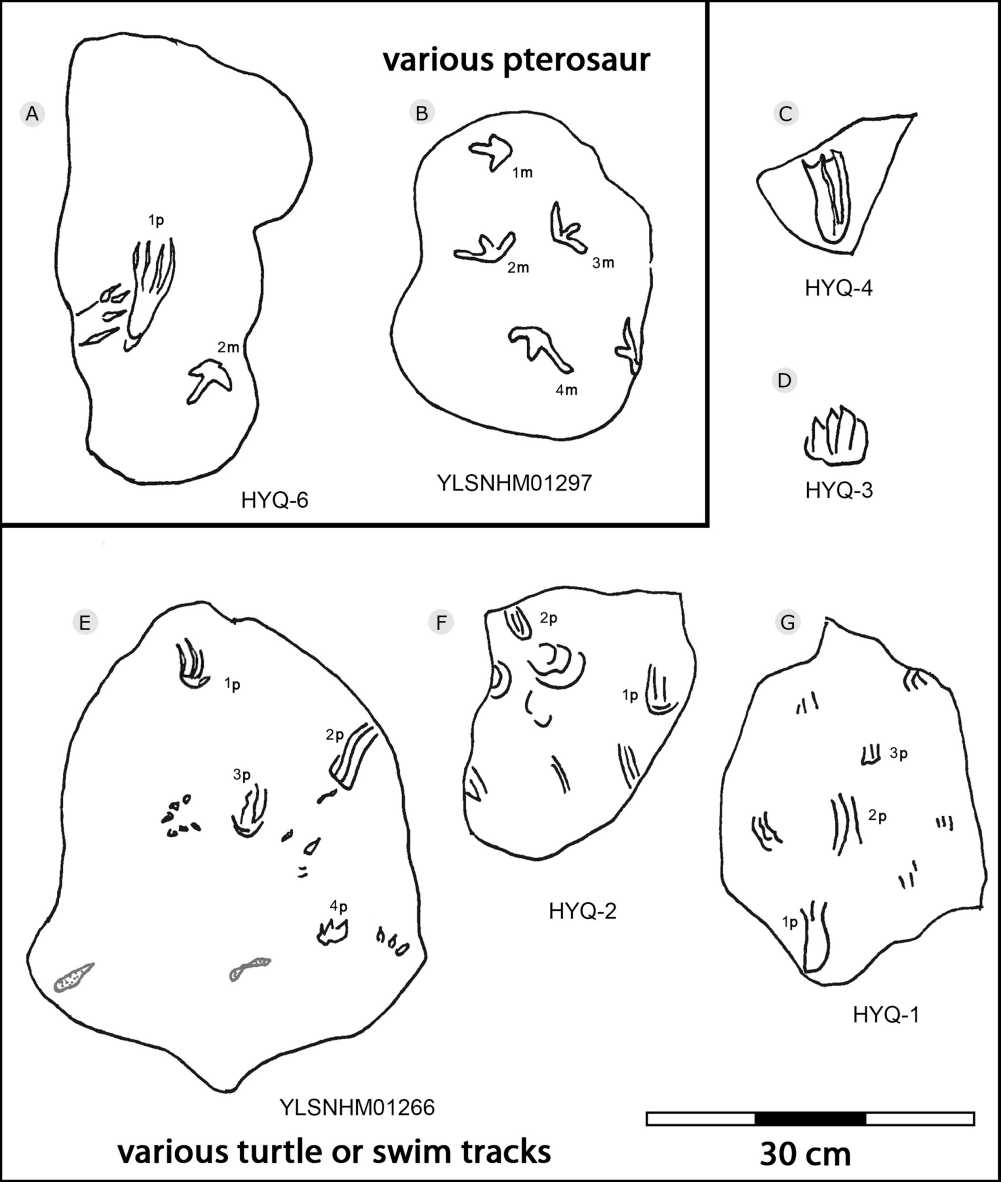
The interpretive outline drawing of pterosaur and turtle tracks at Huangyangquan sites: HYQ-6 (A); YLSNHM01297 (B); HYQ-4 (C), 3 (D); YLSNHM01266 (E); HYQ-2 (F), 1 (G).

197 pterosaur tracks were counted in the Huangyangquan area (see Table 1 for detailed list, and Supplement Information 2 for the measurements) (Figs. 30–38). There are 98 manus prints, suffixed with m, and 99 pes prints, suffixed with p. Pairs of footprints and trackways are very rare, with HYQ-A-7, HYQ-7, HYQ-J-12 and being the best examples (Figs. 31, 32 and 35 respectively).

The sizes of the manus prints ranges from 2.5 cm to 12.3 cm (8 tracks are 2.5–4.0 cm long, 28 are 4.1–5.0 cm long, 36 are 5.1–6.0 cm long, 16 are 6.1–7.0 cm long, and 8 are >7.1 cm long). The length/width ratios range 0.8–4.3 (averaging 2.3): 2.0–2.4, 39 tracks; 2.5–3.0, 30 tracks. The average interdigital divarication angles of Digit I–III is 103°. The mean length ratio of digit I–III is 1: 1.4: 2.1. The mean mesaxony is 0.19, concentrating on 0.10–0.20 (55 tracks).

All manus prints are dominated by one morphotype. Taking the best preserved HYQ-A-25-7m (Fig. 30) as an example, the length is 7.2 cm and the length/width ratio is 2.9. The strongly asymmetrical manus print has three digit impressions. Digit I is shortest, widest, posterolaterally oriented, and curved. Digit II is nearly straight and posterolaterally oriented. Digit III is straight, the longest, and oriented posterolaterally with a distal curvature toward the medial side. The distal ends of digits I–III preserve sharp claw impressions. Digit I and II lack pad impressions, digit III has ~3 elongate phalangeal pads. There is a rounded embossing at the junction of digits II and III, possibly representing the proximal end of digit IV. The length ratio of digit I–III is 1: 1.4: 2.3. The divarication angle of digit I and digit II is 43°, which is lower than that between digit II and digit III (66°). The total divarication in the specimen is large (109°).

However, some tracks show variation in shape such as HYQ-A-5-2m, HYQ-J-5-7m, YLSNHM01218-5m, 6m, YLSNHM01229-2m, YLSNHM01297-1m (Figs. 32, 36, and 38, respectively). Nevertheless, their main morphological features are identifiable. The manus prints are asymmetrical, digitigrade, and consist of three digit impression casts. All three digit casts radiate from a central bulge, and the bulge of digit III is the longest. In some cases, digit II is the longest, and displays a form similar to that of a bird track. For example, the mesaxony of YLSNHM01297-1m is 0.54, much higher than the average value. The impressions of manual digits I and II are short and oriented anterio-laterally and posterio-laterally, respectively. These variations in the tracks are probably caused by the condition of the wet, slippery, and easily deformed substrate.

The size of the pes prints range from 1.6 cm to 14 cm (4 tracks are ≤4.0 cm long, 9 are 4.1–5.0 cm long, 35 are 5.1–6.0 cm long, 25 are 6.1–7.0 cm long, 15 are 7.1–8.0 cm long, 8 are >8.1 cm long). The length/width ratio ranges from 0.6 to 3.6 (averaging 2.4). There are 42 tracks with a ratio of 2.0–2.4 and 28 tracks of 2.5–3.0. According to size, length/width ratio and morphology, these tracks can be divided into 2 morphotypes: A and B.

Morphotype A is represented by YLSNHM00974-1p (Fig. 30) and YLSNHM01202-1p. (Fig. 37) YLSNHM01202-1p is 14 cm in length, with a length/width ratio of 2.3. The pes imprint is plantigrade with four slender digit impressions and indistinct digital pads. It is sub-triangular in overall shape. Due to the presence of corresponding manus prints, digits I–IV of the pes prints can be distinguished. All digits are straight. The distance between digits I and II is obviously less than that of II–III, III–IV. Digits II and III are longest, the most forward projecting, and subequal in length. Digits I and IV are short. Claws are distinct in digits II and III, that of digit III (9 mm) is slightly longer than that of digit II (8 mm). The divarication angle between digits I and IV is 25°. The metatarsal area is elongate, showing a sub-triangular outline.

YLSNHM00974-1p is 6.3 cm in length, with a length/width ratio of 2.1. The pes imprint is similar to YLSNHM00974-1p in overall morphology. All digits are straight. The distance between digits I and II is obviously less than that of II–III and III–IV. Digit I is longest. Digits II and III are subequal in length. Claws are distinct in digits III and IV. The divarication angle between digits I and IV is 24°. The metatarsal area is elongate showing a sub-triangular outline.

Some poorly preserved pes prints mainly differ from YLSNHM00974-1p and YLSNHM01202-1p in the number of digits. These tracks are missing the digit I and digit II boundary, displaying tridactyl appearance. Some tracks with worse preservation only have outlines of heels, such as HYQ-J-4.

Morphotype B is a trace caused by subaqueous rowing, obviously left by a swimming trackmaker that had the same foot morphology as the terrestrially walking producer of morphotype A. Morphotype B consists of elongate, parallel scratches and claw marks. Specimens include YLSNHM01205, YLSNHM01218, YLSNHM01219, YLSNHM01229, HYQ-6, HYQ-10 and HYQ-A-19 (Figs. 36 and 37).

YLSNHM01205 tracks include the manus and pes prints of pterosaurs. There are no trackways. The tracks are distinct overlaps, and some badly preserved scratches. The distal end of 13p overlapped with another pes print, and destroyed the heel of the latter. The small track (15m) overlapped with the large track (14m). Similar to YLSNHM01205, HYQ-10 also has several overlapping manus and pes prints and clear scratches. Both have four elongate and parallel traces with the same direction.

YLSNHM01218 preserves two manus prints (5m and 6m) that show some extramorphological variation, and one badly preserved pes print (1p) with only three digit traces. It is notable that 2p, 3p and 4p all have elongate, parallel claw marks in the same direction. The length of 2p, 3p and 4p is 7.1, 8.1 and 7.0, and the length/width ratio is 3.2, 2.7 and 3.3, distinctly different from that of 1p (2.0, 6 cm in length). YLSNHM01229 preserves one track (5p) with only digit traces, the length of each of the four digit traces is 6.1 cm, the length/width ratio is 2.8, and there is no heel trace.

The tracks of HYQ-A-19 (Fig. 36) are all elongate scratch traces (HYQ-A-19-1) and short claw marks (HYQ-A-19-2). HYQ-A-19-1 is 9.2 cm in length, with a length/width ratio of 3.5. It consists of four scratch traces, three are deep and long, and one is shallow and short. HYQ-A-19-2 is 1.6 cm in length, with a length/width ratio of 0.6, and preserves claw marks.

As noted above, there are few clearly recognizable pterosaur trackways in Huangyangquan area, HYQ-J-12 and HYQ-7 are relatively well-preserved. HYQ-J-12 preserves two trackways, 1p and 1m–3p and 3m sets compose trackway a, and 4p and 4m–5p and 5m sets compose trackway b. All the pes impressions are positioned anterior to the manus impressions. The average length of the pes prints of trackway a is 5.2 cm, the mean pace length is 20.8 cm (four times the length of the footprint), the pace angulation is 130°, and the pes prints are rotated 7° outwards towards the axis of the trackway. The average length of the manus prints is 5.2 cm, the mean pace length is 23.8 cm (4.6 times the length of the footprint), and the pace angulation is 116°. The inner trackway width is 10 cm, and the outer trackway width is 16.7 cm. Trackway b only preserves right tracks, the mean length of the pes prints is 5.2 cm and the pace tracks, each 31.9 cm long (6.13 times the length of the footprint).

HYQ-7 preserves five successive pes prints, designated 1p–5p, without corresponding manus prints. The mean length of the tracks is 6.9 cm, the mean pace length is 13.4 cm (1.94 times the length of the footprint), the pace angulation is 119°, and the pes prints are rotated ~24° (N=4: 15°, 14°, 18°, and 48°) outwards towards the axis of the trackway.

### Comparison & discussion

He et al. (2013) described a set of pterosaur manus and pes prints from the Wuerhe area, which is the first record in this region, and assigned them to *Pteraichnus*. So far, all pterosaur tracks in China have been assigned to *Pteraichnus*, including those known from the Lower Cretaceous Hekou Formation of Gansu Province (Li et al., 2006a; Zhang et al., 2006) and from the Jimo site in Shandong Province (Xing et al., 2012). Various other pterosaur tracks with limited published details are known from the Dongyang site in Zhejiang Province (Lü et al., 2010), the Qijiang District in Chongqing city (Xing et al., 2015c) and the Zhaojue site in Sichuan Province (Xing et al., 2015b).

The morphology of the Wuerhe tracks is extremely similar to that of *Pteraichnus*. Stokes (1957) reported the first credible pterosaur ichnogenus *Pteraichnus* from the Upper Jurassic. *Pteraichnus* remains the most prevalent and well-represented pterosaur ichnotaxon (Lockley, Harris & Mitchell, 2008). There are many *Pteraichnus* ichnospecies, the validity of most has yet to be proved. The currently recognized ichnospecies are: *P. saltwashensis* (Stokes, 1957), *P. stokesi* (Lockley et al., 1995), *P. longipodus* (Fuentes et al., 2004), and *P. nipponensis* (Lee et al., 2010).

*P. saltwashensis* is the type ichnospecies of *Pteraichnus* (Stokes, 1957) from the Late Jurassic Morrison Formation (Kimmeridgian-Tithonian) in Arizona. It includes slim digits, an elongated triangle-shaped pes, and a gradually curved orientation of the manus digit prints. *P. stokesi* (Lockley et al., 1995) is from the Middle to Upper Jurassic Sundance Formation, Wyoming. Lockley et al. (1995) described *P. stokesi* as differing from *P. saltwashensis* in showing a greater degree (45°) of outward rotation of the pes with respect to the midline of the trackway, a relatively shorter and wider third manual digit, and occasional traces of the fifth pes digit. Besides, the pace angulation of the manus print of *P. stokesi* is 90°, smaller than that of *P. saltwashensis* (110°). Sánchez-Hernández, Przewislik & Benton (2009) considered ichnospecies to also differ in the proportional lengths of the manus and pes: in *P. saltwashensis* the manus (9 cm) is longer than the pes (7 cm), whereas in *P. stokesi* the opposite is the case (pes 9 cm; manus 7 cm).

The Huangyangquan specimens are most similar to *P. saltwashensis* (Stokes, 1957) and *P. stokesi* (Lockley et al., 1995). The pace angulation of the manus prints is 116° (HYQ-J-12 trackway a), and the pes prints of the HYQ-J-12 trackway a are rotated 7° outwards towards the axis of the trackway, which is 24° in the HYQ-7 trackway. These traits are similar to those of *P. saltwashensis*, however, the manus of *P. saltwashensis* is longer than the pes, while the manus of the HYQ specimens is subequal to or slightly shorter than the pes. Overall, the Huangyangquan specimens can be assigned to *P. saltwashensis*.

He et al. (2013) considered that the Wuerhe pterosaur trackmaker was most likely a small to medium-sized pterodactyloid based on the lack of a pedal digit V impression. Pterosaur fossils from Junggar basin include the species *Dsungaripterus weii* Young, 1964, *Noripterus complicidens* Young, 1973a, and *Lonchognathosaurus acutirostris* Maisch, Matzke & Sun, 2004, which is likely an individual of *Dsungaripterus weii* (Andres, Clark & Xing, 2010). Pterosaurs known from the Wuerhe area consist of only dsungaripterid pterodactyloids. A pterosaur skull was found in the pterosaur track-bearing beds at the Huangyangquan Site J, and it exhibited the iconic and diagnostic crest of *Dsungaripterus*. Therefore it can be speculated that the trackmaker of the Huangyangquan *Pteraichnus* belongs to dsungaripterid pterosaurs.

Morphotype B is quite similar to the Late Jurassic pterosaur swimming tracks described by Lockley & Wright (2003) from Utah in having narrow scrape marks with the same directional orientation and the missing or very shallow impression of the heel and proximal part of the footprint. The scrape marks of the pes prints also show similar interdigital space as those of morphotype A. The distance between digits I and II traces is clearly shorter than that of digit II–III, and III–IV traces, similar to the HYQ-10 specimen.

Lockley & Wright (2003) considered these narrow scrape marks to have probably been made by a buoyant or partially buoyant pterosaur impressing most of the distal part of its foot on the substrate while progressing in shallow water. The digit region of these tracks is much deeper and clearer than in any known walking pes tracks. This suggests that the foot registered on a very soft, ductile but cohesive substrate, as might be found in a shallow subaqueous setting. Similar elongate scrape marks were described by Lockley et al. (2014), from the Cretaceous of Colorado.

The scratches of morphotype B are not directly comparable with the “normal” shape of pes traces. The pterosaur left footprints of "normal" shape when the whole foot contacted the substrate. When the water level was high enough to sustain buoyancy, only the pterosaur’s toe tips could reach the bottom.

### Turtle tracks

#### Description

**Figure 39:**
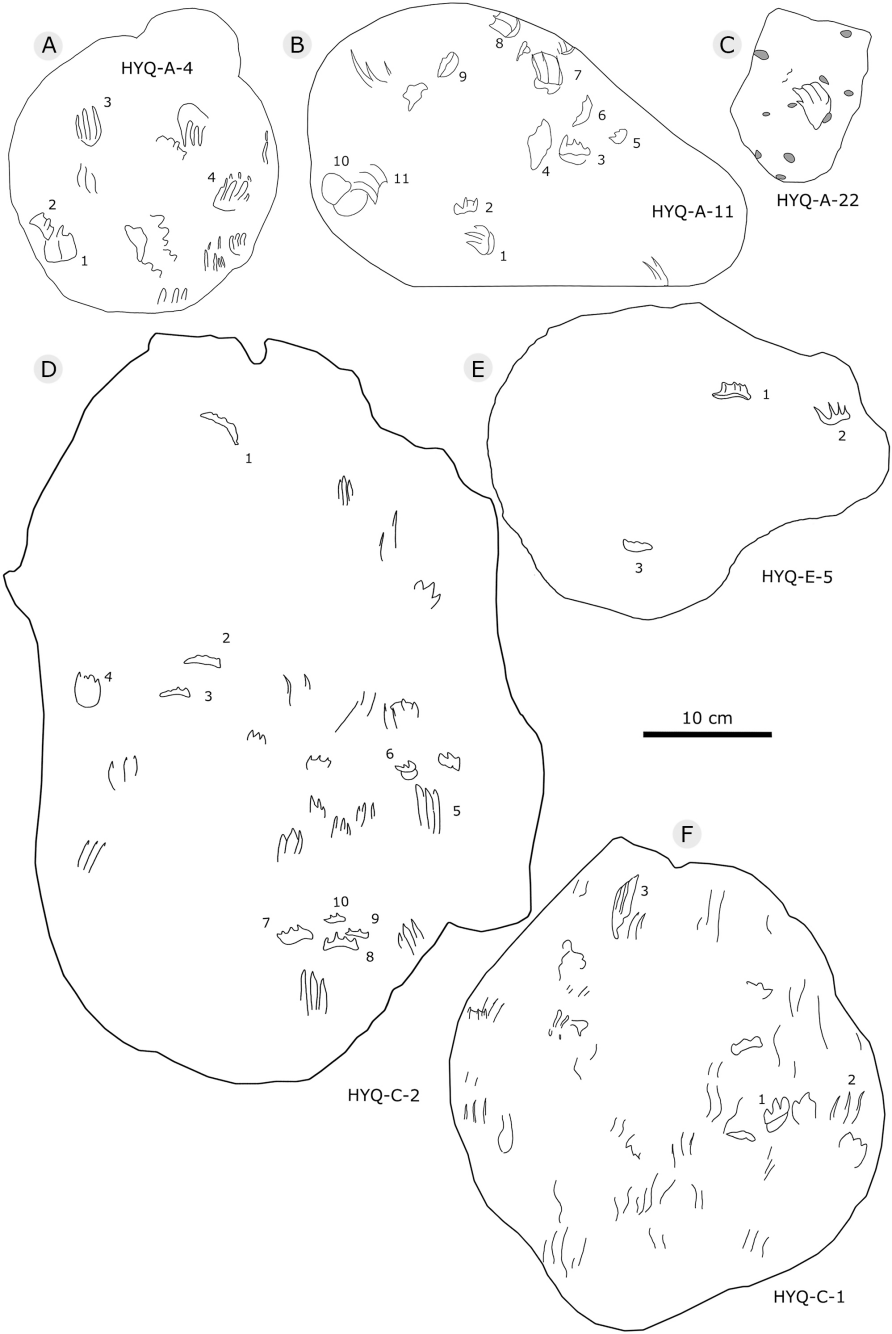
The interpretive outline drawing of turtle tracks at Huangyangquan sites: HYQ-A-4 (A), 11 (B), 22 (C); HYQ-C-2 (D); HYQ-E-5 (E); HYQ-C-1 (F).

**Figure 40:**
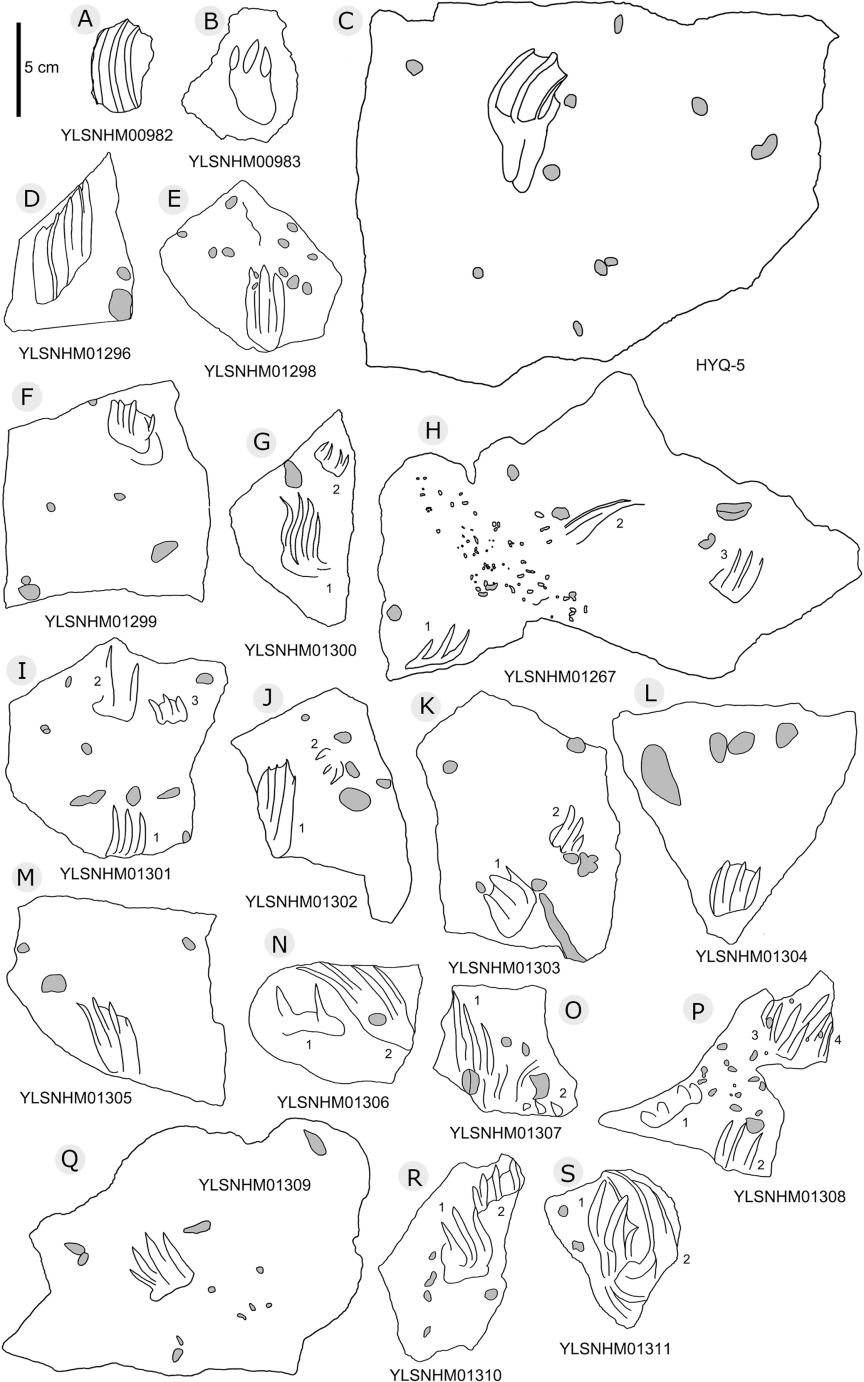
The interpretive outline drawing of turtle tracks at Huangyangquan sites: YLSNHM00982 (A), 00983 (B); HYQ-5 (C); YLSNHM01296 (D), 01298 (E), 01299 (F), 01300 (G), 01267 (H), 01301 (I), 01302 (J), 01303 (K), 01304 (L), 01305 (M), 01306 (N), 01307 (O), 01308 (P), 01309 (Q), 01310 (R), 01311 (S).

**Figure 41:**
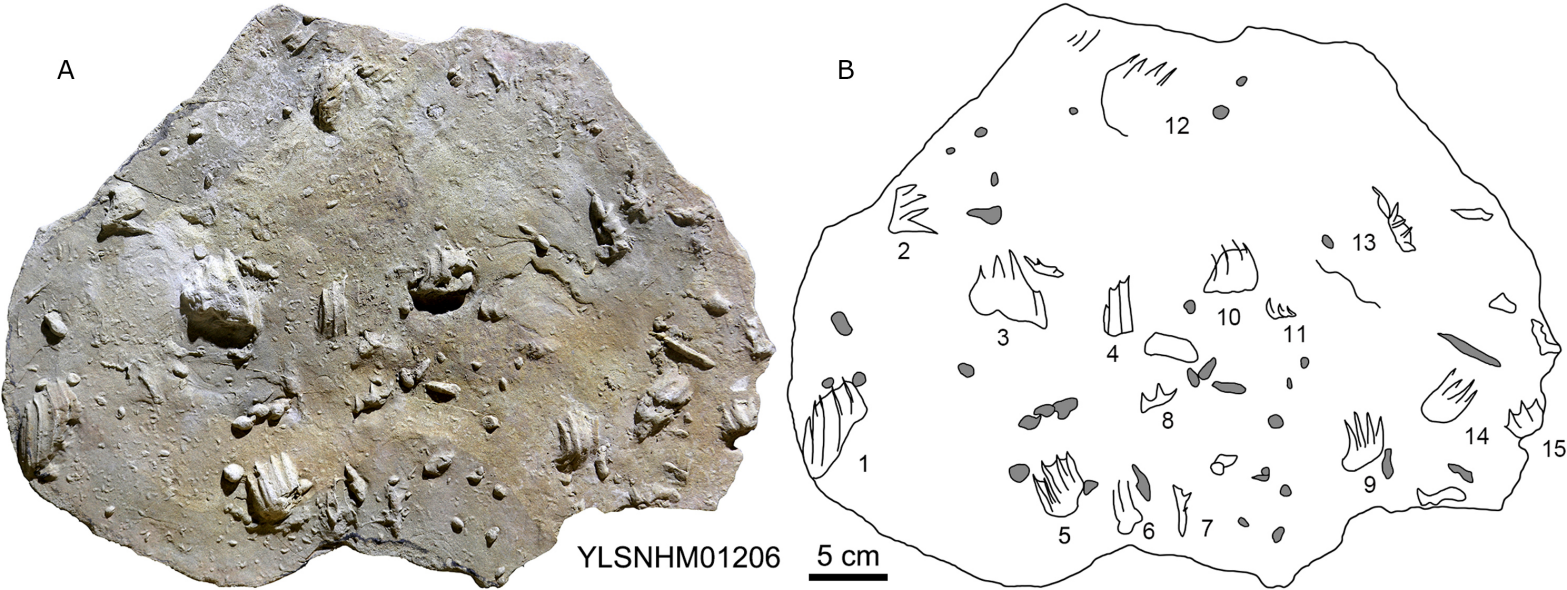
Photograph (A) and interpretive outline drawing (B) of turtle tracks YLSNHM01206 at Huangyangquan sites.

**Figure 42:**
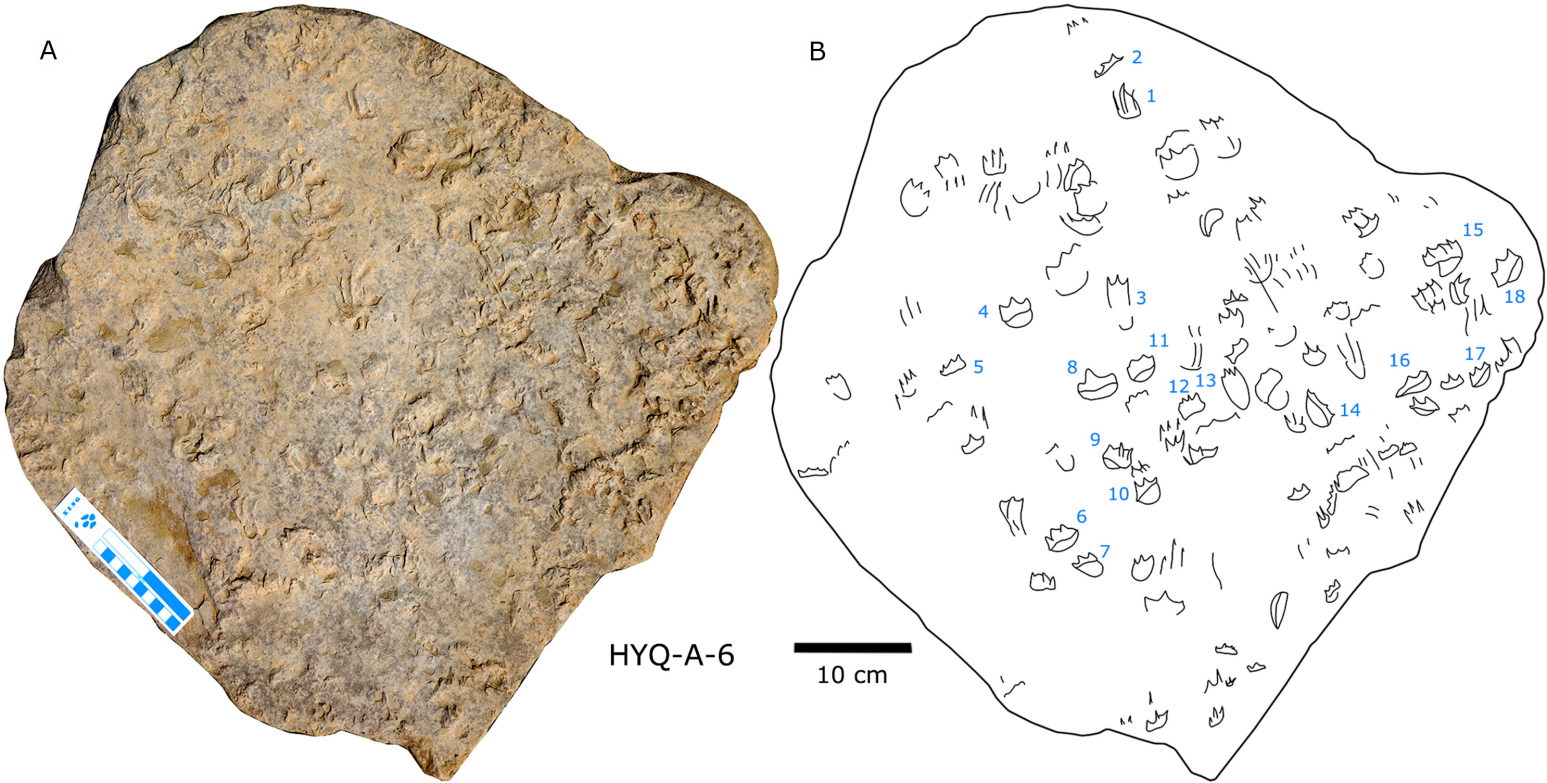
Photograph (A) and interpretive outline drawing (B) of turtle tracks HYQ-A-6 at Huangyangquan sites.

There are at least 362 turtle tracks in Huangyangquan area, and 108 of them were measured (see Table 1 for detailed list, and Supplement Information 2 for the measurements) (Figs. 5, 38–42). The track length ranges from 0.7 cm to 8.9 cm (8 tracks are ≤ 1.0 cm long, 37 tracks are 1.1–2.0 cm long, 18 tracks are 2.1–3.0 cm long, 20 tracks are 3.1–4.0 cm long, 12 tracks are 4.1–5.0 cm long, 6 tracks are 5.1–6.0 cm long, and 6 tracks are > 6.1 cm long). The average length/width ratio is 1.1 (0.3–2.7), and 41 tracks have a ratio in the range of 0.5–1.0.

These tracks were left by quadrupedal animals. However, the lack of distinct trackway makes it difficult to distinguish the manus from the pes print. All the tracks are tridactyl to pentadactyl, with digit traces facing anteriorly and showing little interdigital divarication. The digit traces are mostly elongated into scratch marks and furrows that can be interpreted as claw impressions. All digits are connected by a posterior arched structure slightly less impressed into the substrate than the claw impressions. According to the differences of size, length/width ratio, and morphology, these tracks can be divided into three different morphotypes: A, B, and C.

Morphotype A is best represented by HYQ-3 (Fig. 38). HYQ-3 is 5.3cm in length, with a length/width ratio of 0.9. HYQ-3 is typically digitigrade, and has four elongated ungual marks; the marks are roughly parallel and face anteriorly. The middle ungual marks (digits II and III) are longer than the lateral two (digits I and IV). The similar track, HYQ-A-11-2 (Fig. 39), is 1.2 cm in length, with a length/width ratio of 0.7, and only has three ungual marks -- probably missing a relatively shallow impression of the lateral digit. The medial digit is the longest, while the lateral digits are subequal. The similar track assemblage YLSNHM01206 (Fig. 41) indicates a mean length/width ratio of 1.1, range from 0.3–1.9.

Morphotype B is represented by HYQ-4 (Fig. 38), which is 8.9 cm in length, with a length/width ratio of 2.3. HYQ-4 is typically digitigrade, or semiplantigrade and has elongated ungual marks. Morphotype B most significantly differs from morphotype A in the higher length/width ratio and the number of digits. HYQ-4 has three ungual marks; they are roughly parallel and face anteriorly. The three ungual marks are all similar in width and length, with the middle ungual mark slightly deeper than the lateral two.

Morphotype C shows elongate scratches, such as in HYQ-2 and YLSNHM01266 (Fig. 38), HYQ-C-1 (Fig. 39), YLSNHM01267-2, YLSNHM01306-2, YLSNHM01311-1 and 2, (Fig. 40), and HYQ-A-6 (Fig. 42). The claw impressions extend into 3–5 parallel scratch marks that extend anteriorly, up to several times the length of the footprint. Some specimens have only parallel scratch marks, without heels.

### Comparison & discussion

Lockley, Matsukawa & Li (2012) summarized three discoveries of turtle tracks in the Lower Cretaceous of China: (1) the Chabu tracksite in the Jingchuan Formation of Nei Mongol, (2) the Zhucheng tracksite in the Long-wangzhuang Formation of Shandong Province, and (3) the Huangyangquan tracksite in the Tugulu Group of the Wuerhe district in the Xinjiang Uyghur Autonomous Region. Collectively, these represent the first reports of turtle tracks from the Mesozoic of China.

Avanzini et al. (2005) considered there to be only two ichnotaxa attributable to turtles: *Chelonipus* (Triassic) and *Emydhipus* (Late Jurassic–Early Cretaceous). *Chelonipus* has been described from strata dating from the Early Triassic, the Late Triassic, and the Late Jurassic (Plieninger, 1838; Meyer & Plieninger, 1844; Von Lilienstern, 1939; Haubold, 1971; Lockley & Foster, 2006; Lovelace & Lovelace, 2012; Lichtig et al., 2018). Lockley, Xing & Xu (2019)reclassified *Laiyangpus liui* (Young, 1960) from the Lower Cretaceous of Shandong Province in China, which had been assigned to *Coelurosauria* in the 1960s, as *Chelonipus liui*. This extended the records of *Chelonipus* to the Cretaceous. *Emydhipus* is known from the Late Jurassic and Early Cretaceous (Fuentes et al., 2003; Avanzini et al., 2005).

Xing et al. (2014a) described the turtle tracks from Huangyangquan area in detail, including 40 complete natural casts on 15 slabs. These specimens were assigned to *Emydipus* isp., and the trackmaker was considered *Xinjiangchelys* sp. or *Wuguia* sp. Abundant turtle body fossils also occur from the Tugulu Group (Ye, 1973; Gaffney & Ye, 1992; Matzke et al., 2004; Danilov & Sukhanov, 2006; Danilov & Parham, 2007).

*Emydhipus* is characterized by its four elongated claw marks in the manual print, which are parallel to each other and to the midline of the trackway. The pedal print is plantigrade and has four clawed digits and a round short sole (Fuentes et al., 2003; Avanzini et al., 2005; Klein et al., 2018).

The Huangyangquan morphotypes A and B differ in the length/width ratio and the number of digit traces. Xing et al. (2014a) assigned most morphotype A tracks to pes prints, and most morphotype B tracks to manus prints. However, until a definite trackway is found, there is no confident way to distinguish with confidence between manus and pes prints. Xing et al. (2014a) identified one possible trackway, and considered the pes print to be anteromedial relative to the corresponding manus print. However, the trackway is too short, to be confident. No unequivocally-recognizable trackway was identified in new materials. Nonetheless, the elongated scratch marks in Morphotype B on the manus prints are very similar to those typical of *Emydipus*.

Morphotype C is similar to pterosaur swimming traces, especially HYQ-A-19 (Fig. 36). The differences between the two include: three scratch traces in turtle morphotype C, while the number is four in pterosaur swimming traces; and an interdigital arche in Morphotype C, which is absent in the pterosaur. Lockley et al. (2014) outlined criteria for distinguishing between turtle, crocodylian and pterosaur swim tracks, which is possible in some but not all cases, and dependent on various factors including the size and completeness of tracks, relative length and width of digit traces and relationships between tracks in assemblages.

### Asphaltite site

**Figure 43:**
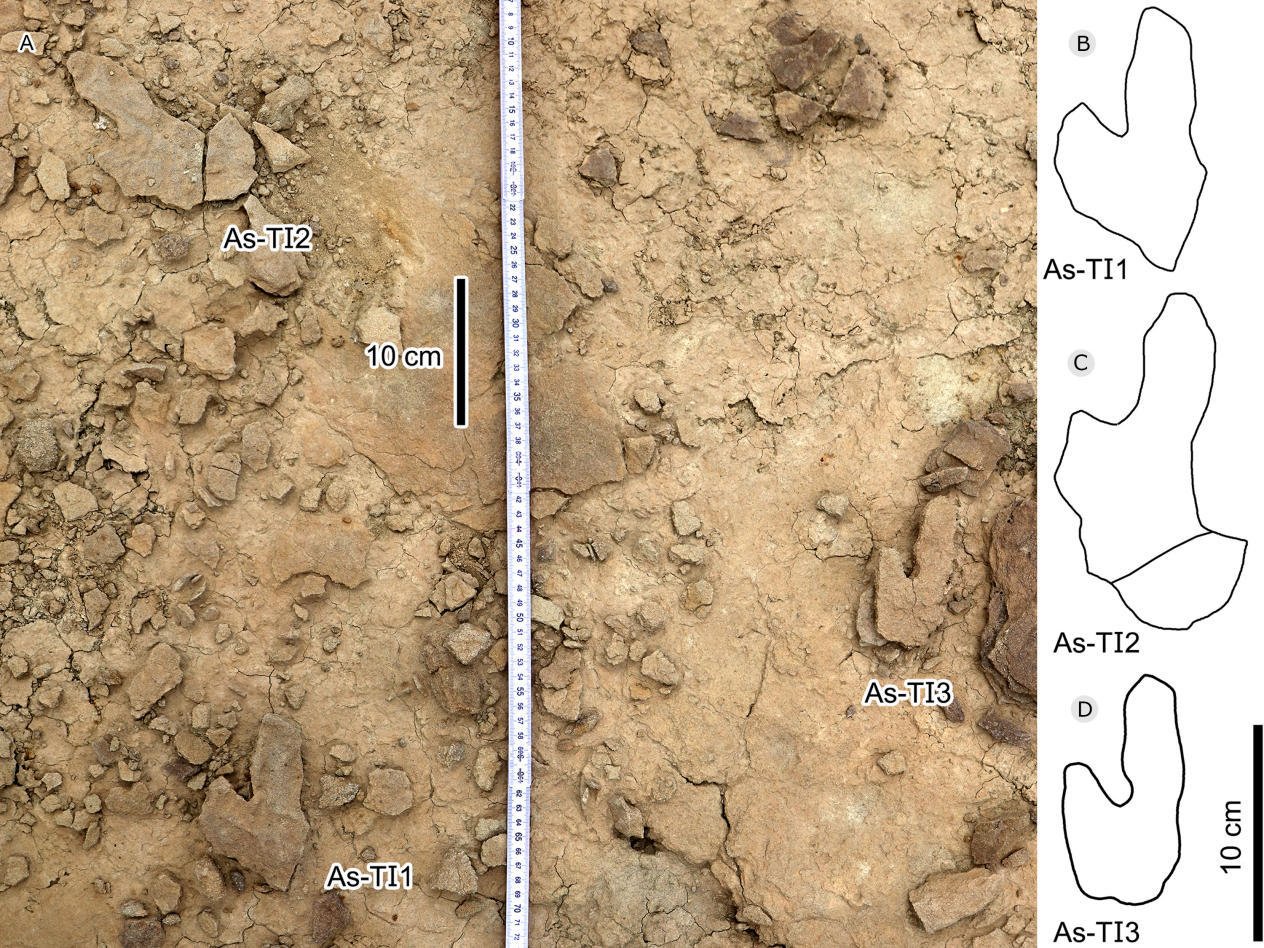
Photograph (A) and interpretive outline drawing of theropod tracks As-TI1 (B), 2 (C), 3 (D) at Asphaltite sites.

Xing et al. (2013b) described non-avian and avian theropod and pterosaur footprints from the Lower Cretaceous asphaltite site. Three incomplete tracks were discovered in the Asphaltite area during the 2019 investigation. These tracks are natural casts, designated As-TI1–TI3 (Fig. 43), with lengths of 18.1, 23.4 and 16.2 cm and a thickness of 1–1.5 cm. Each preserves two digits. There is no distinct trackway. Didactyl tracks are attributed to deinonychosaurian (dromaeosaurid and troodontid) trackmakers, however, As-TI1–TI3 lack the main features of deinonychosaurian trackmakers, such as subequal digit III and IV. Though the deinonychosaurian tracks considered to belong to troodontids, such as *Menglongipus* (Xing et al., 2009b), have a shorter digit IV, As-TI1 and TI2 of As-TI1–TI3 are apparently damaged. Therefore, As-TI1–TI3 may represent a tridactyl track missing one lateral digit. Generally, digit IV is relatively shallow, due to the weight distribution of the trackmaker. As-TI1–TI3 appear to have well-developed heels, which is similar to the *Jialingpus* tracks from the same area.

The preservation of the tracks is unusual. The lower level, is a light-yellow muddy siltstone (Layer 1). Layer 2 is a layer of mudstone ~1 cm thick. Layer 3 is a light-brown muddy siltstone where the tracks are located. Presumably, the footprints registered on Layer 2 and were filled with silty sediments to form casts, then Layer 3 disintegrated due to later weathering and the tracks were exposed.

### Wuerhe vertebrate fauna

#### Paleoecology

**Non-avian theropod:** Most *Jialingpus* tracks from site A, D, and I are isolated casts, which presumably formed by water flow in the reservoir continuously eroding the outcrop. However, there remains some evidence to show the abundance of theropod tracks in the Huangyangquan area. For example, HYQ-D-4 shows partly overlapping tracks, and there are approximately 81 theropod tracks (HYQ-D-13) on a surface of ~7.3 m^2^ (Fig. 11). Over 80% of the tracks are orientated from northwest to southeast, and ~10% of the tracks from southwest to northeast. Only two trackways (five tracks in total) are discernible.

HYQ-A-17 preserves *Jialingpus* (HYQ-A-17-2), turtle tracks (HYQ-A-17-1) and numerous scratch traces left by turtles (Fig. 5). HYQ-A-17-2 is overlapped by scratches. Despite the poor preservation and ambiguous phalangeal pads, there is no sign of theropod swimming traces. A similar situation occurs in HYQ-A-20 (Fig. 5), where turtle tracks were left when water first covered the area and, once the water receded, tracks were left.

Non avian theropod tracks and stegosaur tracks have always been discovered on the same bedding surface. *Jialingpus* of specimens HYQ-D-1, YLSNHM01237 and YLSNHM01247 cooccur with avian theropod tracks. *Jialingpus* of specimen HYQ-J-10 cooccurs with avian theropod and pterosaur tracks. HYQ-7, shows cooccurring pterosaur and avian theropod tracks. He et al. (2013) considered the Wuerhe pterosaur tracks to frequently occur alongside those of birds, but not with non-avian theropod or stegosaur tracks, and suggested that these lacks of cooccurrence probably reflect difference in substrate properties and local paleoenvironments.

Many *Scoyenia* traces cooccur with the *Jialingpus* in HYQ-A-3, which are overlapped on the former. HYQ-A-3-1 shows fairly distinct external morphological changes, the length/width ratio of the anterior triangle is as low as 0.14, which may be due to wet sediments. *Jialingpus* track specimens YLSNHM01277 and YLSNHM01278 cooccur with numerous invertebrate scratch traces. There is no evidence to suggest that the *Jialingpus* trackmakers fed on invertebrates, but this possibility cannot be ruled out.

Theropod hip height is estimated as 4.5 times foot length for small theropods (foot length less than 0.25 m) and 4.9 times foot length for the large theropods, as proposed by Thulborn (1990). For all theropods, body length estimated at about 2.63 times hip height (Xing et al., 2009a). The body length of the theropods in HYQ area accordingly ranges from 0.6 m to 4.1 m, and concentrates at 1.3–1.9 m (corresponding to the track length of 10.7–16.1cm).

**Avian theropod:** Most avian-track-bearing slabs, such as HYQ-E-2, HYQ-12, 16, 17, 18, YLSNHM01292, YLSNHM01231, preserve abundant invertebrate traces, which are dominated by *Helminthopsis* and *Lockeia*. The former is probably produced by polychaetes or priapulids (Fig. 27, Książkiewicz, 1977; Fillion & Pickerill, 1990), and the latter is attributed to terrestrial bivalves (nonmarine bivalves) (Radley, Barker & Munt, 1998). The stellate or rosetted traces associated with specimen YLSNHM01241 (Fig. 21) are not recorded in other specimens. The tracemaker is not known but presumably an infaunal invertebrate (Domichnia / Fodichnia) with a radial foraging capability. Such invertebrates may have been a potential food resource for shore birds.

The trackmaker size range and inferred ichnotaxonomy of Avipedidae (*Aquatilavipes*) and Ignotornidae (*Ignotornis*) is comparable to the *Koreanaornis*-*Goseongornipes*-*Ignotornis* assemblages from the Jindong and Haman formations of South Korea (Lockley et al., 2006b, 2012, 2020).

**Pterosaur:** The Huangyangquan pterosaur tracks constitute the largest number of pterosaur tracks in any Chinese assemblage. Pterosaur and bird track co-occurrences are not rare. Similar records also occur in the Cretaceous strata of China, South Korea and America. For example, the recently opened (November 2019) Jinju Pterosaur Tracks Museum, Jinju City Korea, celebrates the large number of pterosaur tracks recovered from the Lower Cretaceous lacustrine deposits of the Jinju Formation (Kim et al., 2016, 2019). Early Cretaceous pterosaur tracks are also known from terrestrial, lacustrine deposits in the Hekou Formation of Gansu Province (Zhang et al., 2006), the Jiaguan Formation of Chongqing Municipality in China (Xing et al., 2013e). Large pterosaur tracks (*Haenamichnus*) are also reported from Upper Cretaceous lacustrine deposits of Uhangri Formation of South Korea (Hwang et al., 2002). Marginal marine pterosaur track assemblages are known from, the Cenomanian Dakota Group of Colorado (Lockley et al., 2014) and the Campanian North Horn assemblage of Utah (Lockley, 1999). This indicates that tracks of pterosaurs and birds often co-occur along lacustrine and marine shorelines.

On the basis of skeletal anatomy, Bakhurina & Unwin (1995) suggested that beginning from the Cretaceous, pterosaur shifted their habitats to more terrestrial environments . Whereas Jurassic pterosaur tracks are most frequently found in regionally-extensive marine coastal plain sedimentary environments in the Late Jurassic of North America and Europe (Lockley et al., 1995; Lockley & Meyer, 2000; Mazin et al., 2003), Cretaceous pterosaur tracks most frequently occur in non-marine facies, usually fluvio-lacustrine environments in China and Korea (Kim et al., 2006, and references herein). Although they also are found in marine shoreline settings in the Cretaceous of the US western interior (Lockley et al., 2014). The Wuerhe pterosaur tracks however, are another instance, typical of China’s interior terrestrial settings with lake basin facies shorelines. Wuerhe bird and pterosaur track-bearing slabs such as HYQ-A-1, A-2, A-12, A-26, are frequently associated with abundant *Helminthopsis*, *Lockeia* and other invertebrate traces that would benefit from more detailed study.

**Turtle:** Most Huangyangquan turtle tracks have long parallel scratches, which were interpreted as evidence of a partially to fully buoyant trackmaker that swam close to the bottom of a water-filled channel (Milner, Lockley & Kirkland, 2006; Mickelson et al., 2006; Lovelace & Lovelace, 2012; Lockley et al., 2014).

Recent studies of the mid Cretaceous Dakota Group in the western United States have demonstrated the presence of turtle swimming tracks in close association with crocodilian and pterosaur swimming tracks (Lockley et al., 2010, 2014). The nature of swimming traces is variable and generally incomplete, making them difficult to identify. The Huangyangquan turtle tracks, especially some large specimens, are quite similar to pterosaur swimming traces. Generally only the presence of the well-preserved pterosaur or turtle tracks on the same surfaces can be relied upon to confidently differentiate the trackmakers. There is a wide range of the widths of turtle tracks, which suggests a wide range of the body sizes.

**Lakes and the fauna:** In recent decades large numbers of lacustrine basin tetrapod ichnofaunas have been reported from east Asia, particularly from Korea and China. A notable feature of these ichnofaunas is their high diversity, of both large and small forms. Larger forms include widely distributed theropods, sauropods and ornithopod tracks, with stegosaur tracks restricted to the Wuerhe area. Smaller track morphotypes include abundant and diverse small theropod tracks, including small grallatorids, *Minisauripus* and small dromaesaurid morphotypes, diverse bird, pterosaur and turtle tracks in both China and Korea. In these categories the ichnotaxa are similar. Rarer elements found only in Korea include lizard, frog, mammal and crocodylian tracks. These differences reflect different lacustrine systems and facies.

The paleoclimate reconstruction of the Early Cretaceous in East Asia indicates a typically seasonal “semiarid” climate (Houck & Lockley, 2006). Abundant swimming traces left by water-loving animals, such as water/shorebirds, pterosaurs and turtles, indicate that the lakes of Huangyangquan tracksite were perennial (Zhao, 1980; Paik, Kim & Lee, 2001), with variable input of finer and coarser grained clastic sediment indicating expected fluctuations in energy regime and water levels. The Wuerhe assemblages contain many small specimens and few large surfaces. Thus, long trackway segments are rare. Nevertheless, it is clear that the facies yields a high density of tracks. During the dry or drier seasons, the lake basin shrunk so that the lakeside tracks dried out and hardened. Abundant sediments carried by summer rainstorms in upland areas were transported into stream valleys, and periodically overlapped the exposed tracks (Zhao, 1980). The sediments preserved not only the tracks, but also the remains of lakeside animals, such as pterosaurs and turtles found in nodules.

Ever since the vertebrate ichnofacies concept was first introduced (Lockley, Hunt & Meyer, 1994) and further explored by Hunt & Lucas (2007, 2016) and Lockley (2007) there has been widespread recognition that the shorebird ichnofacies (sensu Lockley, Hunt & Meyer, 1994; Melchor, Cardonatto & Visconti, 2012) referred to as the shorebird ichnocoenosis (Hunt & Lucas, 2007) is one of the most distinctive of all tetrapod ichnocoenoses/ichnofacies. Despite some subtle debates over ichnofacies definition, there is little doubt that the Wuerhe ichnofauna represents a classic example of a shorebird ichnocoenosis/ichnofacies. According to Hunt & Lucas (2007: Table 3) the shorebird ichnofacies (sensu Lockley, Hunt & Meyer, 1994) is better considered a “shorebird ichnocoenosis” and a component of / or under the larger global or archetypal umbrella of the *Grallator* ichnofacies concept. This ichnofacies is defined as representing “medium-high diversity ichnofaunas (5–8 ichnogenera) with tracks (usually dominant) of bipedal tridactyl avian and non-avian theropods dominated by higher bipedal carnivores” (sensu, Hunt & Lucas, 2007: Table 3). This definition is broad enough to allow most shorebird-dominated assemblages or ichnocoenoses to be explicitly subsumed under the *Grallator* ichnofacies concept, as is possible in the case of the Huangyangquan ichnofauna or ichnocoenosis.

**Table 3:** Comparison of bone records and track records of Wuerhe area, Xinjiang, China. The Huangyangquan sites from the Lower Layer, and the Asphaltite site from the Upper Layer. Black squares indicate the existence of records.

	Upper Layer	Grey- green Layer	Lower Layer
**Fishes** (Lucas, 2001b)	Y		
**Sauropod**:			
cf. *Asiatosaurus mongoliensis* (Dong, 1973b)	Y	Y	
Camarasauridae (Dong, 1973b)	Y		
**Theropod**:			
*Kelmayisaurus petrolicus* (Dong, 1973b; Brusatte, Benson & Xu, 2012)	Y		
*Phaedrolosaurus ilikensis* (*a nomen dubium per Rauhut & Xu, 2005*)	Y		
*Tugulusaurus faciles* (Dong, 1973b)	Y		
*Xinjiangovenator parvus* (Rauhut & Xu, 2005)	Y		
**Theropod tracks**:			
grallatorid	Y		Y
*Jialingpus* isp.	Y		Y
*Asianopodus* isp.			Y
small-sizad *Eubrontes*	Y		Y
**Bird tracks**			
*Aquatilavipes dodsoni*	Y		Y
*Ignotornis* isp.			Y
**Stegosaur**			
*Wuerhosaurus homheni* (Dong, 1973b, 1990)	Y		
*Wuerhosaurus* sp.			Y
**Stegosaur tracks**			
*Deltapodus curriei*			Y
**protosuchian crocodyliform**			
*Edentosuchus tienshanensis* (Young, 1973b; Diego et al., 2004)	Y		
**Pterosaur**			
*Dsungaripterus weii* (Young, 1964)	Y	Y	
*Noripterus complicidens* (Young, 1973a)	Y		
*Lonchognathosaurus acutirostris* (Maisch, Matzke & Sun, 2004)	Y		
*Dsungaripterus* sp.			Y
**Pterosaur tracks**			
*Pteraichnus saltwashensis*			Y
*Pteraichnus* isp.	Y		
**Plesiosauria**			
*Sinopliosaurus weiyuanensis* (Young, 1973c)	Y		
**Turtle**			
"Sinemys wuerhoensis" (Ye, 1973)	Y		
*Dracochelys bicuspis* (Gaffney & Ye, 1992)	Y		
*Ordosemys brinkmania* (Danilov & Parham, 2007)	Y		
*Xinjiangchelys* sp. (Danilov & Parham, 2007; Xing et al., 2014a)	Y		Y
*Wuguia hutubeiensis* (Matzke et al., 2004; Xing et al., 2014a)			Y
*Wuguia efremovi* (= *Dracochelys wimani*) (Danilov & Sukhanov, 2006)	Y		
cf. *Pantryonichia* indet. (Danilov & Parham, 2007)	Y		
**Turtle tracks**			
*Emydipus* isp.			Y

Thus, using these hierarchical options the Huangyangquan ichnofauna would be considered an example of the shorebird ichnofacies by Lockley, Hunt & Meyer (1994) or an example of the shorebird ichnocoenosis within the *Grallator* ichnofacies by Hunt & Lucas (2007, 2016). It is notable in the case of the Huangyangquan ichnofauna that grallatorid tracks are a significant component, although such tracks do not have to be found in order to fit the definition of a shorebird ichnocoenosis or the *Grallator* ichnofacies. The presence of turtle swim tracks indicates that expressions of the *Characichnos* swim tracks ichnofacies are also intercalated with the *Grallator* ichnofacies: the former representing shallow lake settings, the latter representing the emergent shorelines.

**Vertebrate fauna of northwestern Junggar Basin :**The fossil sites of northwestern Junggar Basin mainly include the Wuerhe area and the Delunshan area, which is located 100 km northeast of Wuerhe. The classical Cretaceous basal ceratopsian *Psittacosaurus* (Dong, 1973a; Brinkman et al., 2001) and fragments of *Teratosaurus* have been found in the Delunshan area.

As for the skeletal fossil record, the vertebrate fauna in Wuerhe and surrounding areas includes fish (Lucas, 2001a), sauropods, theropods, stegosaurs, protosuchian crocodyliforms, pterosaurs, plesiosauria and turtles (Dong, 1973b; Young, 1973a, 1973b, 1973c). These higher taxa categories have not changed since summarized by Dong (1973b), but the members keep increasing as shown in the table (Table 3). Most skeletal records are from the Upper Layer of the Tugulu Group, while a few are from the Middle grey-green Layer and Lower Layer. In terms of diversity, turtles (six species) are the most diverse, followed by theropods, pterosaurs, sauropods and, finally, stegosaurs.

In contrast to the bone records, track records were mainly from the Lower Layer (the Huangyangquan site), while a few tracks are from the Upper Layer (the Asphaltite site). In Huangyangquan area, non-avian and avian theropod tracks contain one and two ichnospecies, respectively, and stegosaur tracks, pterosaur, and turtle tracks all contain one ichnospecies.

There are a total of 1,552 tracks at the site, including 30 trackways and 1,445 isolated tracks, potentially representing 1,473 trackmakers in the sample collected to date. Non-avian theropods account for 14.5%, birds account for 40.8%, stegosaurs account for 3.5%, pterosaurs account for 18.3%, and turtles account for 22.9%. Thus, the Huangyangquan tetrapod ichnofauna is archosaur dominated (77.1%), of which two thirds (55.3%) represent avian and non-avian theropods. Turtles represent the largest non-archosaurian component. Generally, the Wuerhe ichnofauna is consistent with other terrestrial tetrapod ichnofaunas other Early Cretaceous tracksites regions in China (Xing & Lockley, 2016).

In the Asphaltite area, non-avian and avian theropod and pterosaur tracks are both represented by only one ichnospecies. There is a total of 44 tracks at the site, all of which are isolated, probably representing 44 trackmakers. Thereinto, theropods account for 63.3%, birds account for 36.4%, and pterosaurs account for 23%. Thus, the Asphaltite ichnofauna is essentially entirely archosaur dominated.

## Conclusions

The tetrapod ichnofaunas of the Tugulu group, from the Huangyangquan Reservoir (Wuerhe District, Karamay City, Xinjiang) and the nearby Asphaltite sites have been studied intermittently during the last decade and have yielded large ichnogical collections representative of life in inland lacustrine settings in the northwestern margin of the Junggar Basin during the Early Cretaceous. To date the Huangyangquan assemblages are represented by more 1,500 identified tracks, mainly preserved on small slabs, which are not conducive to preservation of long trackway segments. These however reveal the high density of tracks preserved in lakeshore settings, including abundant tracks of birds (*Aquatilavipes* and *Ignotonis*), non-avian theropods (grallatorids including *Jialingpus-* and *Asianopodus*-like morphotypes), pterosaurs (*Pteraichnus*) and turtles (*Emydipus*). Tracks of stegosaurians (*Deltapodus*) also occur, but are less common and represent the only ichnogical evidence of large quadrupedal dinosaurs currently known.

The Huangyangquan and Asphaltite ichnofaunal associations with smaller tetrapods (avian and non-avian theropods, pterosaurs and turtles) resembles that of lacustrine basin faunas from China, with a minimum ichnogeneric tetrapod diversity of six, and a similar diversity of invertebrate ichnogenera. This diversity is somewhat less than the highest diversity ichnofaunas from the Lower Cretaceous of Korea, which have recently been recognized as iconic examples of lacustrine basin tetrapod ichnofaunas. The features of the Chinese and Korean lacustrine tetrapod ichnocoenoses are consistent with what has variously been called the shorebird ichnofacies, or the shorebird ichnocoenosis within the *Grallator* ichnofacies. These shorebird ichnofaunas have many dominant elements in common with others known from East Asia and globally and offer opportunities for comparative analyses of lake basin ichnofaunas, ichnocoenoses and ichnofacies. The Tugulu Group assemblage is one of the best studied Lower Cretaceous ichnofaunas in the world. The comprehensive review and re-analysis of faunal composition and ecology extends our knowledge and understanding of other terrestrial ecosystems from this epoch.

## Supplemental Information

10.7717/peerj.11476/supp-1 Supplemental Information 1 Comparison table of previous MGCM specimen numbers and current YLSNHM specimens numbers.

10.7717/peerj.11476/supp-2 Supplemental Information 2 Measurements (in centimeters and degrees) of Tetrapoda tracks from from the Wuerhe area in Xinjiang, China.

## Institutional and locality abbreviations

MGCM: Moguicheng Dinosaur and Bizarre Stone Museum, Xinjiang, China
YLSNHM: Yingliang Stone Natural History Museum, Shuitou, Fujiang Province, China
HYQ: Huangyangquan site, Wuerhe District, Karamay City, Xinjiang

## Additional Information and Declarations

### Data Availability

The following information was supplied regarding data availability: The raw measurements are available in the Supplemental File. All of the three 3D models of this article are available at https://www.morphosource.org/. *Eubrontes* sp. HYQ-E-11 DOI 10.17602/M2/M361400. Pterosaur tracks HYQ-7 DOI 10.17602/M2/M361405. Pterosaur tracks ylsnhm00974 DOI 10.17602/M2/M361411.
